# Imidazo[1,2-*a*]quinoxalines Derivatives Grafted with Amino Acids: Synthesis and Evaluation on A375 Melanoma Cells

**DOI:** 10.3390/molecules23112987

**Published:** 2018-11-15

**Authors:** Adrien Chouchou, Cindy Patinote, Pierre Cuq, Pierre-Antoine Bonnet, Carine Deleuze-Masquéfa

**Affiliations:** 1 IBMM, Université de Montpellier, CNRS, ENSCM, 34000 Montpellier, France; adrien.chouchou@umontpellier.fr (A.C.); cindy.patinote@umontpellier.fr (C.P.); pierre.cuq@umontpellier.fr (P.C.); carine.masquefa@umontpellier.fr (C.D.-M.); 2 Société d’Accélération du Transfert de Technologies (SATT AxLR), CSU, 950 rue Saint Priest, 34090 Montpellier, France

**Keywords:** imidazo[1,2-*a*]quinoxaline, melanoma, imiqualine, A375 structure–activity relationship

## Abstract

Imiqualines (imidazoquinoxaline derivatives) are anticancer compounds with high cytotoxic activities on melanoma cell lines. The first generation of imiqualines, with two lead compounds (EAPB0203 and EAPB0503), shows remarkable in vitro (IC_50_ = 1 570 nM and IC_50_ = 200 nM, respectively, on the A375 melanoma cell line) and in vivo activity on melanoma xenografts. The second generation derivatives, EAPB02302 and EAPB02303, are more active, with IC_50_ = 60 nM and IC_50_ = 10 nM, respectively, on A375 melanoma cell line. The aim of this study was to optimize the bioavailability of imiqualine derivatives, without losing their intrinsic activity. For that, we achieved chemical modulation on the second generation of imiqualines by conjugating amino acids on position 4. A new series of twenty-five compounds was efficiently synthesized by using microwave assistance and tested for its activity on the A375 cell line. In the new series, compounds **11a**, **9d** and **11b** show cytotoxic activities less than second generation compounds, but similar to that of the first generation ones (IC_50_ = 403 nM, IC_50_ = 128 nM and IC_50_ = 584 nM, respectively). The presence of an amino acid leads to significant enhancement of the water solubility for improved drugability.

## 1. Introduction

Cutaneous melanoma is a malignant tumour of melanocytes located in the basal epidermis. It is the most aggressive and lethal form of skin cancer because of its fast-metastatic development [1,2]. Its incidence has been increasing worldwide for several decades. While in its early stages remarkable outcomes can be achieved with surgery alone, metastatic melanoma requires therapeutic treatment intervention. Thanks to genomic studies, knowledge regarding the molecular biology of melanomas has improved, leading to recent FDA-approved therapies [3,4,5]. However, all the subtypes of the disease are not equally treated [5] and marked resistance mostly against kinase inhibitors rapidly occurs [6,7]. As the incidence among the worldwide fair-skinned population is increasing, this public health concern remains challenging.

**Scheme 1:**
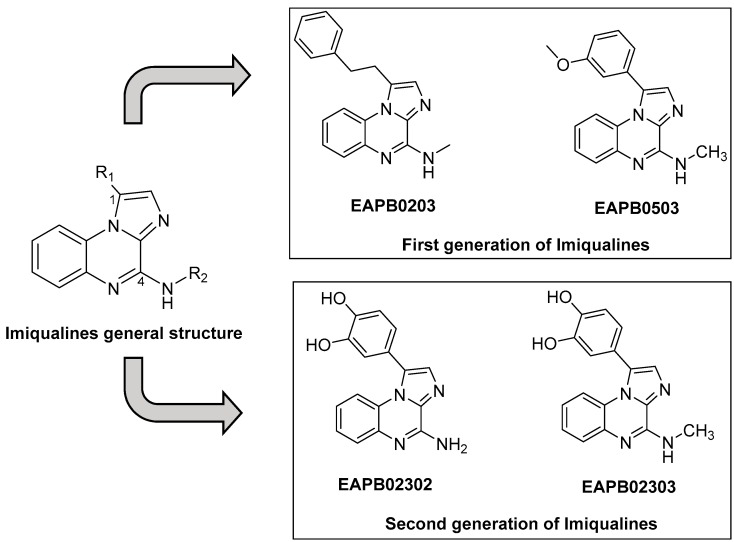
General and lead compounds structures of the imiqualines.

Our group is working on the development of the imidazo[1,2-*a*]quinoxaline derivatives presented in Scheme 1, called imiqualines, as potential antitumoral agents, particularly for the treatment of melanoma [8,9]. The first generation of imiqualines was essentially substituted on position 1 by multiple aromatic moieties directly grafted to the main structure or *via* an alkyl linker. The second generation is characterized by the presence of the 3,4-dihydroxyphenyl moiety on position 1 since the presence of such catechol residue enhances global hydrophilicity. The chemical modulations of the first hits, EAPB0203 and EAPB0503, afforded new leads EAPB02303 and its *N*-demethylated derivative EAPB02302, with impressive in vitro activities in the nanomolar range on the A375 human melanoma cancer cell line [10,11].

EAPB02302 and EAPB02303 are considered as promising anticancer agents but exhibit high lipophilicity (cLogP values estimated at 2.68 and 3.55, respectively), which might be critical for future preclinical in vivo studies. Indeed, their low solubility in water might be a major drawback for further development, especially in the case of intravenous use. In a preliminary study [12], the pharmacokinetic parameters of EAPB02303 were determined in mice after a single intraperitoneal administration. For this, the compound was solubilized in a mixture of DMSO, Tween 80 and sodium chloride solution 0.9% (10/10/80, *v*/*v*/*v*). The use of DMSO is recognized as toxic [13], in particular when used repeatedly as would be the case in an efficacy study. In order to optimize the results of efficacy studies, we chose to chemically modulate our lead compounds to obtain more soluble compounds with a moderate impact on the cytotoxic activity. The result of the introduction of various amino acids on position 4 of the heterocycle on both the physicochemical properties and biological activity was studied. Such an approach to increase solubility, which remains a key factor for potential pharmaceutical development, has already been described in the literature [14,15,16,17]. The introduction of amino acid moieties has been showed to increase the water solubility as well as selective cytotoxicity [18,19,20]. We present herein the synthesis of new imidazo[1,2-*a*]quinoxalines decorated with a panel of natural α-amino acids and their in vitro preliminary evaluation on A375 cell line.

## 2. Results and Discussion

### 2.1. Synthesis of Imidazo[1,2-a]quinoxaline Derivatives

**Scheme 2:**
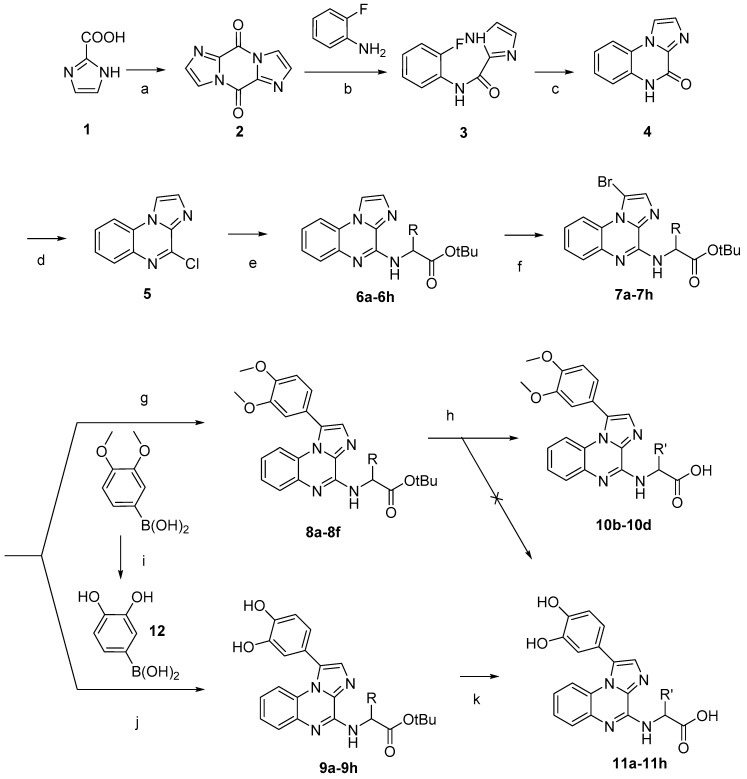
Synthesis of imidazo[1,2-*a*]quinoxaline derivatives grafted with the α-amine group of the amino acid. *Reagents and Conditions*: (**a**) SOCl_2_, reflux, 18h; (**b**) NaHMDS, THF, 0 °C to RT, 5 h; (**c**) NaH, DMA, reflux, 48 h; (**d**) DEA, POCl_3_, MW (130 °C, 15 min); (**e**) H-amino acid (PG)-OtBu, DIEA, DMF, MW (150 °C, 30–60 min); (**f**) NBS, CHCl_3_, reflux, 2 h; (**g**) 3,4-dimethoxyphenylboronic acid, Pd(PPh_3_)_4_, Na_2_CO_3_, DME, MW (140 °C, 20 min); (**h**) BBr_3_, CH_2_Cl_2_, RT, 1h–3h, (**i**) BBr_3_, CH_2_Cl_2_, RT, 1h; (**j**) 3,4-dihydroxyphenylboronic acid **12**, Pd(PPh_3_)_4_, Na_2_CO_3_, DME, MW (140 °C, 20 min); (**k**) TFA/ CH_2_Cl_2_ (1/1, *v*/*v*), RT, 1–2 h.

**Scheme 3:**
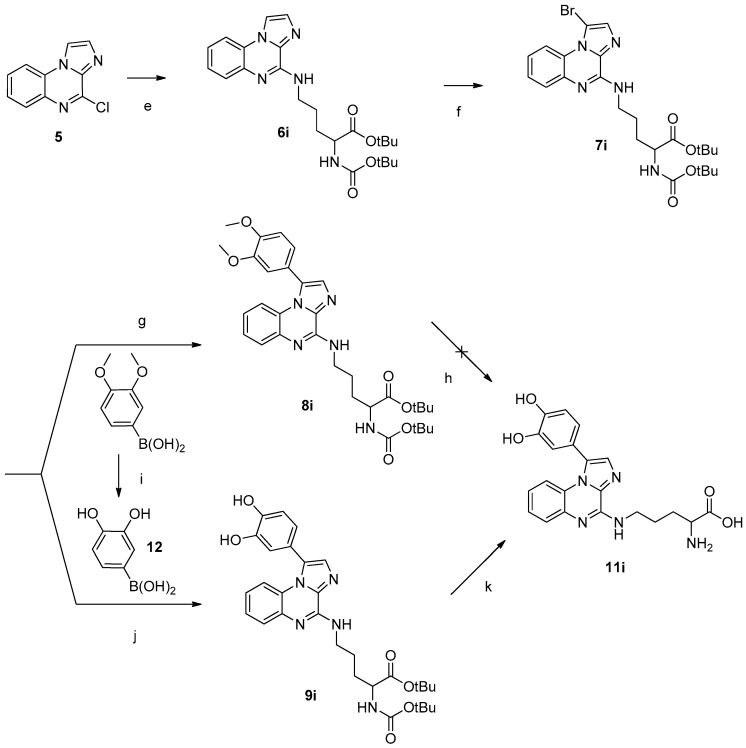
Synthesis of the ornithine-containing imidazo[1,2-*a*]quinoxaline derivative grafted by the side chain. *Reagents and Conditions*: (**e**) Boc-Orn-OtBu, HCl, DIEA, DMF, MW (150 °C, 30–60 min); (**f**) NBS, CHCl_3_, reflux, 2 h; (**g**) 3,4-dimethoxyphenylboronic acid, Pd(PPh_3_)_4_, Na_2_CO_3_, DME, MW (140 °C, 20 min); (**h**) BBr_3_, CH_2_Cl_2_, RT, 1 h–3 h; (**i**) BBr_3_, CH_2_Cl_2_, RT, 1 h; (**j**) 3,4-dihydroxyphenylboronic acid **12**, Pd(PPh_3_)_4_, Na_2_CO_3_, DME, MW (140 °C, 20 min); (**k**) TFA/ CH_2_Cl_2_ (1/1, *v*/*v*), RT, 1–2 h.

The synthetic pathways and the structures of imidazo[1,2-*a*]quinoxalines used in this study are given in Scheme 2 and Scheme 3. Intermediates **1** to **5** were synthetized thanks to a route we previously described [9,21]. Briefly, the carbonylimidazole dimer **2** results from the condensation of the 2-imidazole carboxylic acid **1** in presence of thionyl chloride. Addition of *o*-fluoroaniline to the dimer **2** gives the intermediate **3**. Cyclisation is facilitated by using sodium hydride in dimethylacetamide. Treatment of compound **4** with phosphorus oxychloride and *N,N*-diethylaniline gives the chlorinated key intermediate **5**.

The chlorine of **5** can be substituted by an amino group in the presence of diisopropylethylamine in DMF under microwave assistance. Among the commercially available amino acids, we chose to study the effect of a short or long, substituted or not, aliphatic or aryl side chain with or without a hydrophilic amino or phenolic group. The amino group could be either the α-amine of an amino acid or the amine of the side chain for the ornithine residue. The introduction of HGlyOtBu, HAlaOtBu, HValOtBu, HLeuOtBu, HLysOtBu, HOrnOtbu, HPheOtBu, HTyrOtBu is described in Scheme 2 and the grafting of BocOrnOtBu is depicted in Scheme 3. The bromination of the intermediates **6** by *N-bromosuccinimide* leads to compounds **7**. The 3,4-dimethoxyphenyl group is introduced in position 1 *via* a Suzuki-Miyaura cross-coupling reaction to furnish compounds **8a**–**8f** and **8i**. Boron tribromide did not allow the cleavage of all the protections to give the targeted compounds **11a**–**11i**, even if supplementary equivalents of BBr_3_ were added at the beginning or during the reaction, or if the reaction time was extended. Such an approach allows one to obtain compounds **10b**–**10d** as the main products. These compounds present remaining methoxy groups on the phenyl on position 1 without the protecting groups on the amino acid. The by-products correspond to one remaining methoxy group on the phenyl either at position 3′ or 4′, but unfortunately, they could not be recovered separately.

As target compounds **11a**–**11f** could not be obtained by this way, we decided to deprotect first the 3,4-dimethoxyphenylboronic acid using boron tribromide in order to obtain the 3,4-dihydroxy-phenylboronic acid **12**. This boronic acid readily reacts with intermediates **7** under microwave irradiation to furnish the hydroxylated derivatives **9a**–**9i**. A final step of deprotection of the amino acid moiety using TFA in CH_2_Cl_2_ was used to obtain the final compounds **11a**–**11i**.

### 2.2. In Vitro Cytotoxic Activity on A375 Cell Line and Calculated ClogP

All new imidazo[1,2-*a*]quinoxaline derivatives **8b**–**d**, **8f**, **9a**–**9i**, **10b**–**d** and **11a**–**11i** were tested for their in vitro antiproliferative activities on the human melanoma cell line A375. Their IC_50_ values (concentration of the compound (nM) producing 50% cell growth inhibition after 96 h of drug exposure) were determined using in vitro cytotoxicity assays and are displayed in Table 1 and Table 2.

Lipophilicity, expressed as the logarithm of a compound’s octanol/water partition coefficient (ClogP), is a physicochemical property of drugs that affects many biological mechanisms, especially drug absorption and distribution (absorption, plasma protein binding and membrane permeation) [22]. This parameter can also be correlated to solubility, metabolism and toxicity [23,24]. In a drug discovery process, compounds must be sufficiently lipophilic to cross the membrane barriers and at the same time be sufficiently water soluble to reach their targets. Therefore, a poor water solubility is a common cause of rejection during development [25]. This is why we also estimated the lipophilicity and hydrophilicity properties of all new compounds by predicting their ClogP and theoretical water solubility values thanks to fragmentation methods available on the ACDLabs^®^ software. These calculated values are purely theoretical but give a good estimate of what might be the solubility of the compounds in blood circulation (pH 7.4). This approach is used in pharmaceutical industry for the screening of new compounds [26,27]. Results of our new imidazo[1,2-*a*]quinoxaline derivatives were compared each other as well as with the first and second generation imiqualine leads as shown in Table 3. 

Compounds **8b**, **8c**, **8d** and **8f** have fully protected amino acid residues (side chain and carboxylic function) with 3,4-dimethoxy substitution on the phenyl ring in position 1. They exhibit weak IC_50_ values higher than 10,000 nM. Compounds belonging to this chemical series are very lipophilic with estimated ClogP values higher than 5 (5.25, 5.61, 6.47, 6.98, 7.05 and 7.01 for compounds **8a**, **8b**, **8c**, **8d**, **8e** and **8f**, respectively). The theoretical water solubility of compounds **8** at pH 7.4 is very low since the order varies from 5.88 × 10^−4^ to 3.43 × 10^−5^ mg/mL for compounds **8a** and **8f**, respectively. Therefore, the presence of protective groups on the amino acids and the dimethoxy groups on the phenyl appears not to be valuable, both in terms of water solubility and cytotoxic efficiency. 

Compounds **9a**–**9i** are only protected on the amino acids moieties. These compounds exhibit various cytotoxic activities with IC_50_ values ranging from 128 to 7180 nM, for **9d** and **9c**, respectively. These compounds show high lipophilicity since their ClogP values range from 4.99 to 7.99 for **9a** and **9h** respectively. Similarly, the theoretical values of water solubility are between 3.31 × 10^−5^ and 1.79 × 10^−3^ mg/mL for **9h** and **9a**, respectively. By comparing compounds **8** and **9**, we note that the replacement of the methoxy groups by the hydroxy groups induces, for some residues, an important increase of the biological activities. Nevertheless, such modification, with conservation of the amino acid protection, does not improve the theoretical water solubility of the new synthesized compounds.

Compounds **10b**–**10d** are deprotected on the amino acid residues but still present dimethoxy groups on the phenyl substitution at position 1. These compounds exhibit IC_50_ values higher than 5000 nM (IC_50_ values at 5 947 nM for **10b** and higher than 10,000 nM for **10c** and **10d**). However, ClogP values are below 5 and theoretical water solubilities are higher than 0.8 mg/mL. The transition from compounds **8** to compounds **10** provides a significant decrease of lipophilicity as well as an improvement of theoretical water solubility. The comparison of the compounds according to the grafted residue permits to highlight this improvement. Actually, ClogP values decrease from 5.61 to 3.21, from 6.47 to 4.07 and from 6.98 to 4.58 for compounds **8b** to **10b**, **8c** to **10c** and **8d** to **10d**, respectively. Even more impressive, the theoretical water solubility values increase from 3.8 × 10^−4^ to 6.01, 1.35.10^−4^ to 1.91 and 7.43 × 10^−5^ to 0.89 mg/mL for compounds **8b** to **10b**, **8c** to **10c** and **8d** to **10d**, respectively. The deprotection of the amino acid residues does not improve the cytotoxic activity but clearly improve the theoretical water solubility. The presence of dihydroxy groups on the phenyl substitution at position 1 appears to be necessary for the conservation of the cytotoxic activity.

Compounds **11a**-**11i** are fully deprotected on the amino acid residues as well as on the catechol group at the position 1. Among these compounds, five have attractive IC_50_ values below 1000 nM: 403, 584, 673, 838 and 951 for **11a**, **11b**, **11e**, **11d** and **11g**, respectively. These compounds show ClogP values ranged from 2.57 to 4.32 for **11f** and **11d**. Several compounds present very interesting theoretical water solubility: compounds **11a**, **11b**, **11c**, **11d**, **11g** and **11h** exhibit values higher than 3 mg/mL. Very high values are obtained for compounds **11a** and **11b** at 47.18 and 19.92 mg/mL, respectively. The presence of an alkyl or aryl moiety on the side chain of the amino acid residue does not decrease the water solubility for these compounds. Nevertheless, the tendency appears to not be the same for compounds **11e**, **11f** and **11i** with theoretical water solubility values less than 1 × 10^−2^ mg/mL. These compounds possess ornithine (grafted by the α-amine or by the amine of the side chain of the amino acid) or lysine residues. These two residues are therefore not valuable for increasing the water solubility of the compounds at pH 7.4.

Compounds **11a**, **11b**, **11c** and **11d** present an alkyl chain at position 4 while compounds **11g** and **11h** display an aryl chain on this same position. Saturated alkyl groups appear to be most interesting for our compounds. A trend that seems to stand out in these four compounds is that when the carbon number of the alkyl chain increases, the biological activity and the theoretical solubility decrease (except for **11c** which activity does not follow this tendency). Moreover, the presence of a phenyl group causes an increase in lipophilicity and a decrease in biological activities, in particular with the presence of a phenol (compound **11h**) on the side chain of the amino acid moiety. The compounds with lysine or ornithine residues did not show favorable results neither for biological activities or water solubility. 

The transition of compounds **9** to compounds **11** is carried out by the cleavage of all the protecting groups of the amino acid moieties. Such a deprotection step forms less lipophilic compounds (lower ClogP) with similar or higher activity for all the amino acids tested, except for compounds **11d** and **11f** with the leucine and ornithine residues grafted by the amine in the α-position, respectively. It should be noted that the presence of an ornithine amino acid residue grafted by the α-amine or the amine of the side chain of the amino acid does not result in any significant differences in the biological activity (IC_50_ values at 3404 nM for **11f** and 5168 nM for **11i**) or to the solubility (theoretical water solubility values at 5.82 × 10^−3^ and 3.05 × 10^−3^ mg/mL for **11f** and **11i**, respectively). Surprisingly, compound **11e** which present a butan-1-amine substitution on the lateral chain of the amino acid show an higher biological activity than compound **11f** with a propan-1-amine substitution on its lateral chain (IC_50_ values at 673 nM for **11e** and 3 404 nM for **11f**) with equivalent theoretical water solubilities (values of 4.34.10^−3^ and 5.82.10^−3^ mg/mL for **11e** and **11f**, respectively).

Consequently, compounds **11a**, **11b**, **11d** and **11g** in particular hold our attention, both in terms of improvement of the solubility and in terms of conservation of the biological activity. The activities of these compounds are in the same potency order as the leads of the first generation of imiqualines (IC_50_ values of 200 and 1570 nM for EAPB0503 and EAPB0203, respectively), presented in Table 3. Moreover, these new compounds show highly improved water solubility. Indeed, the values for the first generation compounds were only 2.60 × 10^−3^ and 3.46 × 10^−3^ mg/mL for EAPB0203 and EAPB0503, respectively. On the other hand, the second generation imiqualine compounds exhibit higher activities than the new compounds, with IC_50_ values at 10 and 60 nM for EAPB02303 and EAPB02302 respectively. However, the solubility values of these compounds are low, with values of 1.74 × 10^−2^ mg/mL for EAPB02303 and 4.28 × 10^−2^ mg/mL for EAPB02302.

All of these data and results allow us to put forward a preferential amino acid with a small alkyl side chain in order to get a good compromise between maintaining the activity and increasing water solubility. From the close analysis of Table 1 and Table 2, it can be observed that compounds **11a**, **11b**, **9d**, **11d**, **11e** and **11g** are the most active members of this series against the tested melanoma A375 cell line (IC_50_ values less than 1000 nM). Surprisingly, compound **9d** shows the lowest IC_50_ value among these new synthesized compounds. Among these six attractive compounds, only **11a**, **11b**, **11d**, **11e** and **11g** show a marked and considerable improvement of the theoretical water solubility.

## 3. Experimental Section

### 3.1. Chemistry

#### 3.1.1. General Information

All solvents and reagents were obtained from Sigma Aldrich Chemical Co. (Saint Louis, MO, USA), Iris Biotech GmbH (Marktredwitz, Germany), Alfa Aesar Co. (Karlsruhe, Germany), VWR (Radnor, PA, USA) and FluoroChem UK (Hadfield, UK) and used without further purification unless indicated otherwise. Silica gel chromatography was conducted with 230–400 mesh 60 Å silica gel (Sigma Aldrich Chemical Co.). The progress of reaction was monitored by TLC exposure to UV light (254 nm and 366 nm). Thin layer chromatography plates (Kieselgel 60 F254) were purchased from Merck (Darmstadt, Germany). Microwave assisted organic syntheses were performed on a Biotage Initiator 2.0 microwave system (Uppsala, Sweden). ^1^H (400 MHz) and ^13^C-NMR (100 MHz) spectra were obtained on a Brüker AC-400 spectrometer (Billerica, MA, USA). Chemical shifts are given as parts per million (ppm) using residual dimethylsulfoxide signal for protons (*δ*_DMSO_ = 2.46 ppm) and carbons (*δ*_DMSO_ = 40.00 ppm). Coupling constants are reported in Hertz (Hz). Spectral splitting partners are designed as follow: singlet (s); doublet (d); triplet (t); quartet (q); multiplet (m). Mass spectral data were obtained on a Waters Micromass Q-Tof (Milford, MA, USA) spectrometer equipped with ESI source (Laboratoires de Mesures Physiques, Plateau technique de l’Institut des Biomolecules Max Mousseron, Université de Montpellier, Montpellier, France). Mass spectra were recorded in positive mode between 50 and 1500 Da, capillary and cone tension were 3000 and 20 V, respectively. The High Resolution Mass Spectroscopy (HRMS) analyses are carried out by direct introduction on a Synapt G2-S mass spectrometer (Waters, SN: UEB205) equipped with ESI source. The mass spectra were recorded in positive mode, between 100 and 1500 Da. The capillary tension is 1000 V and the cone tension is 30 V. The source and desolvation temperature are 120 °C and 250 °C, respectively. NMR ^1^H and ^13^C spectra of all compounds are in Supplementary Materials.

#### 3.1.2. Amino Acids Grafted on 4-Chloroimidazo[1,2-*a*]quinoxaline

*Tert-butyl 2-(imidazo[1,2-a]quinoxalin-4-ylamino)acetate* (**6a**): Glycine *tert*-butyl ester hydrochloride (0.824 g, 4.9 mmol), *N*,*N*-diisopropylethylamine (1.6 mL, 9.8 mmol) and 4-chloroimidazo[1,2-*a*]quinoxaline **5** (0.5 g, 2.5 mmol) were dissolved in dimethyl-formamide (10 mL) in a microwave adapted vial and sealed. The reaction mixture was irradiated at 150 °C for 30 min. The solvent was removed under reduced pressure. The crude mixture was dissolved in ethyl acetate (100 mL) and successively washed with saturated aqueous ammonium chloride, distilled water and finally brine. The organic phase was dried on sodium sulfate, filtered and concentrated under reduced pressure. The product was purified by flash chromatography eluted with cyclohexane/ethyl acetate 80/20 to 50/50. The compound is obtained as a beige solid (42% yield). C_16_H_18_N_4_O_2_. M_W_: 298.34 g/mol. ^1^H-NMR *δ* (ppm, DMSO-*d*_6_): 1.42 (s, 9H, 3 × CH_3_ OtBu), 4.13 (d, 2H, *J* = 8 Hz, CH_2_ α), 7.31–7.35 (m, 1H, CH 7), 7.40–7.44 (m, 1H, CH 8), 7.56 (dd, 1H, *J* = 4 Hz, *J* = 8 Hz, CH 9), 7.66 (d, 1H, *J* = 4 Hz, CH 2), 7.96 (t, 1H, *J* = 8 Hz, NH), 8.13 (dd, 1H, *J* = 4 Hz, *J* = 8 Hz, CH 6), 8.64 (d, 1H, *J* = 4 Hz, CH 1). ^13^C-NMR *δ* (ppm, DMSO-*d*_6_): 28.23 (CH_3_ tBu), 43.30 (CH_2_ α), 80.85 (Cq tBu), 115.14 (CH 1), 115.91 (CH 6), 123.57 (CH 7), 124.93 (Cq 5a), 126.74 (CH 9), 126.88 (CH 8), 132.52 (CH 2), 132.71 (Cq 3a), 136.81 (Cq 9a), 147.49 (Cq 4), 169.79 (C=O). MS (ESI +, QTof, *m/z*): 299.0 [M + H]^+^.

*Tert-butyl 2-(imidazo[1,2-a]quinoxalin-4-ylamino)propanoate* (**6b**): Same procedure used for the synthesis of **6a** was employed. L-Alanine *tert*-butyl ester hydrochloride (1.339 g, 7.4 mmol), *N*,*N*-diisopropylethylamine (2.4 mL, 14.7 mmol) and 4-chloroimidazo[1,2-*a*]quinoxaline **5** (0.5 g, 2.5 mmol) were dissolved and reacted in dimethyl-formamide (10 mL). The product was purified by flash chromatography eluted with cyclohexane/ethyl acetate 80/20 to 50/50. The compound is obtained as a beige solid (48% yield). C_17_H_20_N_4_O_2_. M_W_: 312.37 g/mol. ^1^H-NMR *δ* (ppm, DMSO-*d*_6_): 1.41 (s, 9H, 3 × CH_3_ OtBu), 1.51 (d, 3H, *J* = 8 Hz, CH_3_ β), 4.56–4.63 (m, 1H, CH α), 7.33–7.36 (m, 1H, CH 7), 7.41–7.43 (m, 1H, CH 8), 7.56 (dd, 1H, *J* = 4 Hz, *J* = 8 Hz, CH 9), 7.66 (d, 1H, *J* = 4 Hz, CH 2), 7.75 (d, 1H, *J* = 4 Hz, NH), 8.13 (dd, 1H, *J* = 4 Hz, *J* = 8 Hz, CH 6), 8.64 (d, 1H, *J* = 4 Hz, CH 1). ^13^C-NMR *δ* (ppm, DMSO-*d*_6_): 17.46 (CH_3_ β), 28.14 (CH_3_ tBu), 50.22 (CH α), 80.59 (Cq tBu), 115.18 (CH 1), 115.91 (CH 6), 123.64 (CH 7), 124.93 (Cq 5a), 126.73 (CH 9), 126.88 (CH 8), 132.42 (CH 2), 132.58 (Cq 3a), 136.72 (Cq 9a), 147.03 (Cq 4), 172.84 (C=O). MS (ESI +, QTof, *m/z*): 313.2 [M + H]^+^.

*Tert-Butyl 2-(imidazo[1,2-a]quinoxalin-10-ylamino)-3-methylbutanoate* (**6c**): Using the same procedure as for the synthesis of **6a**, L-valine *tert-*butyl ester hydrochloride (2.060 g, 9.8 mmol), *N*,*N*-diisopropylethylamine (2.4 mL, 14.7 mmol) and 4-chloroimidazo[1,2-*a*]quinoxaline **5** (0.5 g, 2.5 mmol) were mixed in dimethylformamide (10 mL). The product was purified by flash chromatography eluted with cyclohexane/ethyl acetate 80/20 to 50/50. The compound is obtained as a beige solid (51% yield). C_19_H_24_N_4_O_2_. M_W_: 340.42 g/mol. ^1^H-NMR *δ* (ppm, DMSO-*d*_6_): 1.03 (d, 3H, *J* = 8 Hz, CH_3_ γ), 1.06 (d, 3H, *J* = 8 Hz, CH_3_ γ’), 1.43 (s, 9H, 3 × CH_3_ OtBu), 2.29–2.37 (m, 1H, CH β), 4.52 (t, 1H, *J* = 16 Hz, CH α), 7.15 (d, 1H, *J* = 8 Hz, NH), 7.33–7.37 (m, 1H, CH 7), 7.42–7.44 (m, 1H, CH 8), 7.59 (dd, 1H *J* = 4 Hz, *J* = 8 Hz, CH 9), 7.68 (d, 1H, *J* = 4 Hz, CH 2), 8.14 (dd, 1H, *J* = 4 Hz, *J* = 8 Hz, CH 6), 8.66 (d, 1H, *J* = 4 Hz, CH 1). ^13^C-NMR *δ* (ppm, DMSO-*d*_6_): 19.42 (CH_3_ γ, CH_3_ γ’), 28.17 (CH_3_ tBu), 30.41 (CH β), 59.76 (CH α), 81.15 (Cq tBu), 115.37 (CH 1), 115.95 (CH 6), 123.88 (CH 7), 125.00 (Cq 5a), 126.84 (CH 9), 126.95 (CH 8), 132.48 (CH 2), 132.58 (Cq 3a), 136.59 (Cq 9a), 147.29 (Cq 4), 171.59 (C=O). MS (ESI +, QTof, *m/z*): 341.0 [M + H]^+^.

*Tert-butyl 2-(imidazo[1,2-a]quinoxalin-4-ylamino)-4-methylpentanoate* (**6d**): The same as for the synthesis of **6a** was used, employing L-leucine *tert-*butyl ester hydrochloride (2.198 g, 9.8 mmol), *N*,*N*-diisopropylethylamine (2.4 mL, 14.7 mmol) and 4-chloro-imidazo[1,2-*a*]quinoxaline **5** (0.5 g, 2.5 mmol) in dimethylformamide (10 mL). The product was purified by flash chromatography eluted with cyclohexane/ethyl acetate 80/20 to 50/50. The compound is obtained as a beige solid (60% yield). C_20_H_26_N_4_O_2_. M_W_: 354.45 g/mol. ^1^H-NMR *δ* (ppm, DMSO-*d*_6_): 0.93 (d, 3H, *J* = 8 Hz, CH_3_ δ), 0.96 (d, 3H, *J* = 8 Hz, CH_3_ δ’), 1.40 (s, 9H, 3 × CH_3_ OtBu), 1.60–1.67 (m, 1H, CH_2_ β), 1.77–1.79 (m, 1H, CH γ), 1.93–1.95 (m, 1H, CH_2_ β), 4.63 (t, 1H, *J* = 4 Hz, CH α), 7.26–7.33 (m, 1H, CH 7), 7.40–7.44 (m, 1H, CH 8), 7.57 (dd, 1H, *J* = 4 Hz, *J* = 8 Hz, CH 9), 7.64 (d, 1H, *J* = 4 Hz, NH), 7.66 (d, 1H, *J* = 4 Hz, CH 2), 8.13 (dd, 1H, *J* = 4 Hz, *J* = 8 Hz, CH 6), 8.64 (d, 1H, *J* = 4 Hz, CH 1). ^13^C-NMR *δ* (ppm, DMSO-*d*_6_): 22.06 (CH_3_ δ), 23.31 (CH_3_ δ’), 25.14 (CH γ), 28.15 (CH_3_ tBu), 40.01 (CH_2_ β), 52.93 (CH α), 80.67 (Cq tBu), 115.21 (CH 1), 115.90 (CH 6), 123.64 (CH 7), 124.93 (Cq 5a), 126.76 (CH 8), 126.89 (CH 9), 132.41 (CH 2), 132.58 (Cq 3a), 136.74 (Cq 9a), 147.40 (Cq 4), 172.75 (C=O). MS (ESI +, QTof, *m/z*): 355.0 [M + H]^+^.

*Tert-butyl 6-((tert-butoxycarbonyl)amino)-2-(imidazo[1,2-a]quinoxalin-4-ylamino)hexanoate* (**6e**): The same procedure used for the synthesis of **6a** was employed with *N*-ε-*tert*-butyloxycarbonyl-L-lysine *tert*-butyl ester hydrochloride (2.565 g, 7.6 mmol), *N*,*N*-diisopropyl-ethylamine (2.4 mL, 14.7 mmol) and 4-chloroimidazo[1,2-*a*]quinoxaline **5** (0.5 g, 2.5 mmol) in dimethylformamide (10 mL). The product was purified by flash chromatography eluted with cyclohexane/ethyl acetate 80/20 to 50/50. The compound is obtained as a beige solid (32% yield). C_25_H_35_N_5_O_4_. M_W_: 469.58 g/mol. ^1^H-NMR *δ* (ppm, DMSO-*d*_6_): 1.35 (s, 9H, 3 × CH_3_ OtBu), 1.41 (s, 9H, 3 × CH_3_ OtBu), 1.43–1.49 (m, 4H, CH_2_ γ, CH_2_ δ), 1.86–1.92 (m, 2H, CH_2_ β), 2.91–2.93 (m, 2H, CH_2_ ε), 4.52–4.56 (m, 1H, CH α), 6.79 (t, 1H, *J* = 4 Hz, NH-CH_2_ ε), 7.26–7.35 (m, 1H, CH 7), 7.39–7.42 (m, 1H, CH 8), 7.52 (dd, 1H, *J* = 4 Hz, *J* = 8 Hz, CH 9), 7.64 (d, 1H, *J* = 4 Hz, NH-CH α), 7.66 (d, 1H, *J* = 4 Hz, CH 2), 8.12 (dd, 1H, *J* = 4 Hz, *J* = 8 Hz, CH 6), 8.64 (d, 1H, *J* = 4 Hz, CH 1). ^13^C-NMR *δ* (ppm, DMSO-*d*_6_): 23.52 (CH_2_ γ), 28.19 (CH_3_ tBu), 28.74 (CH_3_ tBu), 29.67 (CH_2_ δ), 30.92 (CH_2_ β), 40.35 (CH_2_ ε), 54.64 (CH α), 79.68 (Cq tBu), 80.80 (Cq tBu), 115.25 (CH 1), 115.93 (CH 6), 123.70 (CH 7), 124.98 (Cq 5a), 126.81 (CH 9), 126.92 (CH 8), 132.44 (CH 2), 132.60 (Cq 3a), 136.75 (Cq 9a), 147.35 (Cq 4), 172.41 (C=O). MS (ESI +, QTof, *m/z*): 470.0 [M + H]^+^.

*Tert-butyl 5-(((benzyloxy)carbonyl)amino)-2-(imidazo[1,2-a]quinoxalin-4-ylamino)-pentanoate* (**6f**): Using the same procedure used for the synthesis of **6a** with N-δ-carbobenzoxy-L-ornithine α-*tert*-butyl ester hydrochloride (3.702 g, 10.3 mmol), *N*,*N*-diisopropylethylamine (2.4 mL, 14.7 mmol) and 4-chloroimidazo[1,2-*a*]quinoxaline **5** (0.5 g, 2.5 mmol) in dimethylformamide (10 mL). The product was purified by flash chromatography eluted with cyclohexane/ethyl acetate 80/20 to 50/50. The compound is obtained as a beige solid (38% yield). C_27_H_31_N_5_O_4_. M_W_: 489.57 g/mol. ^1^H-NMR *δ* (ppm, 400 MHz, DMSO-*d*_6_): ^1^H-NMR *δ* (ppm, DMSO-*d*_6_): 1.29 (s, 9H, 3 × CH_3_ OtBu), 1.46–1.50 (m, 2H, CH_2_ γ), 1.79–1.84 (m, 2H, CH_2_ β), 2.94–2.98 (m, 2H, CH_2_ δ), 4.44–4.46 (m, 1H, CH α), 4.90 (s, 2H, CH_2_–Phenyl), 7.17–7.21 (m, 1H, CH 7), 7.23 (s, 1H, NH-CH_2_ δ), 7.24–7.27 (m, 5H, CH Phenyl), 7.29–7.33 (m, 1H, CH 8), 7.46 (dd, 1H, *J* = 4 Hz, *J* = 8 Hz, CH 9), 7.52 (d, 1H, *J* = 4 Hz, NH-CH α), 7.56 (d, 1H, *J* = 4 Hz, CH 2), 8.02 (dd, 1H, *J* = 4 Hz, *J* = 8 Hz, CH 6), 8.53 (d, 1H, *J* = 4 Hz, CH 1). ^13^C-NMR *δ* (ppm, DMSO-*d*_6_): 26.63 (CH_2_ γ), 28.13 (CH_3_ tBu), 28.66 (CH_2_ β), 40.71 (CH_2_ δ), 54.50 (CH α), 65.67 (CH_2_-Phenyl), 80.91 (Cq tBu), 115.30 (CH 1), 115.99 (CH 6), 123.77 (CH 7), 125.03 (Cq 5a), 126.84 (CH 9), 126.97 (CH 8), 128.86 (CH Phenyl), 132.50 (CH 2), 132.65 (Cq 3a), 136.77 (Cq 9a), 147.36 (Cq 4), 156.65 (Cq Phenyl), 172.28 (C=O). MS (ESI +, QTof, *m/z*): 490.0 [M + H]^+^.

*Tert-Butyl 2-(imidazo[1,2-a]quinoxalin-4-ylamino)-3-phenylpropanoate* (**6g**):The same procedure as for the synthesis of **6a** was used with L-phenylalanine *tert*-butyl ester hydrochloride (1.9 g, 7.4 mmol), *N*,*N*-diisopropylethylamine (2.4 mL, 14.7 mmol) and 4-chloroimidazo[1,2-*a*]quinoxaline **5** (0.5 g, 2.5 mmol) in dimethylformamide (10 mL). The product was purified by flash chromatography eluted with cyclohexane/ethyl acetate 80/20 to 50/50. The compound is obtained as a beige solid (37% yield). C_23_H_24_N_4_O_2_. M_W_: 388.46 g/mol. ^1^H-NMR *δ* (ppm, DMSO-*d*_6_): 1.33 (s, 9H, 3 × CH_3_ OtBu), 3.19–3.23 (m, 1H, CH_2_ β), 3.32–3.36 (m, 1H, CH_2_ β), 4.82–4.84 (m, 1H, CH α), 7.21 (t, 1H, *J* = 8 Hz, CH Phenyl), 7.29 (t, 2H, *J* = 8 Hz, 2 × CH Phenyl), 7.32–7.34 (m, 3H, CH 7, 2 × CH Phenyl), 7.41–7.44 (m, 1H, CH 8), 7.57 (dd, 1H, *J* = 4 Hz, *J* = 8 Hz, CH 9), 7.61 (d, 1H, *J* = 8 Hz, NH), 7.66 (d, 1H, *J* = 4 Hz, CH 2), 8.13 (dd, 1H, *J* = 8, 4 Hz, CH 6), 8.64 (d, 1H, *J* = 4 Hz, CH 1). ^13^C-NMR *δ* (ppm, DMSO-*d*_6_): 26.97 (CH_3_ tBu), 35.91 (CH_2_ β), 54.93 (CH α), 79.88 (Cq tBu), 114.18 (CH 1), 114.85 (CH 6), 122.74 (CH 7), 123.89 (Cq 5a), 125.74 (CH 9), 125.84 (CH Phenyl), 125.86 (CH 8), 127.59 (CH Phenyl), 128.69 (CH Phenyl), 131.41 (CH 2), 135.54 (Cq 3a), 137.05 (Cq 9a), 146.02 (Cq 4), 170.53 (C=O). MS (ESI +, QTof, *m/z*): 389.0 [M + H]^+^.

*Tert-butyl 3-(4-(tert-butoxy)phenyl)-2-(imidazo[1,2-a]quinoxalin-4-ylamino)propanoate* (**6h**): The same procedure as for the synthesis of **6a** was used with *O*-*tert*-butyl-L-tyrosine *tert*-butyl ester hydrochloride (1.6 g, 4.9 mmol), *N*,*N*-diisopropylethylamine (2.4 mL, 14.7 mmol) and 4-chloroimidazo[1,2-*a*]quinoxaline **5** (0.5 g, 2.5 mmol) in dimethylformamide (10 mL). The product was purified by flash chromatography eluted with cyclohexane/ethyl acetate 80/20 to 50/50. The compound is obtained as a beige solid (26% yield). C_27_H_32_N_4_O_3_. M_W_: 460.57 g/mol. ^1^H-NMR *δ* (ppm, DMSO-*d*_6_): 1.23 (s, 9H, 3 × CH_3_ OtBu), 1.31 (s, 9H, 3 × CH_3_ OtBu), 3.11–3.17 (m, 1H, CH_2_ β), 3.25–3.31 (m, 1H, CH_2_ β), 4.78–4.83 (m, 1H, CH α), 6.86 (dd, 2H, *J* = 4 Hz, *J* = 8 Hz, CH Phenyl), 7.22 (dd, 2H, *J* = 4 Hz, *J* = 8 Hz, CH Phenyl), 7.32–7.36 (m, 1H, CH 7), 7.40–7.44 (m, 1H, CH 8), 7.57 (dd, 1H, *J* = 4 Hz, *J* = 8 Hz, CH 9), 7.61 (d, 1H, *J* = 4 Hz, NH), 7.66 (d, 1H, *J* = 4 Hz, CH 2), 8.13 (dd, 1H, *J* = 4 Hz, *J* = 8 Hz, CH 6), 8.64 (d, 1H, *J* = 4 Hz, CH 1). ^13^C-NMR *δ* (ppm, DMSO-*d*_6_): 28.02 (CH_3_ tBu), 28.93 (CH_3_ tBu), 36.63 (CH_2_ β), 56.09 (CH α), 78.11 (Cq tBu), 80.87 (Cq tBu), 115.22 (CH 1), 115.90 (CH 6), 123.80 (CH 7), 123.98 (2 × CH Phenyl), 124.96 (Cq 5a), 126.79 (CH 9), 126.90 (CH 8), 130.31 (2 × CH Phenyl), 132.47 (CH 2), 132.61 (Cq 3a), 136.60 (Cq 9a), 147.04 (Cq 4), 154.03 (2 × Cq Phenyl), 171.72 (C=O). MS (ESI +, QTof, *m/z*): 461.2 [M + H]^+^.

*Tert-butyl 6-((tert-butoxycarbonyl)amino)-2-(imidazo[1,2-a]quinoxalin-4-ylamino)hexanoate* (**6i**): Same procedure used for the synthesis of **6a** was employed with *N*-α-*tert-*butyloxycarbonyl-L-ornithine *tert*-butyl ester hydrochloride (1.596 g, 4.9 mmol), *N*,*N*-diisopropyl-ethylamine (1.6 mL, 9.8 mmol) and 4-chloroimidazo[1,2-*a*]quinoxaline **5** (0.5 g, 2.5 mmol) in dimethylformamide (10 mL). The product was purified by flash chromatography eluted with cyclohexane/ethyl acetate 80/20 to 50/50. The compound is obtained as a beige solid (48% yield). C_24_H_33_N_5_O_4_. M_W_: 455.56 g/mol. ^1^H-NMR *δ* (ppm, DMSO-*d*_6_): 1.35 (s, 9H, CH-COOtBu), 1.41 (s, 9H, NH-COOtBu), 1.85–1.96 (m, 2H, CH_2_ β), 2.91–2.95 (m, 2H, CH_2_ γ), 3.35–3.39 (m, 2H, CH_2_ δ), 4.51–4.56 (m, 1H, CH α), 6.79 (d, 1H, *J* = 4 Hz, NH-CH α), 7.32–7.35 (m, 1H, CH 7), 7.39–7.42 (m, 1H, CH 8), 7.56 (dd, 1H, *J* = 4 Hz, *J* = 8 Hz, CH 9), 7.60 (d, 1H, *J* = 4 Hz, NH-CH_2_ δ), 7.66 (s, 1H, CH 2), 8.13 (dd, 1H, *J* = 4 Hz, *J* = 8 Hz, CH 6), 8.64 (d, 1H, CH 1). ^13^C-NMR *δ* (ppm, DMSO-*d*_6_): 25.87 (CH_2_ β), 28.04 (CH_3_ tBu), 28.67 (CH_3_ tBu), 28.43 (CH_2_ γ), 39.79 (CH_2_ δ), 54.75 (CH α), 78.45 (Cq tBu), 80.53 (Cq tBu), 115.02 (CH 1), 115.79 (CH 6), 122.99 (CH 7), 124.65 (Cq 5a), 126.50 (CH 9), 126.73 (CH 8), 132.20 (CH 2), 132.87 (Cq 3a), 147.78 (Cq 4), 156.00 (Cq 9a), 172.33 (C=O). MS (ESI +, QTof, *m/z*): 456.0.1 [M + H]^+^.

#### 3.1.3. Bromination

*Tert-butyl 2-((1-bromoimidazo[1,2-a]quinoxalin-4-yl)amino)acetate* (**7a**): A solution of **6a** (0.26 g, 0.87 mmol) and *N-bromosuccinimide* (0.19 g, 1.0 mmol) in CHCl_3_ (50 mL) was heated under reflux for 2 h. The solvent was removed under reduced pressure. The crude mixture was dissolved in ethyl acetate (50 mL) and successively washed with saturated aqueous ammonium chloride, saturated aqueous sodium bicarbonate, distilled water and finally brine (50 mL). The organic phase was dried on sodium sulfate, filtered and concentrated under reduced pressure. The compound was obtained as a beige oil (97% yield) and used without purification. C_16_H_17_BrN_4_O_2_. M_W_: 377.24 g/mol. ^1^H-NMR *δ* (ppm, DMSO-*d*_6_): 1.42 (s, 9H, 3 × CH_3_ OtBu), 4.12 (d, 2H, *J* = 8 Hz, CH_2_ α), 7.34–7.37 (m, 1H, CH 7), 7.45–7.50 (m, 1H, CH 8), 7.59 (dd, 1H, *J* = 4 Hz, *J* = 8 Hz, CH 9), 7.76 (s, 1H, CH 2), 8.02 (t, 1H, *J* = 8 Hz, NH), 8.96 (dd, 1H, *J* = 4 Hz, *J* = 8 Hz, CH 6). ^13^C-NMR *δ* (ppm, DMSO-*d*_6_): 28.22 (CH_3_ tBu), 43.26 (CH_2_ α), 80.93 (Cq tBu), 99.61 (Cq 1), 115.12 (CH 6), 123.08 (CH 7), 126.08 (Cq 5a), 127.35 (CH 9), 127.78 (CH 8), 134.01 (Cq 3a), 134.79 (CH 2), 137.63 (Cq 9a), 147.05 (Cq 4), 169.64 (C=O). MS (ESI +, QTof, *m/z*): 377.0 [M + H]^+^.

*Tert-butyl 2-((1-bromoimidazo[1,2-a]quinoxalin-4-yl)amino)propanoate* (**7b**): A solution of **6b** (0.3 g, 0.96 mmol) and *N-bromosuccinimide* (0.2 g, 1.1 mmol) in CHCl_3_ (50 mL) was heated under reflux for 2 h. The solvent was removed under reduced pressure. The crude mixture was dissolved in ethyl acetate (50 mL) and successively washed with saturated aqueous ammonium chloride, saturated aqueous sodium bicarbonate, distilled water and finally brine. The organic phase was dried on sodium sulfate, filtered and concentrated under reduced pressure. The compound was obtained as a beige oil (98% yield) and used without purification. C_17_H_19_BrN_4_O_2_. M_W_: 391.26 g/mol. ^1^H-NMR *δ* (ppm, DMSO-*d*_6_): 1.41 (s, 9H, 3 × CH_3_ OtBu), 1.51 (d, 3H, *J* = 8 Hz, CH_3_ β), 4.55–4.59 (m, 1H, CH α), 7.34–7.38 (m, 1H, CH 7), 7.46–7.48 (m, 1H, CH 8), 7.60 (dd, 1H, *J* = 4 Hz, *J* = 8 Hz, CH 9), 7.76 (s, 1H, CH 2), 7.82 (d, 1H, *J* = 4 Hz, NH), 8.97 (dd, 1H, *J* = 4 Hz, *J* = 8 Hz, CH 6). ^13^C-NMR *δ* (ppm, DMSO-*d*_6_): 17.40 (CH_3_ β), 28.03 (CH_3_ tBu), 50.24 (CH α), 80.70 (Cq tBu), 99.68 (CH 1), 115.06 (CH 6), 123.17 (CH 7), 126.06 (Cq 5a), 127.30 (CH 9), 127.45 (CH 8), 133.87 (Cq 3a), 134.73 (CH 2), 137.47 (Cq 9a), 146.58 (Cq 4), 172.66 (C=O). MS (ESI +, QTof, *m/z*): 391.1 [M + H]^+^.

*Tert-b*utyl 2-((1-*bromoimidazo*[1,2-a]quinoxalin-4-yl)amino)-3-methylbutanoate (**7c**): A solution of **6c** (0.38 g, 1.1 mmol) and *N-bromosuccinimide* (0.24 g, 1.4 mmol) in CHCl_3_ (50 mL) was heated under reflux for 2 h. The solvent was removed under reduced pressure. The crude mixture was dissolved in ethyl acetate (50 mL) and successively washed with saturated aqueous ammonium chloride, saturated aqueous sodium bicarbonate, distilled water and finally brine. The organic phase was dried on sodium sulfate, filtered and concentrated under reduced pressure. The compound was obtained as a beige oil (96% yield) and used without purification. C_19_H_23_BrN_4_O_2_. M_W_: 419.31 g/mol. ^1^H-NMR *δ* (ppm, DMSO-*d*_6_): 1.02 (d, 3H, *J* = 8 Hz, CH_3_ γ), 1.05 (d, 3H, *J* = 8 Hz, CH_3_ γ’), 1.43 (s, 9H, 3 × CH_3_ OtBu), 2.31–2.36 (m, 1H, CH β), 4.49 (t, 1H, *J* = 16 Hz, CH α), 7.20 (d, 1H, *J* = 8 Hz, NH), 7.35–7.40 (m, 1H, CH 7), 7.47–7.49 (m, 1H, CH 8), 7.62 (dd, 1H, *J* = 4 Hz, *J* = 8 Hz, CH 9), 7.76 (s, 1H, CH 2), 8.97 (dd, 1H, *J* = 4 Hz, *J* = 8 Hz, CH 6). ^13^C-NMR *δ* (ppm, DMSO-*d*_6_): 19.42 (CH_3_ γ, CH_3_ γ’), 28.17 (CH_3_ tBu), 30.40 (CH β), 59.76 (CH α), 81.25 (Cq tBu), 99.86 (Cq 1), 115.08 (CH 6), 123.38 (CH 7), 126.16 (Cq 5a), 127.44 (CH 9), 127.47 (CH 8), 133.81 (Cq 3a), 134.74 (CH 2), 137.40 (Cq 9a), 146.84 (Cq 4), 171.44 (C=O). MS (ESI +, QTof, *m/z*): 419.1 [M + H]^+^.

*Tert-butyl 2-((1-bromoimidazo[1,2-a]quinoxalin-4-yl)amino)-4-methylpentanoate* (**7d**): A solution of **6d** (0.35 g, 1.0 mmol) and *N-bromosuccinimide* (0.2 g, 1.2 mmol) in CHCl_3_ (50 mL) was heated under reflux for 2 h. The solvent was removed under reduced pressure. The crude mixture was dissolved in ethyl acetate (50 mL) and successively washed with saturated aqueous ammonium chloride, saturated aqueous sodium bicarbonate, distilled water and finally brine. The organic phase was dried on sodium sulfate, filtered and concentrated under reduced pressure. The compound was obtained as a beige oil (95% yield) and used without purification. C_20_H_25_BrN_4_O_2_. M_W_: 433.34 g/mol. ^1^H-NMR *δ* (ppm, DMSO-*d*_6_): 0.91 (d, 3H, *J* = 8 Hz, CH_3_ δ), 0.95 (d, 3H, *J* = 8 Hz, CH_3_ δ’), 1.40 (s, 9H, 3 × CH_3_ OtBu), 1.60–1.62 (m, 1H, CH_2_ β), 1.77–1.79 (m, 1H, CH γ), 1.94–1.96 (m, 1H, CH_2_ β), 4.60 (t, 1H, *J* = 4 Hz, CH α), 7.35–7.38 (m, 1H, CH 7), 7.46–7.50 (m, 1H, CH 8), 7.60 (dd, 1H, *J* = 4 Hz, *J* = 8 Hz, CH 9), 7.71 (d, 1H, *J* = 4 Hz, NH), 7.76 (s, 1H, CH 2), 8.97 (dd, 1H, *J* = 4 Hz, *J* = 8 Hz, CH 6). ^13^C-NMR *δ* (ppm, DMSO-*d*_6_): 22.03 (CH_3_ δ), 23.31 (CH_3_ δ’), 25.12 (CH γ), 28.14 (CH_3_ tBu), 40.10 (CH_2_ β), 52.95 (CH α), 80.76 (Cq tBu), 99.21 (Cq 1), 115.07 (CH 6), 123.17 (CH 7), 126.08 (Cq 5a), 127.37 (CH 9), 127.46 (CH 8), 133.88 (Cq 3a), 134.71 (CH 2), 137.55 (Cq 9a), 146.97 (Cq 4), 172.59 (C=O). MS (ESI +, QTof, *m/z*): 433.1 [M + H]^+^.

*Tert-butyl 2-((1-bromoimidazo[1,2-a]quinoxalin-4-yl)amino)-6-((Tert-butoxycarbonyl)amino)-hexanoate* (**7e**): A solution of **6e** (1.2 g, 2.5 mmol) and *N-bromosuccinimide* (0.54 g, 3.1 mmol) in CHCl_3_ (50 mL) was heated under reflux for 2 h. The solvent was removed under reduced pressure. The crude mixture was dissolved in ethyl acetate (50 mL) and successively washed with saturated aqueous ammonium chloride, saturated aqueous sodium bicarbonate, distilled water and finally brine. The organic phase was dried on sodium sulfate, filtered and concentrated under reduced pressure. The compound was obtained as a beige oil (78% yield) and used without purification. C_25_H_34_BrN_5_O_4_. M_W_: 548.47 g/mol. ^1^H-NMR *δ* (ppm, DMSO-*d*_6_): 1.22–1.24 (m, 2H, CH_2_ γ), 1.34 (s, 9H, 3 × CH_3_ OtBu), 1.40 (s, 9H, 3 × CH_3_ OtBu), 1.54–1.56 (m, 2H, CH_2_ β), 1.89–1.91 (m, 2H, CH_2_ δ), 2.83–2.89 (m, 2H, CH_2_ ε), 4.13–4.19 (m, 1H, CH α), 6.78 (t, 1H, *J* = 4 Hz, NH-CH_2_ ε), 7.34–7.39 (m, 1H, CH 7), 7.46–7.48 (m, 1H, CH 8), 7.65 (dd, 1H, *J* = 4 Hz, *J* = 8 Hz, CH 9), 8.03 (s, 1H, CH 2), 8.34 (d, 1H, *J* = 4 Hz, NH-CH α), 8.97 (dd, 1H, *J* = 4 Hz, *J* = 8 Hz, CH 6). ^13^C-NMR *δ* (ppm, DMSO-*d*_6_): 22.90 (CH_2_ γ), 28.15 (CH_3_ tBu), 28.73 (CH_3_ tBu), 29.46 (CH_2_ δ), 31.34 (CH_2_ β), 39.36 (CH_2_ ε), 54.61 (CH α), 80.85 (Cq tBu), 81.14 (Cq tBu), 99.70 (Cq 1), 115.06 (CH 6), 123.19 (CH 7), 126.09 (Cq 5a), 127.39 (CH 9), 127.46 (CH 8), 133.87 (CH 2), 134.70 (Cq 3a), 137.54 (Cq 9a), 146.88 (Cq 4), 171.39 (C=O), 172.21 (C=O). MS (ESI +, QTof, *m/z*): 548.1 [M + H]^+^.

*Tert-butyl 5-(((benzyloxy)carbonyl)amino)-2-((1-bromoimidazo[1,2-a]quinoxalin-4-yl)-amino)pentanoate* (**7f**): A solution of **6f** (0.45 g, 0.9 mmol) and *N*-bromosuccinimide (0.20 g, 1.1 mmol) in CHCl_3_ (50 mL) was heated under reflux for 2 h. The solvent was removed under reduced pressure. The crude mixture was dissolved in ethyl acetate (50 mL) and successively washed with saturated aqueous ammonium chloride, saturated aqueous sodium bicarbonate, distilled water and finally brine. The organic phase was dried on sodium sulfate, filtered and concentrated under reduced pressure. The compound was obtained as a beige oil (94% yield) and used without purification. C_27_H_30_BrN_5_O_4_. M_W_: 568.46 g/mol. ^1^H-NMR *δ* (ppm, DMSO-*d*_6_): 1.40 (s, 9H, 3 × CH_3_ OtBu), 1.51–1.59 (m, 2H, CH_2_ γ), 1.89–1.92 (m, 2H, CH_2_ β), 3.03–3.08 (m, 2H, CH_2_ δ), 4.52–4.54 (m, 1H, CH α), 5.00 (s, 2H, CH_2_-Phenyl), 7.26 (s, 1H, NH-CH_2_ δ), 7.31–7.35 (m, 5H, CH Phenyl), 7.36–7.39 (m, 1H, CH 7), 7.46–7.50 (m, 1H, CH 8), 7.59 (dd, 1H, *J* = 4 Hz, *J* = 8 Hz, CH 9), 7.67 (d, 1H, *J* = 4 Hz, NH-CH α), 7.76 (s, 1H, CH 2), 8.99 (dd, 1H, *J* = 4 Hz, *J* = 8 Hz, CH 6). ^13^C-NMR *δ* (ppm, DMSO-*d*_6_): 26.53 (CH_2_ γ), 28.13 (CH_3_ tBu), 28.50 (CH_2_ β), 40.42 (CH_2_ δ), 54.43 (CH α), 65.59 (CH_2_-Phenyl), 80.92 (Cq tBu), 99.69 (Cq 1), 115.07 (CH 6), 123.20 (CH 7), 126.10 (Cq 5a), 127.39 (CH 9), 127.45 (CH 8), 128.18 (CH Phenyl), 128.78 (CH Phenyl), 133.87 (Cq 3a), 134.70 (CH 2), 137.72 (Cq 9a), 146.85 (Cq 4), 156.56 (Cq Phenyl), 172.05 (C=O). MS (ESI +, QTof, *m/z*): 568.3 [M + H]^+^.

*Tert-butyl 2-((1-bromoimidazo[1,2-a]quinoxalin-4-yl)amino)-3-phenylpropanoate* (**7g**): A solution of **6g** (0.34 g, 0.9 mmol) and *N*-bromosuccinimide (0.18 g, 1.1 mmol) in CHCl_3_ (50 mL) was heated under reflux for 2 h. The solvent was removed under reduced pressure. The crude mixture was dissolved in ethyl acetate (50 mL) and successively washed with saturated aqueous ammonium chloride, saturated aqueous sodium bicarbonate, distilled water and finally brine. The organic phase was dried on sodium sulfate, filtered and concentrated under reduced pressure. The compound was obtained as a beige oil (83% yield) and used without purification. C_23_H_23_BrN_4_O_2_. M_W_: 467.36 g/mol. ^1^H-NMR *δ* (ppm, DMSO-*d*_6_): 1.33 (s, 9H, 3 × CH_3_ OtBu), 3.18–3.22 (m, 1H, CH_2_ β), 3.31–3.34 (m, 1H, CH_2_ β), 4.79–4.81 (m, 1H, CH α), 7.21 (t, 1H, *J* = 8 Hz, CH Phenyl), 7.27–7.30 (m, 2H, 2 × CH Phenyl), 7.32–7.34 (m, 2H, 2xCH Phenyl), 7.35–7.38 (m, 1H, CH 7), 7.46–7.49 (m, 1H, CH 8), 7.60 (dd, 1H, *J* = 4 Hz, *J* = 8 Hz, CH 9), 7.65 (d, 1H, *J* = 8 Hz, NH), 7.75 (s, 1H, CH 2), 8.97 (dd, 1H, *J* = 2 Hz, *J* = 8 Hz, CH 6). ^13^C-NMR *δ* (ppm, DMSO-*d*_6_): 28.04 (CH_3_ tBu), 36.91 (CH_2_ β), 56.01 (CH α), 81.06 (Cq tBu), 99.76 (Cq 1), 115.07 (CH 6), 123.33 (CH 7), 126.13 (Cq 5a), 126.96 (CH Phenyl), 127.45 (CH 9), 127.47 (CH 8), 128.68 (CH Phenyl), 129.77 (CH Phenyl), 133.80 (Cq Phenyl), 134.76 (CH 2), 137.45 (Cq 3a), 138.05 (Cq 9a), 146.66 (Cq 4), 171.45 (C=O). MS (ESI +, QTof, m/z): 467.1 [M + H]^+^.

*Tert-butyl 2-((1-bromoimidazo[1,2-a]quinoxalin-4-yl)amino)-3-(4-(Tert-butoxy)phenyl)-propanoate* (**7h**): A solution of **6h** (0.34 g, 0.9 mmol) and *N*-bromosuccinimide (0.18 g, 1.1 mmol) in CHCl_3_ (50 mL) was heated under reflux for 2 h. The solvent was removed under reduced pressure. The crude mixture was dissolved in ethyl acetate (50 mL) and successively washed with saturated aqueous ammonium chloride, saturated aqueous sodium bicarbonate, distilled water and finally brine. The organic phase was dried on sodium sulfate, filtered and concentrated under reduced pressure. The compound was obtained as a beige oil (91% yield) and used without purification. C_23_H_23_BrN_4_O_2_. M_W_: 467.36 g/mol. ^1^H-NMR *δ* (ppm, DMSO-*d*_6_): 1.22 (s, 9H, 3 × CH_3_ OtBu), 1.31 (s, 9H, 3 × CH_3_ OtBu), 3.11–3.16 (m, 1H, CH_2_ β), 3.24–3.26 (m, 1H, CH_2_ β), 4.76–4.81 (m, 1H, CH α), 6.85 (dd, 2H, *J* = 4 Hz, *J* = 8 Hz, CH Phenyl), 7.20 (dd, 2H, *J* = 4 Hz, *J* = 8 Hz, CH Phenyl), 7.34–7.38 (m, 1H, CH 7), 7.46–7.50 (m, 1H, CH 8), 7.59 (dd, 1H, *J* = 4 Hz, *J* = 8 Hz, CH 9), 7.65 (d, 1H, *J* = 8 Hz, NH), 7.75 (d, 1H, *J* = 4 Hz, CH 2), 8.98 (dd, 1H, *J* = 4 Hz, *J* = 8 Hz, CH 6). ^13^C-NMR *δ* (ppm, DMSO-*d*_6_): 28.04 (CH_3_ tBu), 28.93 (CH_3_ tBu), 36.61 (CH_2_ β), 56.03 (CH α), 78.10 (Cq tBu), 81.01 (Cq tBu), 99.71 (Cq 1), 115.08 (CH 6), 123.32 (CH 7), 123.96 (2 × CH Phenyl), 126.13 (Cq 5a), 127.42 (CH 9), 127.47 (CH 8), 130.33 (2 × CH Phenyl), 132.54 (Cq 3a), 134.78 (CH 2), 137.43 (Cq 9a), 146.61 (Cq 4), 154.06 (2 × Cq Phenyl), 171.53 (C=O). MS (ESI +, QTof, m/z): 467.1 [M + H]^+^.

*Tert-butyl 5-((1-bromoimidazo[1,2-a]quinoxalin-4-yl)amino)-2-((Tert-butoxycarbonyl)amino)-pentanoate* (**7i**): A solution of **6i** (0.49 g, 1.1 mmol) and *N*-bromosuccinimide (0.23 g, 1.3 mmol) in CHCl_3_ (50 mL) was heated under reflux for 2 h. The solvent was removed under reduced pressure. The crude mixture was dissolved in ethyl acetate (50 mL) and successively washed with saturated aqueous ammonium chloride, saturated aqueous sodium bicarbonate, distilled water and finally brine. The organic phase was dried on sodium sulfate, filtered and concentrated under reduced pressure. The compound was obtained as a beige oil (92% yield) and used without purification. C_24_H_32_BrN_5_O_4_. M_W_: 534.45 g/mol. ^1^H-NMR *δ* (ppm, DMSO-*d*_6_): 1.44 (s, 9H, CH-COOtBu), 1.46 (s, 9H, NH-COOtBu), 1.79–1.83 (m, 2H, CH_2_ β), 1.91–1.94 (m, 2H, CH_2_ γ), 3.73–3.78 (m, 2H, CH_2_ δ), 4.25–4.29 (m, 1H, CH α), 5.39 (d, 1H, *J* = 4 Hz, NH-CH α), 6.33 (d, 1H, *J* = 4 Hz, NH-CH_2_ δ), 7.27–7.31 (m, 1H, CH 7), 7.41–7.45 (m, 1H, CH 8), 7.48 (s, 1H, CH 2), 7.81 (dd, 1H, *J* = 4 Hz, *J* = 8 Hz, CH 9), 8.98 (dd, 1H, *J* = 4 Hz, *J* = 8 Hz, CH 6). ^13^C-NMR *δ* (ppm, DMSO-*d*_6_): 25.74 (CH_2_ β), 28.07 (CH_3_ tBu), 28.45 (CH_3_ tBu), 30.17 (CH_2_ γ), 40.42 (CH_2_ δ), 54.00 (CH α), 79.75 (Cq tBu), 81.98 (Cq tBu), 99.05 (Cq 1), 115.02 (CH 6), 122.77 (CH 7), 124.54 (Cq 5a), 127.12 (CH 8), 127.98 (CH 9), 134.22 (CH 2), 135.98 (Cq 3a), 146.86 (Cq 4), 155.59 (Cq 9a), 171.92 (C=O). MS (ESI +, QTof, *m/z*): 534.1 [M + H]^+^.

#### 3.1.4. Suzuki-Miyaura Cross-Coupling Reactions

*Tert-butyl 2-((1-(3,4-dimethoxyphenyl)imidazo[1,2-a]quinoxalin-4-yl)amino)acetate* (**8a**): To a mixture of **7a** (0.270 g, 0.71 mmol) in DME/H_2_O (2/1, 15 mL) were added 3,4-dimethoxyphenylboronic acid (0.143 g, 0.79 mmol), tetrakis(triphenylphosphine) palladium (0.042 g, 0.036 mmol) and sodium carbonate (0.152 g, 1.43 mmol) in a microwave-adapted vial. The reaction was submitted to microwave irradiations during 20 min at 150 °C and then filtered on a Celite pad. The filtrate was concentrated under reduced pressure and successively washed with saturated aqueous ammonium chloride, saturated aqueous sodium bicarbonate, distilled water and finally brine. The organic phase was dried on sodium sulfate, filtered and concentrated under reduced pressure. The product was purified by flash chromatography eluted with cyclohexane/ethyl acetate 80/20 to 50/50. The compound is obtained as a white solid (19% yield). C_24_H_26_N_4_O_4_. MW: 434.49 g/mol. ^1^H-NMR *δ* (ppm, DMSO-*d*_6_): 1.44 (s, 9H, 3 × CH_3_ OtBu), 3.74 (s, 3H, OCH_3_ Phenyl), 3.87 (s, 3H, OCH_3_ Phenyl), 4.14 (d, 2H, *J* = 8 Hz, CH_2_ α), 7.01–7.05 (m, 1H, CH 7), 7.11 (d, 1H, *J* = 4 Hz, CH Phenyl), 7.14 (s, 1H, *J* = 4 Hz, CH Phenyl), 7.18 (d, 1H, *J* = 4 Hz, CH Phenyl), 7.31 (dd, 1H, *J* = 4 Hz, *J* = 8 Hz, CH 6), 7.33–7.35 (m, 1H, CH 8), 7.54 (s, 1H, CH 2), 7.56 (dd, 1H, *J* = 4 Hz, *J* = 8 Hz, CH 9), 7.96 (t, 1H, *J* = 8 Hz, NH). ^13^C-NMR *δ* (ppm, DMSO-*d*_6_): 28.26 (CH_3_ tBu), 43.33 (CH_2_ α), 80.86 (Cq tBu), 112.31 (CH Phenyl), 114.25 (CH Phenyl), 115.93 (CH 6), 122.75 (CH 7), 123.30 (CH Phenyl), 126.03 (Cq 5a), 126.58 (CH 8), 127.20 (CH 9), 132.66 (CH 2), 133.141 (Cq 3a), 130.93 (Cq 1), 137.71 (Cq 9a), 149.27 (Cq Phenyl), 150.14 (Cq Phenyl), 147.74 (Cq 4), 169.85 (C=O). MS (ESI +, QTof, *m/z*): 435.1 [M + H]^+^.

*Tert-butyl 2-((1-(3,4-dimethoxyphenyl)imidazo[1,2-a]quinoxalin-4-yl)amino)propanoate* (**8b**): Following the same procedure used for the synthesis of **8a**, to a mixture of **7b** (0.440 g, 1.12 mmol) in DME/H_2_O (2/1, 15 mL) were added 3,4-dimethoxyphenylboronic acid (0.225 g, 1.24 mmol), tetrakis(triphenylphosphine) palladium (0.065 g, 0.056 mmol) and sodium carbonate (0.237 g, 2.24 mmol) in a microwave-adapted vial. The product was purified by flash chromatography eluted with cyclohexane/ethyl acetate 80/20 to 50/50. The compound is obtained as a white solid (42% yield). C_25_H_28_N_4_O_4_. MW: 448.51 g/mol. ^1^H-NMR *δ* (ppm, DMSO-*d*_6_): 1.32 (s, 9H, 3 × CH_3_ OtBu), 1.42 (d, 3H, *J* = 8 Hz, CH_3_ β), 3.64 (s, 3H, OCH_3_ Phenyl), 3.76 (s, 3H, OCH_3_ Phenyl), 4.48–4.52 (m, 1H, CH α), 6.90–6.95 (m, 1H, CH 7), 7.01 (d, 1H, *J* = 4 Hz, CH Phenyl), 7.03 (s, 1H, CH Phenyl), 7.08 (d, 1H, *J* = 4 Hz, CH Phenyl), 7.20 (dd, 1H, *J* = 4 Hz, *J* = 8 Hz, CH 6), 7.22–7.25 (m, 1H, CH 8), 7.42 (s, 1H, CH 2), 7.46 (dd, 1H, *J* = 4 Hz, *J* = 8 Hz, CH 9), 7.82 (d, 1H, *J* = 4 Hz, NH). ^13^C-NMR *δ* (ppm, DMSO-*d*_6_): 17.59 (CH_3_ β), 28.25 (CH_3_ tBu), 50.30 (CH α), 80.72 (Cq tBu), 112.40 (CH Phenyl), 114.33 (CH Phenyl), 116.00 (CH 6), 122.77 (CH 7), 122.90 (Cq 1), 123.37 (CH Phenyl), 126.10 (CH 8), 126.65 (Cq 5a), 127.28 (CH 9), 131.05 (Cq 3a), 132.63 (CH 2), 137.68 (Cq 9a), 147.33 (Cq 4), 149.36 (Cq Phenyl), 150.22 (Cq Phenyl), 172.96 (C=O). MS (ESI +, QTof, m/z): 449.2 [M + H]^+^. HRMS calculated for C_25_H_29_N_4_O_4_ 449.2189, found 449.2186.

*Tert-butyl 2-((1-(3,4-dimethoxyphenyl)imidazo[1,2-a]quinoxalin-4-yl)amino)-3-methylbutanoate* (**8c**): Followigng the same procedure used for the synthesis of **8a**, to a mixture of **7c** (0.370 g, 0.88 mmol) in DME/H_2_O (2/1, 15 mL) were added 3,4-dimethoxyphenylboronic acid (0.176 g, 0.97 mmol), tetrakis(triphenylphosphine) palladium (0.051 g, 0.044 mmol) and sodium carbonate (0.186 g, 1.76 mmol) in a microwave-adapted vial. The product was purified by flash chromatography eluted with cyclohexane/ethyl acetate 80/20 to 50/50. The compound is obtained as a white solid (69% yield). C_27_H_32_N_4_O_4_. MW: 476.57 g/mol. ^1^H-NMR *δ* (ppm, DMSO-*d*_6_): 0.95 (d, 3H, *J* = 8 Hz, CH_3_ γ), 0.98 (d, 3H, *J* = 8 Hz, CH_3_ γ’), 1.34 (s, 9H, 3 × CH_3_ OtBu), 2.20–2.28 (m, 1H, CH β), 3.63 (s, 3H, OCH_3_ Phenyl), 3.77 (s, 3H, OCH_3_ Phenyl), 4.44 (t, 1H, *J* = 16 Hz, CH α), 6.92–6.97 (m, 1H, CH 7), 7.00 (s, 1H, CH Phenyl), 7.02 (d, 1H, *J* = 4 Hz, NH), 7.06 (d, 1H, *J* = 4 Hz, CH Phenyl), 7.08 (d, 1H, *J* = 4 Hz, CH Phenyl), 7.22 (dd, 1H, *J* = 4 Hz, *J* = 8 Hz, CH 6), 7.23–7.26 (m, 1H, CH 8), 7.42 (s, 1H, CH 2), 7.49 (dd, 1H, *J* = 4 Hz, *J* = 8 Hz, CH 9). ^13^C-NMR *δ* (ppm, DMSO-*d*_6_): 19.43 (CH_3_ γ’), 19.56 (CH_3_ γ), 28.28 (CH_3_ tBu), 30.57 (CH β), 56.15 (OCH_3_ Phenyl), 56.24 (OCH_3_ Phenyl), 59.73 (CH α), 81.31 (Cq tBu), 112.41 (CH Phenyl), 114.35 (CH Phenyl), 116.02 (CH 6), 122.67 (Cq 1), 123.14 (CH 7), 123.39 (CH Phenyl), 126.19 (Cq 5a), 126.71 (CH 8), 127.39 (CH 9), 131.25 (Cq Phenyl), 132.68 (CH 2), 133.00 (Cq 3a), 137.55 (Cq 9a), 147.56 (Cq 4), 149.36 (Cq Phenyl), 150.26 (Cq Phenyl), 171.70 (C=O). MS (ESI +, QTof, *m/z*): 477.1 [M + H]^+^. HRMS calculated for C_27_H_33_N_4_O_4_ 477.2502, found 477.2505.

*Tert-butyl 2-((1-(3,4-dimethoxyphenyl)imidazo[1,2-a]quinoxalin-4-yl)amino)-4-methyl-pentanoate*: (**8d**): Following the same procedure used for the synthesis of **8a**, to a mixture of **7d** (0.360 g, 0.83 mmol) in DME/H_2_O (2/1, 15 mL) were added 3,4-dimethoxyphenylboronic acid (0.166 g, 0.91 mmol), tetrakis(triphenylphosphine) palladium (0.048 g, 0.041 mmol) and sodium carbonate (0.176 g, 1.66 mmol) in a microwave-adapted vial. The product was purified by flash chromatography eluted with cyclohexane/ethyl acetate 80/20 to 50/50. The compound is obtained as a white solid (61% yield). C_28_H_34_N_4_O_4_. MW: 490.59 g/mol. ^1^H-NMR *δ* (ppm, DMSO-*d*_6_): 0.83 (d, 3H, *J* = 8 Hz, CH_3_ δ), 0.86 (d, 3H, *J* = 8 Hz, CH_3_ δ’), 1.31 (s, 9H, 3 × CH_3_ OtBu), 1.55–1.58 (m, 1H, CH_2_ β), 1.67–1.70 (m, 1H, CH γ), 1.84–1.88 (m, 1H, CH_2_ β), 3.63 (s, 3H, OCH_3_ Phenyl), 3.76 (s, 3H, OCH_3_ Phenyl), 4.55 (t, 1H, *J* = 4 Hz, CH α), 6.90–6.94 (m, 1H, CH 7), 7.01 (d, 1H, *J* = 4 Hz, CH Phenyl), 7.05 (s, 1H, CH Phenyl), 7.08 (d, 1H, *J* = 4 Hz, CH Phenyl), 7.20 (dd, 1H, *J* = 4 Hz, *J* = 8 Hz, CH 6), 7.22–7.24 (m, 1H, CH 8), 7.41 (s, 1H, CH 2), 7.45 (dd, 1H, *J* = 4 Hz, *J* = 8 Hz, CH 9), 7.50 (d, 1H, *J* = 4 Hz, NH). ^13^C-NMR *δ* (ppm, DMSO-*d*_6_): 22.14 (CH_3_ δ), 23.41 (CH_3_ δ’), 25.25 (CH γ), 28.25 (CH_3_ tBu), 40.29 (CH_2_ β), 52.96 (CH α), 56.14 (OCH_3_ Phenyl), 56.23 (OCH_3_ Phenyl), 80.79 (Cq tBu), 112.40 (CH Phenyl), 114.34 (CH Phenyl), 115.99 (CH 6), 122.78 (Cq 1), 122.88 (CH 7), 123.36 (CH Phenyl), 126.11 (Cq 5a), 126.69 (CH 8), 127.29 (CH 9), 131.09 (Cq 3a), 132.61 (CH 2), 137.70 (Cq 9a), 147.66 (Cq 4), 149.36 (Cq Phenyl), 150.23 (Cq Phenyl), 172.86 (C=O). MS (ESI +, QTof, *m/z*): 491.0 [M + H]^+^. HRMS calculated for C_28_H_35_N_4_O_4_ 491.2658, found 491.2654.

*Tert-butyl 6-((Tert-butoxycarbonyl)amino)-2-((1-(3,4-dimethoxyphenyl)imidazo[1,2-a]-quinoxalin-4-yl)amino)hexanoate* (**8e**): Following the same procedure used for the synthesis of **8a**, to a mixture of **7e** (1.090 g, 1.99 mmol) in DME/H_2_O (2/1, 15 mL) were added 3,4-dimethoxyphenylboronic acid (0.397 g, 2.18 mmol), tetrakis(triphenylphosphine) palladium (0.114 g, 0.099 mmol) and sodium carbonate (0.421 g, 3.97 mmol) in a microwave-adapted vial. The product was purified by flash chromatography eluted with cyclohexane/ethyl acetate 80/20 to 50/50. The compound is obtained as a white solid (7% yield). C_33_H_43_N_5_O_6_. MW: 605.72 g/mol. ^1^H-NMR *δ* (ppm, DMSO-*d*_6_): 1.36 (s, 9H, 3 × CH_3_ OtBu), 1.41–1.46 (m, 13H, CH_2_ γ, 3 × CH_3_ OtBu, CH_2_ δ), 1.88–1.91 (m, 2H, CH_2_ β), 2.93–2.95 (m, 2H, CH_2_ ε), 3.73 (s, 3H, OCH_3_ Phenyl), 3.87 (s, 3H, OCH_3_ Phenyl), 4.54–4.58 (m, 1H, CH α), 6.80 (t, 1H, *J* = 4 Hz, NH-CH_2_ ε), 7.01–7.05 (m, 1H, CH 7), 7.11 (d, 1H, *J* = 4 Hz, CH Phenyl), 7.15 (s, 1H, CH Phenyl), 7.18 (d, 1H, *J* = 4 Hz, CH Phenyl), 7.31 (dd, 1H, *J* = 4 Hz, *J* = 8 Hz, CH 6), 7.31–7.35 (m, 1H, CH 8), 7.52 (s, 1H, CH 2), 7.55 (dd, 1H, *J* = 4 Hz, *J* = 8 Hz, CH 9), 7.59 (d, 1H, *J* = 4 Hz, NH-CH α). ^13^C-NMR *δ* (ppm, DMSO-*d*_6_): 23.49 (CH_2_ γ), 28.18 (CH_3_ tBu), 28.72 (CH_3_ tBu), 29.64 (CH_2_ δ), 30.94 (CH_2_ β), 40.40 (CH_2_ ε), 54.56 (CH α), 56.05 (OCH_3_ Phenyl), 56.14 (OCH_3_ Phenyl), 77.78 (Cq tBu), 80.80 (Cq tBu), 112.30 (CH Phenyl), 114.22 (CH Phenyl), 115.91 (CH 6), 122.67 (CH 7), 123.28 (CH Phenyl), 126.03 (Cq 5a), 126.56 (CH 8), 127.23 (CH 9), 131.01 (Cq 1), 132.53 (CH 2), 132.98 (Cq 3a), 137.61 (Cq 9a), 147.51 (Cq 4), 149.27 (Cq Phenyl), 150.14 (Cq Phenyl), 156.04 (Cq Phenyl), 172.41 (C=O). MS (ESI +, QTof, *m/z*): 606.2 [M + H]^+^. HRMS calculated for C_33_H_44_N_5_O_6_ 606.3292, found 606.3291.

*Tert-butyl 5-(((benzyloxy)carbonyl)amino)-2-((1-(3,4-dimethoxyphenyl)imidazo[1,2-a]-quinoxalin-4-yl)amino)pentanoate* (**8f**): Following the same procedure used for the synthesis of **8a**, to a mixture of **7f** (0.470 g, 0.89 mmol) in DME/H_2_O (2/1, 15 mL) were added 3,4-dimethoxyphenylboronic acid (0.165 g, 0.91 mmol), tetrakis(triphenylphosphine) palladium (0.048 g, 0.041 mmol) and sodium carbonate (0.175 g, 1.65 mmol) in a microwave-adapted vial. The product was purified by flash chromatography eluted with cyclohexane/ethyl acetate 80/20 to 50/50. The compound is obtained as a white solid (42% yield). C_35_H_39_N_5_O_6_. MW: 625.71 g/mol. ^1^H-NMR *δ* (ppm, DMSO-*d*_6_): ^1^H-NMR *δ* (ppm, 400 MHz, DMSO-*d*_6_): 1.52 (s, 9H, 3 × CH_3_ OtBu), 1.77–1.81 (m, 2H, CH_2_ γ), 1.99–2.03 (m, 1H, CH_2_ δ), 2.10–2.13 (m, 1H, CH_2_ δ), 3.34–3.39 (m, 2H, CH_2_ β), 3.88 (s, 3H, OCH_3_ Phenyl), 4.02 (s, 3H, OCH_3_ Phenyl), 5.12 (s, 2H, CH_2_-Phenyl), 5.37–5.39 (m, 1H, CH α), 6.99 (d, 2H, *J* = 4 Hz, CH Phenyl), 7.03 (s, 1H, CH Phenyl), 7.10–7.12 (m, 1H, CH 7), 7.28 (dd, 1H, *J* = 4 Hz, *J* = 8 Hz, CH 6), 7.32–7.35 (m, 7H, CH 8, CH2, CH Phenyl), 7.36 (dd, 1H, *J* = 4 Hz, *J* = 8 Hz, CH 9), 7.47 (d, 1H, *J* = 4 Hz, NH-CH_2_ δ), 7.76 (dd, 1H, *J* = 4 Hz, *J* = 8 Hz, NH-CH α). ^13^C-NMR *δ* (ppm, DMSO-*d*_6_): 25.62 (CH_2_ γ), 28.09 (CH_3_ tBu), 30.04 (CH_2_ β), 40.61 (CH_2_ δ), 54.42 (CH α), 66.58 (CH_2_-Phenyl), 82.42 (Cq tBu), 111.30 (CH Phenyl), 113.24 (CH Phenyl), 114.69 (Cq 1), 115.99 (CH 6), 123.16 (CH 7), 126.40 (Cq 5a), 126.82 (CH 9), 126.87 (CH 8), 128.01 (CH Phenyl), 128.08 (CH Phenyl), 129.49 (CH Phenyl), 131.85 (CH 2), 132.87 (Cq 3a), 136.68 (Cq 9a), 149.16 (Cq 4), 150.12 (Cq Phenyl), 152.37 (Cq Phenyl), 156.51 (Cq Phenyl), 171.59 (C=O). MS (ESI +, QTof, *m/z*): 626.0 [M + H]^+^. HRMS calculated for C_35_H_40_N_5_O_6_ 626.2979, found 626.2982.

*Tert-butyl 2-((Tert-butoxycarbonyl)amino)-5-((1-(3,4-dimethoxyphenyl)imidazo[1,2-a]-quinoxalin-4-yl)amino)pentanoate* (**8i**): Following the same procedure used for the synthesis of **8a**, to a mixture of **7i** (0.515 g, 0.96 mmol) in DME/H_2_O (2/1, 15 mL) were added 3,4-dimethoxyphenylboronic acid (0.193 g, 1.06 mmol), tetrakis(triphenylphosphine) palladium (0.056 g, 0.048 mmol) and sodium carbonate (0.204 g, 1.93 mmol) in a microwave-adapted vial. The product was purified by flash chromatography eluted with cyclohexane/ethyl acetate 80/20 to 50/50. The compound is obtained as a white solid (45% yield). C_32_H_41_N_5_O_6_. MW: 591.70 g/mol. ^1^H-NMR *δ* (ppm, DMSO-*d*_6_): 1.34 (s, 9H, CH-COOtBu), 1.39 (s, 9H, NH-COOtBu), 1.73–1.74 (m, 2H, CH_2_ β), 1.77–1.78 (m, 2H, CH_2_ γ), 3.56–3.59 (m, 2H, CH_2_ δ), 3.74 (s, 3H, OCH_3_ Phenyl), 3.87 (s, 3H, OCH_3_ Phenyl), 3.89–3.92 (m, 1H, CH α), 6.95–7.00 (m, 1H, CH 7), 7.09 (dd, 1H, *J* = 4 Hz, *J* = 8 Hz, CH Phenyl), 7.14 (s, 1H, CH Phenyl), 7.15 (dd, 1H, *J* = 4 Hz, *J* = 8 Hz, CH Phenyl), 7.19 (d, 1H, *J* = 4 Hz, NH-CH α), 7.28 (dd, 1H, *J* = 4 Hz, *J* = 8 Hz, CH 6), 7.30–7.32 (m, 1H, CH 8), 7.47 (s, 1H, CH 2), 7.58 (dd, 1H, *J* = 4 Hz, *J* = 8 Hz CH 9), 7.72 (t, 1H, *J* = 4 Hz, NH-CH_2_ δ). ^13^C-NMR *δ* (ppm, DMSO-*d*_6_): 25.97 (CH_2_ β), 28.06 (CH_3_ tBu), 28.25 (CH_3_ tBu), 28.67 (CH_2_ γ), 39.79 (CH_2_ δ), 54.77 (CH α), 56.05 (OCH_3_ Phenyl), 56.15 (OCH_3_ Phenyl), 78.46 (Cq tBu), 80.54 (Cq tBu), 112.32 (CH Phenyl), 114.22 (CH Phenyl), 115.83 (CH 6), 122.12 (CH 7), 122.82 (CH Phenyl), 123.26 (Cq 1), 125.75 (Cq 5a), 126.41 (CH 8), 127.07 (CH 9), 132.32 (CH 2), 138.24 (Cq 3a), 148.04 (Cq 4), 149.26 (Cq Phenyl), 150.10 (Cq Phenyl), 156.01 (Cq 9a), 172.36 (C=O). MS (ESI +, QTof, *m/z*): 592.1 [M + H]^+^. HRMS calculated for C_32_H_42_N_5_O_6_ 592.3135, found 592.3137.

*Tert-butyl 2-((1-(3,4-dihydroxyphenyl)imidazo[1,2-a]quinoxalin-4-yl)amino)acetate* (**9a**): To a mixture of **7a** (0.320 g, 0.85 mmol) in DME/H_2_O (2/1, 15 mL) were added compound **12** (0.392 g, 2.54 mmol), tetrakis(triphenylphosphine) palladium (0.049 g, 0.040 mmol) and sodium carbonate (0.179 g, 1.70 mmol) in a microwave-adapted vial. The reaction was submitted to microwave irradiations during 20 min at 150 °C and then filtered on a Celite pad. The filtrate was concentrated under reduced pressure and successively washed with saturated aqueous ammonium chloride, saturated aqueous sodium bicarbonate, distilled water and finally brine. The organic phase was dried on sodium sulfate, filtered and concentrated under reduced pressure. The product was purified by flash chromatography eluted with cyclohexane/ethyl acetate 80/20 to 50/50. The compound is obtained as a white solid (16% yield). C_22_H_22_N_4_O_4_. MW: 406.43 g/mol. 1H-NMR δ (ppm, DMSO-*d*_6_): ^1^H-NMR *δ* (ppm, DMSO-*d*_6_): 1.42 (s, 9H, 3 × CH_3_ OtBu), 4.15 (d, 2H, *J* = 8 Hz, CH_2_ α), 6.82 (dd, 1H, *J* = 4 Hz, *J* = 8 Hz, CH Phenyl), 6.89 (dd, 1H, *J* = 4 Hz, *J* = 8 Hz, CH Phenyl), 6.93 (s, 1H, CH Phenyl), 7.02–7.06 (m, 1H, CH 7), 7.33 (dd, 1H, *J* = 4 Hz, *J* = 8 Hz, CH 6), 7.35–7.38 (m, 1H, CH 8), 7.46 (s, 1H, CH 2), 7.54 (dd, 1H, *J* = 4 Hz, *J* = 8 Hz, CH 9), 7.96–8.00 (m, 1H, NH), 9.32 (s, 1H, C-OH Phenyl), 9.41 (s, 1H, C-OH Phenyl). ^13^C-NMR *δ* (ppm, DMSO-*d*_6_): 28.23 (CH_3_ tBu), 43.30 (CH_2_ α), 80.85 (Cq tBu), 115.37 (Cq 1), 115.92 (CH 6), 116.48 (CH 7), 117.89 (CH Phenyl), 121.13 (CH Phenyl), 122.06 (CH 8), 122.81 (CH Phenyl), 125.98 (Cq 5a), 126.54 (CH 9), 127.77 (Cq Phenyl), 131.43 (Cq 3a), 132.36 (CH 2), 132.89 (Cq 9a), 146.04 (Cq Phenyl), 147.13 (Cq Phenyl), 147.65 (Cq 4), 169.76 (C=O). MS (ESI +, QTof, *m/z*): 407.2 [M + H]^+^. HRMS calculated for C_22_H_23_N_4_O_4_ 407.1706, found 407.1710.

*Tert-butyl 2-((1-(3,4-dihydroxyphenyl)imidazo[1,2-a]quinoxalin-4-yl)amino)propanoate* (**9b**): Following the same procedure used for the synthesis of **9a** (see 4.1.4.8.), to a mixture of **7b** (0.290 g, 0.74 mmol) in DME/H_2_O (2/1, 15 mL) were added compound **12** (0.342 g, 2.22 mmol), tetrakis- (triphenylphosphine) palladium (0.043 g, 0.037 mmol) and sodium carbonate (0.157 g, 1.48 mmol) in a microwave-adapted vial. The product was purified by flash chromatography eluted with cyclohexane/ethyl acetate 80/20 to 50/50. The compound is obtained as a white solid (6% yield). C_23_H_24_N_4_O_4_. MW: 420.46 g/mol. ^1^H-NMR *δ* (ppm, DMSO-*d*_6_): 1.42 (s, 9H, 3 × CH_3_ OtBu), 1.51 (d, 3H, *J* = 8 Hz, CH_3_ β), 4.59–4.63 (m, 1H, CH α), 6.81 (d, 1H, *J* = 4 Hz, CH Phenyl), 6.90 (d, 1H, *J* = 4 Hz, CH Phenyl), 6.93 (s, 1H, CH Phenyl), 7.01–7.05 (m, 1H, CH 7), 7.30 (dd, 1H, *J* = 4 Hz, *J* = 8 Hz, CH 6), 7.33–7.37 (m, 1H, CH 8), 7.45 (s, 1H, CH 2), 7.53 (dd, 1H, *J* = 4 Hz, *J* = 8 Hz, CH 9), 7.66 (d, 1H, *J* = 4 Hz, NH), 9.36 (s, 2H, C-OH Phenyl). ^13^C-NMR *δ* (ppm, DMSO-*d*_6_): 17.57 (CH_3_ β), 28.16 (CH_3_ tBu), 50.17 (CH α), 80.66 (Cq tBu), 115.87 (CH 8), 116.47 (CH Phenyl), 117.88 (CH Phenyl), 121.16 (Cq 1), 122.05 (CH Phenyl), 122.78 (CH 7), 126.02 (Cq 5a), 127.16 (CH 9), 131.35 (Cq 3a), 132.19 (CH 2), 132.80 (Cq 9a), 137.57 (Cq 4), 146.03 (Cq Phenyl), 147.10 (Cq Phenyl), 147.23 (Cq Phenyl), 172.87 (C=O). MS (ESI +, QTof, *m/z*): 421.2 [M + H]^+^. HRMS calculated for C_23_H_25_N_4_O_4_ 421.1876, found 421.1875.

*Tert-butyl 2-((1-(3,4-dihydroxyphenyl)imidazo[1,2-a]quinoxalin-4-yl)amino)-3-methyl-butanoate* (**9c**): Following the same procedure used for the synthesis of **9a** (see 4.1.4.8.), to a mixture of **7c** (0.380 g, 0.91 mmol) in DME/H_2_O (2/1, 15 mL) were added compound **12** (0.340 g, 2.72 mmol), tetrakis- (triphenylphosphine) palladium (0.052 g, 0.045 mmol) and sodium carbonate (0.192 g, 1.81 mmol) in a microwave-adapted vial. The product was purified by flash chromatography eluted with cyclohexane/ethyl acetate 80/20 to 50/50. The compound is obtained as a white solid (32% yield). C_25_H_28_N_4_O_4_. MW: 448.51 g/mol. ^1^H-NMR *δ* (ppm, DMSO-*d*_6_): 1.03 (d, 3H, *J* = 8 Hz, CH_3_ γ), 1.06 (d, 3H, *J* = 8 Hz, CH_3_ γ’), 1.44 (s, 9H, 3 × CH_3_ OtBu), 2.31–2.35 (m, 1H, CH β), 4.53 (t, 1H, *J* = 8 Hz, CH α), 6.81 (dd, 1H, *J* = 4 Hz, *J* = 8 Hz, CH Phenyl), 6.90 (d, 1H, *J* = 4 Hz, *J* = 8 Hz, CH Phenyl), 6.93 (d, 1H, *J* = 4 Hz, *J* = 8 Hz, CH Phenyl), 7.03–7.05 (m, 1H, CH 7), 7.07 (d, 1H, *J* = 4 Hz, NH), 7.34–7.35 (m, 1H, CH 8), 7.37 (dd, 1H, *J* = 4 Hz, *J* = 8 Hz, CH 6), 7.46 (s, 1H, CH 2), 7.56 (dd, 1H, *J* = 4 Hz, *J* = 8 Hz, CH 9), 9.32 (s, 1H, C-OH Phenyl), 9.41 (s, 1H, C-OH Phenyl). ^13^C-NMR *δ* (ppm, DMSO-*d*_6_): 19.30 (CH_3_ γ’), 19.47 (CH_3_ γ), 28.19 (CH_3_ tBu), 30.54 (CH β), 59.60 (CH α), 81.25 (Cq tBu), 115.90 (CH 6), 116.47 (CH Phenyl), 117.89 (CH Phenyl), 121.05 (Cq 1), 122.07 (CH Phenyl), 123.07 (CH 7), 126.11 (Cq 5a), 126.56 (CH 8), 127.28 (CH 9), 131.55 (Cq Phenyl), 132.24 (CH 2), 132.72 (Cq 3a), 137.43 (Cq 9a), 146.03 (Cq 4), 147.14 (Cq Phenyl), 147.47 (Cq Phenyl), 171.62 (C=O). MS (ESI +, QTof, *m/z*): 449.3 [M + H]^+^. HRMS calculated for C_25_H_29_N_4_O_4_ 449.2189, found 449.2188.

*Tert-butyl 2-((1-(3,4-dihydroxyphenyl)imidazo[1,2-a]quinoxalin-4-yl)amino)-4-methyl-pentanoate* (**9d**): Following the same procedure used for the synthesis of **9a** (see 4.1.4.8.), to a mixture of **7d** (0.460 g, 1.06 mmol) in DME/H_2_O (2/1, 15 mL) were added compound **12** (0.400 g, 2.6 mmol), tetrakis- (triphenylphosphine) palladium (0.062 g, 0.053 mmol) and sodium carbonate (0.224 g, 2.11 mmol) in a microwave-adapted vial. The product was purified by flash chromatography eluted with cyclohexane/ethyl acetate 80/20 to 50/50. The compound is obtained as a white solid (16% yield). C_26_H_30_N_4_O_4_. MW: 462.54 g/mol. ^1^H-NMR *δ* (ppm, DMSO-*d*_6_): 0.93 (d, 3H, *J* = 8 Hz, CH_3_ δ), 0.97 (d, 3H, *J* = 8 Hz, CH_3_ δ’), 1.42 (s, 9H, 3 × CH_3_ OtBu), 1.56–1.60 (m, 1H, CH_2_ β), 1.79–1.83 (m, 1H, CH γ), 1.91–1.95 (m, 1H, CH_2_ β), 4.65 (t, 1H, *J* = 4 Hz, CH α), 6.81 (d, 1H, *J* = 4 Hz, CH Phenyl), 6.86 (s, 1H, CH Phenyl), 6.90 (d, 1H, *J* = 4 Hz, CH Phenyl), 7.01–7.05 (m, 1H, CH 7), 7.28–7.32 (m, 1H, CH 8), 7.34 (dd, 1H, *J* = 4 Hz, *J* = 8 Hz, CH 6), 7.46 (s, 1H, CH 2), 7.55 (dd, 1H, *J* = 4 Hz, *J* = 8 Hz, CH 9), 7.59 (d, 1H, *J* = 4 Hz, NH), 9.38 (s, 2H, C-OH Phenyl). ^13^C-NMR *δ* (ppm, DMSO-*d*_6_): 22.09 (CH_3_ δ), 23.32 (CH_3_ δ’), 25.17 (CH γ), 28.18 (CH_3_ tBu), 39.60 (CH_2_ β), 52.88 (CH α), 80.76 (Cq tBu), 115.87 (CH 6), 116.48 (CH Phenyl), 117.90 (CH Phenyl), 121.15 (Cq 1), 122.18 (CH Phenyl), 122.79 (CH 7), 126.11 (Cq 5a), 125.80 (CH 8), 126.04 (CH 9), 131.42 (Cq 3a), 132.18 (CH 2), 137.58 (Cq 9a), 146.06 (Cq 4), 147.14 (Cq Phenyl), 147.57 (Cq Phenyl), 172.77 (C=O). MS (ESI +, QTof, *m/z*): 463.2 [M + H]^+^. HRMS calculated for C_26_H_31_N_4_O_4_ 463.2345, found 463.2343.

*Tert-butyl 6-((Tert-butoxycarbonyl)amino)-2-((1-(3,4-dihydroxyphenyl)imidazo[1,2-a]-quinoxalin-4-yl)amino)hexanoate* (**9e**): Following the same procedure used for the synthesis of **9a** (see 4.1.4.8.), to a mixture of **7e** (0.242 g, 0.44 mmol) in DME/H_2_O (2/1, 15 mL) were added compound **12** (0.170 g, 1.10 mmol), tetrakis- (triphenylphosphine) palladium (0.025 g, 0.022 mmol) and sodium carbonate (0.093 g, 0.88 mmol) in a microwave-adapted vial. The product was purified by flash chromatography eluted with cyclohexane/ethyl acetate 80/20 to 50/50. The compound is obtained as a white solid (40% yield). C_31_H_39_N_5_O_6_. MW: 577.67 g/mol. ^1^H-NMR *δ* (ppm, DMSO-*d*_6_): 1.35 (s, 9H, 3 × CH_3_ OtBu), 1.40–1.42 (m, 2H, CH_2_ γ), 1.43 (s, 9H, 3 × CH_3_ OtBu), 1.45–1.47 (m, 2H, CH_2_ δ), 1.88–2.00 (m, 2H, CH_2_ β), 2.92–2.94 (m, 2H, CH_2_ ε), 4.55–4.56 (m, 1H, CH α), 6.79 (t, 1H, *J* = 4 Hz, NH-CH_2_ ε), 6.83 (d, 1H, *J* = 4 Hz, CH Phenyl), 6.89 (s, 1H, CH Phenyl), 6.93 (d, 1H, *J* = 4 Hz, CH Phenyl), 7.02–7.05 (m, 1H, CH 7), 7.31–7.33 (m, 1H, CH 8), 7.36 (dd, 1H, *J* = 4 Hz, *J* = 8 Hz, CH 6), 7.45 (s, 1H, CH 2), 7.54 (dd, 1H, *J* = 4 Hz, *J* = 8 Hz, CH 9), 7.57 (d, 1H, *J* = 4 Hz, NH-CH α), 9.33 (s, 1H, C-OH Phenyl), 9.41 (s, 1H, C-OH Phenyl). ^13^C-NMR *δ* (ppm, DMSO-*d*_6_): 22.40 (CH_2_ γ), 27.11 (CH_3_ tBu), 27.65 (CH_3_ tBu), 28.56 (CH_2_ δ), 29.91 (CH_2_ β), 39.25 (CH_2_ ε), 53.44 (CH α), 76.70 (Cq tBu), 79.75 (Cq tBu), 114.79 (CH 6), 115.40 (CH Phenyl), 116.80 (CH Phenyl), 120.07 (CH Phenyl), 121.73 (CH 7), 124.97 (Cq 5a), 125.42 (CH 8), 126.12 (CH 9), 130.32 (Cq 1), 131.09 (CH 2), 131.70 (Cq 3a), 136.49 (Cq 9a), 144.96 (Cq 4), 146.04 (Cq Phenyl), 146.43 (Cq Phenyl), 154.96 (Cq Phenyl), 172.41 (C=O). MS (ESI +, QTof, *m/z*): 578.3 [M + H]^+^. HRMS calculated for C_31_H_40_N_5_O_6_ 578.2979, found 578.2980.

*Tert-butyl 5-(((benzyloxy)carbonyl)amino)-2-((1-(3,4-dihydroxyphenyl)imidazo[1,2-a]-quinoxalin-4-yl)amino)pentanoate* (**9f**): Following the same procedure used for the synthesis of **9a** (see 4.1.4.8.), to a mixture of **7f** (0.260 g, 0.46 mmol) in DME/H_2_O (2/1, 15 mL) were added compound **12** (0.211 g, 1.37 mmol), tetrakis- (triphenylphosphine) palladium (0.026 g, 0.023 mmol) and sodium carbonate (0.097 g, 0.91 mmol) in a microwave-adapted vial. The product was purified by flash chromatography eluted with cyclohexane/ethyl acetate 80/20 to 50/50. The compound is obtained as a white solid (26% yield). C_33_H_35_N_5_O_6_. MW: 597.66 g/mol. ^1^H-NMR *δ* (ppm, DMSO-*d*_6_): ^1^H-NMR *δ* (ppm, 400 MHz, DMSO-*d*_6_): 1.40 (s, 9H, 3 × CH_3_ OtBu), 1.58–1.60 (m, 2H, CH_2_ γ), 1.91–1.95 (m, 2H, CH_2_ β), 3.05–3.08 (m, 2H, CH_2_ δ), 4.55–4.59 (m, 1H, CH α), 5.01 (s, 2H, CH_2_-Phenyl), 6.81 (d, 1H, *J* = 4 Hz, CH Phenyl), 6.89 (s, 1H, CH Phenyl), 6.93 (d, 1H, *J* = 4 Hz, CH Phenyl), 7.01–7.06 (m, 1H, CH 7), 7.27 (d, 1H, *J* = 4 Hz, NH-CH_2_ δ), 7.31–7.34 (m, 5H, CH Phenyl), 7.37 (dd, 1H, *J* = 4 Hz, *J* = 8 Hz, CH 6), 7.45 (s, 1H, CH 2), 7.54 (d, 1H, *J* = 4 Hz, NH-CH α), 7.56–7.60 (m, 1H, CH 8), 7.63 (dd, 1H, J = *J* = 4 Hz, *J* = 8 Hz, CH 9). ^13^C-NMR *δ* (ppm, DMSO-*d*_6_): 26.54 (CH_2_ γ), 28.09 (CH_2_ β), 28.17 (CH_3_ tBu), 40.45 (CH_2_ δ), 54.34 (CH α), 65.60 (CH_2_-Phenyl), 80.92 (Cq tBu), 115.88 (CH 6), 116.48 (CH Phenyl), 117.90 (CH Phenyl), 121.16 (Cq 1), 122.84 (CH 7), 126.06 (Cq 5a), 126.50 (CH 9), 127.20 (CH 8), 128.18 (CH Phenyl), 128.79 (CH Phenyl), 129.16 (CH Phenyl), 131.90 (CH 2), 132.79 (Cq 3a), 137.55 (Cq 9a), 146.04 (Cq 4), 147.12 (Cq Phenyl), 147.47 (Cq Phenyl), 156.59 (Cq Phenyl), 172.23 (C=O). MS (ESI +, QTof, *m/z*): 598.3 [M + H]^+^. HRMS calculated for C_33_H_36_N_5_O_6_ 598.2666, found 598.2670.

*Tert-butyl 2-((1-(3,4-dihydroxyphenyl)imidazo[1,2-a]quinoxalin-4-yl)amino)-3-phenyl-propanoate* (**9g**): Following the same procedure used for the synthesis of **9a** (see 4.1.4.8.), to a mixture of **7g** (0.360 g, 0.77 mmol) in DME/H_2_O (2/1, 15 mL) were added compound **12** (0.356 g, 2.31 mmol), tetrakis- (triphenylphosphine) palladium (0.044 g, 0.038 mmol) and sodium carbonate (0.163 g, 1.54 mmol) in a microwave-adapted vial. The product was purified by flash chromatography eluted with cyclohexane/ethyl acetate 80/20 to 50/50. The compound is obtained as a white solid (16% yield). C_29_H_28_N_4_O_4_. MW: 496.56 g/mol. ^1^H-NMR *δ* (ppm, DMSO-*d*_6_): 1.36 (s, 9H, 3 × CH_3_ OtBu), 3.23–3.27 (m, 1H, CH_2_ β), 3.33–3.38 (m, 1H, CH_2_ β), 4.91–4.93 (m, 1H, CH α), 6.81 (dd, 1H, *J* = 4 Hz, *J* = 8 Hz, CH Phenyl), 6.89 (d, 1H, *J* = 4 Hz, CH Phenyl), 6.92 (d, 1H, *J* = 8 Hz, CH Phenyl), 7.08 (t, 1H, *J* = 8 Hz, CH Phenyl), 7.20 (t, 1H, *J* = 8 Hz, CH Phenyl), 7.29 (t, 2H, *J* = 8 Hz, CH Phenyl), 7.34 (d, 2H, *J* = 8 Hz, CH 6, CH Phenyl), 7.34–7.37 (m, 1H, CH 7), 7.38–7.40 (m, 1H, CH 8), 7.52 (s, 1H, CH 2), 7.60 (d, 1H, *J* = 8 Hz, CH 9), 7.92–7.93 (m, 1H, NH), 9.38 (s, 2H, C-OH Phenyl). ^13^C-NMR *δ* (ppm, DMSO-*d*_6_): 26.98 (CH_3_ tBu), 35.93 (CH_2_ β), 55.04 (CH α), 80.23 (Cq tBu), 114.96 (CH 6), 115.42 (CH Phenyl), 116.75 (CH Phenyl), 119.67 (Cq 1), 120.96 (CH Phenyl), 122.32 (CH 7), 124.69 (Cq 5a), 125.73 (CH Phenyl, CH 9), 125.94 (CH Phenyl), 127.90 (CH Phenyl), 128.71 (CH 8), 130.76 (Cq Phenyl), 131.11 (CH 2), 131.21 (Cq Phenyl), 136.87 (Cq 3a), 138.12 (Cq 9a), 146.17 (Cq 4), 170.13 (C=O). MS (ESI +, QTof, *m/z*): 497.1 [M + H]^+^. HRMS calculated for C_29_H_29_N_4_O_4_ 497.2189, found 497.2198.

*Tert-butyl 3-(4-(Tert-butoxy)phenyl)-2-((1-(3,4-dihydroxyphenyl)imidazo[1,2-a]quinoxalin-4-yl)amino)propanoate* (**9h**): Following the same procedure used for the synthesis of **9a** (see 4.1.4.8.), to a mixture of **7h** (0.260 g, 0.57 mmol) in DME/H_2_O (2/1, 15 mL) were added compound **12** (0.170 g, 1.72 mmol), tetrakis- (triphenylphosphine) palladium (0.028 g, 0.028 mmol) and sodium carbonate (0.101 g, 1.15 mmol) in a microwave-adapted vial. The product was purified by flash chromatography eluted with cyclohexane/ethyl acetate 80/20 to 50/50. The compound is obtained as a white solid (22% yield). C_33_H_36_N_4_O_5_. MW: 568.66 g/mol. ^1^H-NMR *δ* (ppm, DMSO-*d*_6_): 1.24 (s, 9H, 3 × CH_3_ OtBu), 1.33 (s, 9H, 3 × CH_3_ OtBu), 3.12–3.18 (m, 1H, CH_2_ β), 3.26–3.32 (m, 1H, CH_2_ β), 4.80–4.84 (m, 1H, CH α), 6.80 (d, 1H, *J* = 8 Hz, CH Phenyl), 6.88 (s, 1H, CH Phenyl), 6.90 (dd, 2H, *J* = 4 Hz, *J* = 8 Hz, CH Phenyl), 6.92 (d, 1H, *J* = 8 Hz, CH Phenyl), 7.01–7.05 (m, 1H, CH 7), 7.25 (d, 2H, *J* = 8 Hz, CH Phenyl), 7.30–7.32 (m, 1H, CH 8), 7.34 (dd, 1H, *J* = 4 Hz, *J* = 8 Hz, CH 6), 7.45 (s, 1H, CH 2), 7.54 (dd, 1H, *J* = 4 Hz, *J* = 8 Hz, CH 9), 7.57 (d, 1H, *J* = 8 Hz, NH), 9.31 (s, 1H, C-OH Phenyl), 9.40 (s, 1H, C-OH Phenyl). ^13^C-NMR *δ* (ppm, DMSO-*d*_6_): 28.05 (CH_3_ tBu), 28.95 (CH_3_ tBu), 36.64 (CH_2_ β), 55.87 (CH α), 78.12 (Cq tBu), 80.91 (Cq tBu), 115.90 (CH 6), 116.46 (CH Phenyl), 117.87 (CH Phenyl), 121.08 (Cq 1), 122.05 (CH Phenyl), 122.91 (CH 7), 124.00 (2 × CH Phenyl), 126.07 (Cq 5a), 126.49 (CH 8), 127.21 (CH 9), 130.33 (2 × CH Phenyl), 132.26 (CH 2), 132.62 (Cq 3a), 137.46 (Cq 9a), 146.02 (Cq 4), 147.11 (Cq Phenyl), 147.22 (Cq Phenyl), 154.05 (2 × Cq Phenyl), 171.75 (C=O). MS (ESI +, QTof, *m/z*): 569.3 [M + H]^+^. HRMS calculated for C_33_H_37_N_4_O_5_ 569.2764, found 569.2773.

*Tert-butyl 2-((Tert-butoxycarbonyl)amino)-5-((1-(3,4-dihydroxyphenyl)imidazo[1,2-a]-quinoxalin-4-yl)amino)pentanoate* (**9i**): Following the same procedure used for the synthesis of **9a** (see 4.1.4.8.), to a mixture of **7i** (0.460 g, 0.86 mmol) in DME/H_2_O (2/1, 15 mL) were added compound **12** (0.330 g, 2.15 mmol), tetrakis- (triphenylphosphine) palladium (0.050 g, 0.043 mmol) and sodium carbonate (0.182 g, 1.72 mmol) in a microwave-adapted vial. The product was purified by flash chromatography eluted with cyclohexane/ethyl acetate 80/20 to 50/50. The compound is obtained as a white solid (30% yield). C_30_H_37_N_5_O_6_. MW: 563.64 g/mol. ^1^H-NMR *δ* (ppm, DMSO-*d*_6_): 1.33 (s, 9H, CH-COOtBu), 1.39 (s, 9H, NH-COOtBu), 1.66–1.72 (m, 4H, CH_2_ β, CH_2_ γ), 3.56–3.57 (m, 2H, CH_2_ δ), 3.89–3.93 (m, 1H, CH α), 6.79 (dd, 1H, *J* = 4 Hz, *J* = 8 Hz, CH Phenyl), 6.87 (d, 1H, *J* = 4 Hz, CH Phenyl), 6.90 (d, 1H, *J* = 8 Hz, CH Phenyl), 6.95–7.00 (m, 1H, CH 7), 7.17 (d, 1H, *J* = 8 Hz, NH-CH α), 7.27–7.29 (m, 1H, CH 8), 7.31 (dd, 1H, *J* = 4 Hz, *J* = 8 Hz, CH 6), 7.40 (s, 1H, CH 2), 7.56 (dd, 1H, *J* = 4 Hz, *J* = 8 Hz, CH 9), 7.69 (t, 1H, *J* = 4 Hz, NH-CH_2_ δ), 9.35 (s, 2H, C-OH Phenyl). ^13^C-NMR *δ* (ppm, DMSO-*d*_6_): 26.80 (CH_2_ β), 28.04 (CH_3_ tBu), 28.67 (CH_3_ tBu), 30.42 (CH_2_ γ), 39.79 (CH_2_ δ), 54.78 (CH α), 78.46 (Cq tBu), 80.53 (Cq tBu), 115.79 (CH 6), 116.45 (CH Phenyl), 117.86 (CH Phenyl), 121.29 (Cq 1), 122.01 (CH Phenyl), 122.06 (CH 7), 125.76 (Cq 5a), 126.29 (CH 8), 127.03 (CH 9), 131.91 (CH 2), 138.20 (Cq 3a), 146.02 (Cq 4), 147.05 (Cq Phenyl), 148.03 (Cq Phenyl), 156.02 (Cq 9a), 172.34 (C=O). MS (ESI +, QTof, *m/z*): 564.3 [M + H]^+^. HRMS calculated for C_30_H_38_N_5_O_6_ 564.2822, found 564.2827.

#### 3.1.5. Cleavage of the Protective Groups

*2-((1-(3,4-Dimethoxyphenyl)imidazo[1,2-a]quinoxalin-4-yl)amino)propanoic acid* (**10b**): To a cooled (0 °C) solution of **8b** (0.170 g, 0.36 mmol) in anhydrous CH_2_Cl_2_ (20 mL) was added boron tribromide (2.1 mL, 2.1 mmol). The resulting solution was allowed to warm to room temperature and stirred until complete consumption of the starting material (1–3 h, monitored by TLC). The solution was neutralized by addition of saturated aqueous sodium bicarbonate (20 mL). The crude mixture was extracted with CH_2_Cl_2_ (3 × 20 mL). The organic phase was dried on sodium sulfate, filtered and concentrated under reduced pressure. The product was purified by flash chromatography eluted with cyclohexane/ethyl acetate 90/20 to 40/50. The compound is obtained as a white solid (71% yield). C_21_H_20_N_4_O_4_. MW: 392.41 g/mol. ^1^H-NMR *δ* (ppm, DMSO-*d*_6_): 1.24 (s, 1H, COOH), 1.53 (d, 3H, *J* = 8 Hz, CH_3_ β), 3.75 (s, 3H, OCH_3_ Phenyl), 3.87 (s, 3H, OCH_3_ Phenyl), 4.61–4.63 (m, 1H, CH α), 7.00–7.04 (m, 1H, CH 7), 7.11 (d, 1H, *J* = 4 Hz, CH Phenyl), 7.13 (s, 1H, CH Phenyl), 7.17 (d, 1H, *J* = 4 Hz, CH Phenyl), 7.31 (dd, 1H, *J* = 4 Hz, *J* = 8 Hz, CH 6), 7.33–7.35 (m, 1H, CH 8), 7.50 (s, 1H, CH 2), 7.52 (d, 1H, *J* = 4 Hz, NH), 7.60 (dd, 1H, *J* = 4 Hz, *J* = 8 Hz, CH 9). ^13^C-NMR *δ* (ppm, DMSO-*d*_6_): 18.71 (CH_3_ β), 50.30 (CH α), 56.07 (OCH_3_ Phenyl), 56.16 (OCH_3_ Phenyl), 112.31 (CH Phenyl), 114.22 (CH Phenyl), 115.92 (CH 6), 122.54 (CH 7), 122.70 (Cq 1), 123.28 (CH Phenyl), 126.54 (CH 8), 125.90 (Cq 5a), 127.21 (CH 9), 130.95 (Cq 3a), 132.63 (CH 2), 137.99 (Cq 9a), 147.05 (Cq 4), 149.26 (Cq Phenyl), 150.132 (Cq Phenyl), 172.96 (C=O). MS (ESI +, QTof, m/z): 393.0 [M + H]^+^. HRMS calculated for C_21_H_21_N_4_O_4_ 393.1563, found 393.1558.

*2-((1-(3,4-Dimethoxyphenyl)imidazo[1,2-a]quinoxalin-4-yl)amino)-3-methylbutanoic acid* (**10c**): Following the same procedure used for the synthesis of **10b**, to a cooled solution of **8c** (0.290 g, 0.61 mmol) in anhydrous CH_2_Cl_2_ (20 mL) was added boron tribromide (3.6 mL, 3.6 mmol). The product was purified by flash chromatography eluted with cyclohexane/ethyl acetate 90/20 to 40/50. The compound is obtained as a white solid (80% yield). C_23_H_24_N_4_O_4_. MW: 420.46 g/mol. ^1^H-NMR *δ* (ppm, DMSO-*d*_6_): 0.99 (d, 3H, *J* = 8 Hz, CH_3_ γ), 1.01 (d, 3H, *J* = 8 Hz, CH_3_ γ’), 1.23 (s, 1H, COOH), 2.36–2.41 (m, 1H, CH β), 3.74 (s, 3H, OCH_3_ Phenyl), 3.87 (s, 3H, OCH_3_ Phenyl), 4.55 (t, 1H, *J* = 16 Hz, CH α), 6.97–7.01 (m, 1H, CH 7), 7.10 (dd, 1H, *J* = 4 Hz, *J* = 8 Hz, CH Phenyl), 7.14 (s, 1H, CH Phenyl), 7.16 (dd, 1H, *J* = 4 Hz, *J* = 8 Hz, CH Phenyl), 7.19 (d, 1H, NH-CH α), 7.28 (d, 1H, *J* = 4 Hz, CH 6), 7.29–7.32 (m, 1H, CH 8), 7.48 (s, 1H, CH 2), 7.54 (dd, 1H, *J* = 4 Hz, *J* = 8 Hz, CH 9). ^13^C-NMR *δ* (ppm, DMSO-*d*_6_): 19.02 (CH_3_ γ’), 19.96 (CH_3_ γ), 31.19 (CH β), 56.06 (OCH_3_ Phenyl), 56.16 (OCH_3_ Phenyl), 59.54 (CH α), 112.31 (CH Phenyl), 114.26 (CH Phenyl), 115.87 (CH 6), 122.35 (CH 7), 122.75 (Cq 1), 123.29 (CH Phenyl), 125.89 (Cq 5a), 126.49 (CH 8), 127.18 (CH 9), 130.95 (Cq Phenyl), 132.44 (CH 2), 133.40 (Cq 3a), 138.10 (Cq 9a), 147.60 (Cq 4), 149.25 (Cq Phenyl), 150.12 (Cq Phenyl), 170.00 (C=O). MS (ESI +, QTof, *m/z*): 420.9 [M + H]^+^. HRMS calculated for C_23_H_25_N_4_O_4_ 421.1876, found 421.1873.

*2-((1-(3,4-Dimethoxyphenyl)imidazo[1,2-a]quinoxalin-4-yl)amino)-4-methylpentanoic acid* (**10d**): Following the same procedure used for the synthesis of **10b**, to a cooled solution of **8d** (0.210 g, 0.43 mmol) in anhydrous CH_2_Cl_2_ (20 mL) was added boron tribromide (2.5 mL, 2.53.6 mmol). The product was purified by flash chromatography eluted with cyclohexane/ethyl acetate 90/20 to 40/50. The compound is obtained as a white solid (76% yield). C_24_H_26_N_4_O_4_. MW: 434.49 g/mol. ^1^H-NMR *δ* (ppm, DMSO-*d*_6_): 0.95 (d, 3H, *J* = 8 Hz, CH_3_ δ), 0.98 (d, 3H, *J* = 8 Hz, CH_3_ δ’), 1.76–1.80 (m, 3H, CH_2_ β, CH γ), 3.74 (s, 3H, OCH_3_ Phenyl), 3.87 (s, 3H, OCH_3_ Phenyl), 4.66 (t, 1H, *J* = 4 Hz, CH α), 6.98–7.02 (m, 1H, CH 7), 7.11 (d, 1H, *J* = 4 Hz, CH Phenyl), 7.13 (s, 1H, CH Phenyl), 7.17 (d, 1H, *J* = 4 Hz, CH Phenyl), 7.29 (dd, 1H, *J* = 4 Hz, *J* = 8 Hz, CH 6), 7.31–7.33 (m, 1H, CH 8), 7.44 (d, 1H, *J* = 4 Hz, NH), 7.49 (s, 1H, CH 2), 7.56 (dd, 1H, *J* = 4 Hz, *J* = 8 Hz, CH 9). ^13^C-NMR *δ* (ppm, DMSO-*d*_6_): 22.68 (CH_3_ δ), 23.69 (CH_3_ δ’), 25.24 (CH γ), 40.42 (CH_2_ β), 53.00 (CH α), 56.05 (OCH_3_ Phenyl), 56.15 (OCH_3_ Phenyl), 112.30 (CH Phenyl), 114.24 (CH Phenyl), 115.88 (CH 6), 122.29 (CH 7), 122.77 (Cq 1), 123.27 (CH Phenyl), 125.86 (Cq 5a), 126.49 (CH 8), 127.13 (CH 9), 130.90 (Cq 3a), 132.42 (CH 2), 138.12 (Cq 9a), 147.44 (Cq 4), 149.25 (Cq Phenyl), 150.10 (Cq Phenyl), 172.31 (C=O). MS (ESI +, QTof, *m/z*): 434.9 [M + H]^+^. HRMS calculated for C_24_H_27_N_4_O_4_ 435.2032, found 435.2032.

*2-((1-(3,4-Dihydroxyphenyl)imidazo[1,2-a]quinoxalin-4-yl)amino)acetic acid* (**11a**): To a cooled (0 °C) solution of **9a** (0.055, 0.14 mmol) in anhydrous CH_2_Cl_2_ (10 mL) was added TFA (5 mL). The resulting solution was allowed to warm to room temperature and stirred until complete consumption of the starting material (1–2 h, monitored by TLC). The solvent was removed under reduced pressure. The product was purified by flash chromatography eluted with cyclohexane/ethyl acetate 90/20 to 40/50. The compound is obtained as a white solid (92% yield). C_18_H_14_N_4_O_4_. MW: 350.33 g/mol. ^1^H-NMR *δ* (ppm, DMSO-*d*_6_): 1.23 (s, 1H, COOH), 4.24 (d, 2H, *J* = 8 Hz, CH_2_ α), 6.82 (dd, 1H, *J* = 4 Hz, *J* = 8 Hz, CH Phenyl), 6.90 (dd, 1H, *J* = 4 Hz, *J* = 8 Hz, CH Phenyl), 6.93 (s, 1H, CH Phenyl), 7.03–7.07 (m, 1H, CH 7), 7.31–7.34 (m, 1H, CH 8), 7.35 (dd, 1H, *J* = 4 Hz, *J* = 8 Hz, CH 6), 7.48 (s, 1H, CH 2), 7.58 (dd, 1H, *J* = 4 Hz, *J* = 8 Hz, CH 9), 8.02–8.05 (m, 1H, NH), 9.39–9.43 (m, 2H, C-OH Phenyl). ^13^C-NMR *δ* (ppm, DMSO-*d*_6_): 42.37 (CH_2_ α), 115.97 (CH 6), 116.49 (CH Phenyl), 117.86 (CH Phenyl), 120.69 (Cq 1), 122.06 (CH Phenyl), 122.96 (CH 7), 126.13 (Cq 5a), 126.51 (CH 8), 126.62 (CH 9), 132.36 (CH 2), 146.01 (Cq 3a), 147.17 (Cq 9a), 147.56 (Cq 4), 152.74 (Cq Phenyl), 172.09 (C=O). MS (ESI +, QTof, *m/z*): 351.2 [M + H]^+^. HRMS calculated for C_18_H_15_N_4_O_4_ 351.1093, found 351.1093.

*2-((1-(3,4-dihydroxyphenyl)imidazo[1,2-a]quinoxalin-4-yl)amino)propanoic acid* (**11b**): Following the same procedure for the synthesis of **11a**, to a cooled (0 °C) solution of **9b** (0.040, 0.09 mmol) in anhydrous CH_2_Cl_2_ (10 mL) was added TFA (5 mL). The product was purified by flash chromatography eluted with cyclohexane/ethyl acetate 90/20 to 40/50. The compound is obtained as a white solid (88% yield). C_19_H_16_N_4_O_4_. MW: 364.35 g/mol. ^1^H-NMR *δ* (ppm, DMSO-*d*_6_): 1.23 (s, 1H, COOH), 1.56 (d, 3H, *J* = 8 Hz, CH_3_ β), 4.82–4.84 (m, 1H, CH α), 6.82 (d, 1H, *J* = 4 Hz, CH Phenyl), 6.90 (d, 1H, *J* = 4 Hz, CH Phenyl), 6.93 (s, 1H, CH Phenyl), 7.05–7.08 (m, 1H, CH 7), 7.33 (dd, 1H, *J* = 4 Hz, *J* = 8 Hz, CH 6), 7.35–7.37 (m, 1H, CH 8), 7.50 (s, 1H, CH 2), 7.59 (dd, 1H, *J* = 4 Hz, *J* = 8 Hz, CH 9), 7.95 (d, 1H, *J* = 4 Hz, NH), 9.37 (s, 2H, C-OH Phenyl). ^13^C-NMR *δ* (ppm, DMSO-*d*_6_): 17.79 (CH_3_ β), 49.27 (CH α), 116.01 (CH 6), 116.51 (CH Phenyl), 117.87 (CH Phenyl), 120.42 (Cq 1), 120.95 (Cq 5a), 122.06 (CH Phenyl), 123.17 (CH 7), 125.80 (CH 9), 126.69 (CH 8), 131.82 (Cq 3a), 132.42 (CH 2), 132.64 (Cq 9a), 137.42 (Cq 4), 146.06 (Cq Phenyl), 146.95 (Cq Phenyl), 147.22 (Cq Phenyl), 174.64 (C=O). MS (ESI +, QTof, *m/z*): 365.1 [M + H]^+^. HRMS calculated for C_19_H_17_N_4_O_4_ 365.1250, found 365.1240.

*2-((1-(3,4-Dihydroxyphenyl)imidazo[1,2-a]quinoxalin-4-yl)amino)-3-methylbutanoic acid* (**11c**): Following the same procedure for the synthesis of **11a**, to a cooled (0 °C) solution of **9c** (0.035, 0.08 mmol) in anhydrous CH_2_Cl_2_ (10 mL) was added TFA (5 mL). The product was purified by flash chromatography eluted with cyclohexane/ethyl acetate 90/20 to 40/50. The compound is obtained as a white solid (91% yield). C_21_H_20_N_4_O_4_. MW: 392.41 g/mol. ^1^H-NMR *δ* (ppm, DMSO-*d*_6_): 1.04 (d, 3H, *J* = 8 Hz, CH_3_ γ), 1.07 (d, 3H, *J* = 8 Hz, CH_3_ γ’), 1.18 (s, 1H, COOH), 2.36–2.41 (m, 1H, CH β), 4.74–4.76 (m, 1H, CH α), 6.82 (dd, 1H, *J* = 4 Hz, *J* = 8 Hz, CH Phenyl), 6.90 (d, 1H, *J* = 4 Hz, *J* = 8 Hz, CH Phenyl), 6.94 (d, 1H, *J* = 4 Hz, *J* = 8 Hz, CH Phenyl), 7.06–7.10 (m, 1H, CH 7), 7.17 (d, 1H, *J* = 4 Hz, NH), 7.33–7.36 (m, 1H, CH 8), 7.38 (dd, 1H, *J* = 4 Hz, *J* = 8 Hz, CH 6), 7.51 (s, 1H, CH 2), 7.60 (dd, 1H, *J* = 4 Hz, *J* = 8 Hz, CH 9), 9.40 (s, 2H, C-OH Phenyl). ^13^C-NMR *δ* (ppm, DMSO-*d*_6_): 18.86 (CH_3_ γ’), 19.63 (CH_3_ γ), 30.50 (CH β), 58.70 (CH α), 115.98 (CH 6), 116.49 (CH Phenyl), 117.86 (CH Phenyl), 120.83 (Cq 1), 122.07 (CH Phenyl), 123.29 (CH 7), 126.05 (Cq 5a), 126.72 (CH 8), 126.94 (CH 9), 132.12 (CH 2), 132.52 (Cq Phenyl), 132.82 (Cq 3a), 137.42 (Cq 9a), 146.06 (Cq 4), 147.23 (Cq Phenyl), 147.47 (Cq Phenyl), 171.62 (C=O). MS (ESI +, QTof, *m/z*): 393.2 [M + H]^+^. HRMS calculated for C_21_H_21_N_4_O_4_ 393.1563, found 393.1561.

*2-((1-(3,4-Dihydroxyphenyl)imidazo[1,2-a]quinoxalin-4-yl)amino)-4-methylpentanoic acid* (**11d**): Following the same procedure for the synthesis of **11a**, to a cooled (0 °C) solution of **9d** (0.050, 0.11 mmol) in anhydrous CH_2_Cl_2_ (10 mL) was added TFA (5 mL). The product was purified by flash chromatography eluted with cyclohexane/ethyl acetate 90/20 to 40/50. The compound is obtained as a white solid (78% yield). C_22_H_22_N_4_O_4_. MW: 406.43 g/mol. ^1^H-NMR *δ* (ppm, DMSO-*d*_6_): 0.93 (d, 3H, *J* = 8 Hz, CH_3_ δ), 0.98 (d, 3H, *J* = 8 Hz, CH_3_ δ’), 1.42 (s, 1H, COOH), 1.69–1.72 (m, 1H, CH_2_ β), 1.76–1.79 (m, 1H, CH γ), 1.99–2.02 (m, 1H, CH_2_ β), 4.85 (t, 1H, *J* = 4 Hz, CH α), 6.82 (d, 1H, *J* = 4 Hz, CH Phenyl), 6.90 (s, 1H, CH Phenyl), 6.93 (d, 1H, *J* = 4 Hz, CH Phenyl), 7.05–7.08 (m, 1H, CH 7), 7.32–7.35 (m, 1H, CH 8), 7.36 (dd, 1H, *J* = 4 Hz, *J* = 8 Hz, CH 6), 7.50 (s, 1H, CH 2), 7.59 (dd, 1H, *J* = 4 Hz, *J* = 8 Hz, CH 9), 7.90 (d, 1H, *J* = 4 Hz, NH), 9.38 (s, 2H, C-OH Phenyl). ^13^C-NMR *δ* (ppm, DMSO-*d*_6_): 20.74 (CH_3_ δ), 22.28 (CH_3_ δ’), 24.07 (CH γ), 39.08 (CH_2_ β), 50.92 (CH α), 114.92 (CH 6), 115.42 (CH Phenyl), 116.77 (CH Phenyl), 119.82 (Cq 1), 120.98 (CH Phenyl), 122.10 (CH 7), 124.67 (Cq 5a), 125.64 (CH 8), 125.72 (CH 9), 130.79 (Cq 3a), 131.28 (CH 2), 144.99 (Cq 9a), 146.14 (Cq 4), 146.28 (Cq Phenyl), 157.42 (Cq Phenyl), 173.51 (C=O). MS (ESI +, QTof, *m/z*): 407.1 [M + H]^+^. HRMS calculated for C_22_H_23_N_4_O_4_ 407.1719, found 407.1712.

*6-Amino-2-((1-(3,4-dihydroxyphenyl)imidazo[1,2-a]quinoxalin-4-yl)amino)hexanoic acid* (**11e**): Following the same procedure for the synthesis of **11a**. To a cooled (0 °C) solution of **9e** (0.035, 0.06 mmol) in anhydrous CH_2_Cl_2_ (10 mL) was added TFA (5 mL). The product was purified by flash chromatography eluted with cyclohexane/ethyl acetate 90/20 to 40/50. The compound is obtained as a white solid (84% yield). C_22_H_23_N_5_O_4_. MW: 421.45 g/mol. ^1^H-NMR *δ* (ppm, DMSO-*d*_6_): 1.23 (s, 1H, COOH), 1.47–1.51 (m, 2H, CH_2_ γ), 1.58–1.64 (m, 2H, CH_2_ δ), 1.98–2.04 (m, 2H, CH_2_ β), 2.79–2.83 (m, 2H, CH_2_ ε), 4.76–4.78 (m, 1H, CH α), 6.81 (d, 1H, *J* = 4 Hz, CH Phenyl), 6.90 (s, 1H, CH Phenyl), 6.93 (d, 1H, *J* = 4 Hz, CH Phenyl), 7.03–7.07 (m, 1H, CH 7), 7.31–7.35 (m, 1H, CH 8), 7.37 (dd, 1H, *J* = 4 Hz, *J* = 8 Hz, CH 6), 7.47 (s, 1H, CH 2), 7.58 (dd, 1H, *J* = 4 Hz, *J* = 8 Hz, CH 9), 7.64 (d, 1H, *J* = 4 Hz, NH-CH α), 7.68–7.70 (m, 2H, NH_2_), 9.40–9.46 (m, 2H, C-OH Phenyl). ^13^C-NMR *δ* (ppm, DMSO-*d*_6_): 22.04 (CH_2_ γ), 26.08 (CH_2_ δ), 29.65 (CH_2_ β), 38.04 (CH_2_ ε), 52.17 (CH α), 114.84 (CH 6), 115.40 (CH Phenyl), 116.79 (CH Phenyl), 120.96 (CH Phenyl), 121.87 (CH 7), 124.88 (Cq 5a), 125.49 (CH 8), 126.01 (CH 9), 130.49 (Cq 1), 131.14 (CH 2), 131.69 (Cq 3a), 136.24 (Cq 9a), 145.00 (Cq 4), 146.10 (Cq Phenyl), 146.51 (Cq Phenyl), 157.59 (Cq Phenyl), 173.29 (C=O). MS (ESI +, QTof, *m/z*): 422.1 [M + H]^+^. HRMS calculated for C_22_H_24_N_5_O_4_ 422.1828, found 422.1834.

*5-Amino-2-((1-(3,4-dihydroxyphenyl)imidazo[1,2-a]quinoxalin-4-yl)amino)pentanoic acid* (**11f**): Following the same procedure for the synthesis of **11a**, to a cooled (0 °C) solution of **9f** (0.055, 0.09 mmol) in anhydrous CH_2_Cl_2_ (10 mL) was added TFA (5 mL). The product was purified by flash chromatography eluted with cyclohexane/ethyl acetate 90/20 to 40/50. The compound is obtained as a white solid (10% yield). C_21_H_21_N_5_O_4_. MW: 407.42 g/mol. ^1^H-NMR *δ* (ppm, DMSO-*d*_6_): ^1^H-NMR *δ* (ppm, 400 MHz, DMSO-*d*_6_): 1.70–1.74 (m, 2H, CH_2_ γ), 1.89–1.93 (m, 1H, CH_2_ β), 2.07–2.11 (m, 1H, CH_2_ β), 2.66–2.70 (m, 1H, CH_2_ δ), 2.78–2.82 (m, 1H, CH_2_ δ), 4.33–4.34 (m, 1H, CH α), 6.69 (d, 1H, *J* = 8 Hz, CH Phenyl), 6.80 (d, 1H, *J* = 8 Hz, CH Phenyl), 6.89 (d, 1H, *J* = 4 Hz, CH Phenyl), 6.97–7.01 (m, 1H, CH 7), 7.29-.33 (m, 1H, CH 8), 7.36 (dd, 1H, *J* = 4 Hz, *J* = 8 Hz, CH 6), 7.40 (s, 1H, *J* = 4 Hz, CH 2), 7.46 (d, 1H, *J* = 4 Hz, NH-CH α), 7.57 (dd, 1H, *J* = 4 Hz, *J* = 8 Hz, CH 9), 8.69 (s, 2H, C-OH Phenyl). ^13^C-NMR *δ* (ppm, DMSO-*d*_6_): 23.61 (CH_2_ γ), 29.41 (CH_2_ β), 40.41 (CH_2_ δ), 54.57 (CH α), 115.88 (CH 6), 116.50 (CH Phenyl), 117.95 (CH Phenyl), 121.23 (Cq 1), 122.04 (CH 7), 122.18 (CH Phenyl), 125.85 (Cq 5a), 126.39 (CH 8), 127.17 (CH 9), 132.08 (CH 2), 133.25 (Cq 3a), 138.31 (Cq 9a), 146.07 (Cq 4), 146.84 (Cq Phenyl), 147.12 (Cq Phenyl), 174.19 (C=O). MS (ESI +, QTof, *m/z*): 408.2 [M + H]^+^. HRMS calculated for C_21_H_22_N_5_O_4_ 408.1672, found 408.1668.

*2-((1-(3,4-Dihydroxyphenyl)imidazo[1,2-a]quinoxalin-4-yl)amino)-3-phenylpropanoic acid* (**11g**): Following the same procedure for the synthesis of **11a**, to a cooled (0 °C) solution of **9g** (0.045, 0.09 mmol) in anhydrous CH_2_Cl_2_ (10 mL) was added TFA (5 mL). The product was purified by flash chromatography eluted with cyclohexane/ethyl acetate 90/20 to 40/50. The compound is obtained as a white solid (87% yield). C_25_H_20_N_4_O_4_. MW: 440.45 g/mol. ^1^H-NMR *δ* (ppm, DMSO-*d*_6_): 1.23 (s, 1H, COOH), 3.33–3.39 (m, 2H, CH_2_ β), 5.04–5.07 (m, 1H, CH α), 6.81 (d, 1H, *J* = 8 Hz, CH Phenyl), 6.89 (d, 1H, *J* = 4 Hz, CH Phenyl), 6.91 (d, 1H, *J* = 8 Hz, CH Phenyl), 7.05–7.08 (m, 1H, CH 7), 7.17 (t, 1H, *J* = 8 Hz, CH Phenyl), 7.26 (t, 2H, *J* = 8 Hz, CH Phenyl), 7.31–7.333 (m, 1H, CH 8), 7.34 (dd, 1H, *J* = 4 Hz, *J* = 8 Hz, CH 6), 7.36–7.37 (m, 2H, CH Phenyl), 7.48 (s, 1H, CH 2), 7.61 (dd, 1H, *J* = 4 Hz, *J* = 8 Hz, CH 9), 7.76–7.77 (m, 1H, NH), 9.39 (s, 2H, C-OH Phenyl). ^13^C-NMR *δ* (ppm, DMSO-*d*_6_): 35.61 (CH_2_ β), 5.31 (CH α), 114.91 (CH 6), 115.40 (CH Phenyl), 116.75 (CH Phenyl), 119.75 (Cq 1), 120.96 (CH Phenyl), 122.23 (CH 7), 124.71 (Cq 5a), 125.64 (CH 9), 125.88 (CH 8), 127.67 (CH Phenyl), 128.58 (CH Phenyl), 130.78 (Cq Phenyl), 131.35 (CH 2), 137.26 (Cq 3a), 138.42 (Cq 9a), 144.98 (Cq Phenyl), 146.14 (Cq 4), 157.48 (Cq Phenyl), 157.77 (Cq Phenyl), 172.35 (C=O). MS (ESI +, QTof, *m/z*): 441.2 [M + H]^+^. HRMS calculated for C_25_H_21_N_4_O_4_ 441.1563, found 441.1569.

*2-((1-(3,4-Dihydroxyphenyl)imidazo[1,2-a]quinoxalin-4-yl)amino)-3-(4-hydroxyphenyl)-propanoic acid* (**11h**): Following the same procedure for the synthesis of **11a**, to a cooled (0 °C) solution of **9h** (0.030, 0.06 mmol) in anhydrous CH_2_Cl_2_ (10 mL) was added TFA (5 mL). The product was purified by flash chromatography eluted with cyclohexane/ethyl acetate 90/20 to 40/50. The compound is obtained as a white solid (89% yield). C_25_H_20_N_4_O_5_. MW: 456.45 g/mol. ^1^H-NMR *δ* (ppm, DMSO-*d*_6_): 1.29 (s, 1H, COOH), 3.21–3.23 (m, 2H, CH_2_ β), 4.94–4.98 (m, 1H, CH α), 6.71 (d, 2H, *J* = 8 Hz, CH Phenyl), 6.86 (dd, 1H, *J* = 4 Hz, *J* = 8 Hz, CH Phenyl), 6.94 (d, 1H, CH Phenyl), 6.98 (d, 1H, *J* = 8 Hz, CH Phenyl), 7.04–7.08 (m, 1H, CH 7), 7.12 (d, 2H, *J* = 8 Hz, CH Phenyl), 7.31–7.32 (m, 1H, CH 8), 7.35 (dd, 1H, *J* = 4 Hz, *J* = 8 Hz, CH 6), 7.47 (s, 1H, CH 2), 7.59–7.60 (m, 1H, NH), 7.64 (dd, 1H, *J* = 4 Hz, *J* = 8 Hz, CH 9), 9.32–9.35 (m, 2H, C-OH Phenyl). ^13^C-NMR *δ* (ppm, DMSO-*d*_6_): 36.01 (CH_2_ β), 55.24 (CH α), 115.56 (2 × CH Phenyl), 115.96 (CH 6), 116.47 (CH Phenyl), 117.82 (CH Phenyl), 120.86 (Cq 1), 122.05 (CH Phenyl), 123.19 (CH 7), 125.81 (Cq 5a), 126.68 (CH 9), 126.70 (CH 8), 130.59 (2 × CH Phenyl), 132.35 (Cq 3a), 132.48 (CH 2), 137.32 (Cq 9a), 146.03 (Cq 4), 147.05 (Cq Phenyl), 147.19 (Cq Phenyl), 156.44 (Cq Phenyl), 173.55 (C=O). MS (ESI +, QTof, *m/z*): 457.1 [M + H]^+^. HRMS calculated for C_25_H_21_N_4_O_5_ 457.1512, found 457.1514.

*2-Amino-5-((1-(3,4-dihydroxyphenyl)imidazo[1,2-a]quinoxalin-4-yl)amino)pentanoic acid* (**11i**): Following the same procedure for the synthesis of **11a**, to a cooled (0 °C) solution of **9i** in anhydrous CH_2_Cl_2_ (10 mL) was added TFA (5 mL). The product was purified by flash chromatography eluted with cyclohexane/ethyl acetate 90/20 to 40/50. The compound is obtained as a white solid (79% yield). C_21_H_21_N_5_O_4_. MW: 407.42 g/mol. ^1^H-NMR *δ* (ppm, DMSO-*d*_6_): 1.23 (s, 1H, COOH), 1.85–1.89 (m, 4H, CH_2_ γ, CH_2_ β), 3.64–3.66 (m, 2H, CH_2_ δ), 3.99–4.01 (m, 1H, CH α), 6.80 (dd, 1H, *J* = 4 Hz, *J* = 8 Hz, CH Phenyl), 6.89 (d, 1H, *J* = 4 Hz, CH Phenyl), 6.92 (d, 1H, *J* = 8 Hz, CH Phenyl), 7.07–7.11 (m, 1H, CH 7), 7.35 (dd, 1H, *J* = 4 Hz, *J* = 8 Hz, CH 6), 7.37–7.39 (m, 1H, CH 8), 7.51 (s, 1H, CH 2), 7.55 (dd, 1H, *J* = 4 Hz, *J* = 8 Hz, CH 9), 7.66 (t, 1H, *J* = 4 Hz, NH-CH_2_ δ), 8.24 (s, 2H, NH_2_), 9.40 (s, 2H, C-OH Phenyl). ^13^C-NMR *δ* (ppm, DMSO-*d*_6_): 24.80 (CH_2_ γ), 28.04 (CH_2_ β), 39.99 (CH_2_ δ), 52.30 (CH α), 116.12 (CH 6), 116.51 (CH Phenyl), 117.77 (CH Phenyl), 120.74 (Cq 1), 121.96 (CH Phenyl), 123.27 (CH 7), 125.76 (Cq 5a), 126.83 (CH 8), 129.28 (CH 9), 132.68 (CH 2), 138.42 (Cq 3a), 146.12 (Cq 4), 147.29 (Cq Phenyl), 158.41 (Cq Phenyl), 158.75 (Cq 9a), 171.54 (C=O). MS (ESI +, QTof, *m/z*): 408.2 [M + H]^+^. HRMS calculated for C_21_H_22_N_5_O_4_ 408.1672, found 408.1668.

#### 3.1.6. 3,4-Dihydroxyphenylboronic Acid (**12**)

To a cooled (0 °C) solution of 3,4-dimethoxyphenylboronic acid (0.800 g, 4.39 mmol) in anhydrous CH_2_Cl_2_ (50 mL) was added boron tribromide (10 mL, 10 mmol). The resulting solution was allowed to warm to room temperature and stirred until complete consumption of the starting material (1–2h, monitored by TLC). The solution was neutralized by addition of methanol (50 mL). The crude mixture was concentrated under reduced pressure. The compound was obtained as a white solid (84% yield) and used without purification. C_6_H_7_BO_4_. MW: 153.93 g/mol. ^1^H-NMR *δ* (ppm, DMSO-*d*_6_): 2.08 (s, 2H, B-OH), 6.47 (d, 1H, CH Phenyl), 6.60 (d, 1H, CH Phenyl), 6.71 (d, 1H, CH Phenyl), 8.00 (s, 2H, C-OH Phenyl). ^13^C-NMR *δ* (ppm, DMSO-*d*_6_): 108.10 (CH Phenyl), 116.14 (CH Phenyl), 119.73(CH Phenyl), 145.73 (C-OH). MS (ESI +, QTof, m/z): 153.2 [M−H]^−^. HRMS calculated for C_6_H_6_O_4_B 153.0359, found 153.0358.

### 3.2. Cell Line and Culture Techniques

The melanoma (A375) human cancer cell line is obtained from American Type Culture Collection (Rockville, MD, USA). Cells were cultured in RPMI Gibco medium containing RPMI-1640 (Waltham, MA, USA), 10% heat-inactived (56 °C) foetal bovine serum (FBS) (Polylabo, Paris, France), 2 mM l-glutamine, 100 IU/mL penicillin G sodium, 100 mg/mL streptomycin sulfate, and 0.25 mg/mL amphotericin B. Cells were maintained in a humidified atmosphere of 5% CO_2_ in air at 37 °C.

### 3.3. In Vitro Cytotoxicity Assay

Previously to the experiments, the number of cells by well, the doubling time and the MTT concentration have been optimized. In all the experiments, A375 cells were seeded at a final concentration of 5000 cells/well in 96-well microtiter plates and allowed to attach overnight. After 24 h incubation, the medium (phosphate-buffer saline pH 7.3) was aspirated carefully from the plates using a sterile Pasteur pipette, and cells were exposed (i) to vehicle controls (0.15% DMSO/culture medium (*v*/*v*) and culture medium alone), (ii) to EAPB02303, EAPB02302 and the synthesized compounds at concentrations of 10^−5^–3.2 × 10^−9^ M dissolved in a mixture 0.15% DMSO/culture medium (*v*/*v*). After 96 h of incubation, cell supernatant was removed and 100 μL of a MTT (3-[4.5-dimethylthiazol-2-yl]-2.5-diphenyltetrazolium bromide) solution in fresh medium was added per well (MTT final concentration of 0.5 mg/mL) and incubated for 4 h at 37 °C. This colorimetric assay is based on the ability of live and metabolically unimpaired tumor-cell targets to reduce MTT to a blue formazan product. At the end of the incubation period, the supernatant was carefully aspirated, then, 100 µL of a mixture of isopropyl alcohol and 1 M hydrochloric acid (96/4, *v*/*v*) was added to each well. After 10 min of incubation and vigorous shaking to solubilize formazan crystals, the optical density was measured at 570 nm in a microculture plate reader (Zaragoza, Spain). For each assay, at least three experiments were performed in triplicate. The individual cell line growth curves confirmed that all A375 line in control medium remained in the log phase of cell growth 96 h after plating. Cell survival was expressed as percent of vehicle control. The IC_50_ values defined as the concentrations of drugs which produced 50% cell growth inhibition; 50% reduction of absorbance, were estimated from the sigmoidal dose–response curves.

## 4. Conclusions

The synthesis and study of the amino acid groups grafted on position 4 within the imiqualine series highlight the fact that the nature of the substituent on position 4 is not essential for the biological activity. Indeed, large modifications between EAPB02302, which only has a primary amine, and the new compounds with a complete amino acid residue do not significantly modify the activity, which remains similar to that of our first imiqualine generation. However, these modulations allow one to significantly increase the theoretical water solubility. The presence of dihydroxy groups on the phenyl appears to be necessary for the conservation of the cytotoxic activity on the melanoma cell line tested. These encouraging results obtained on the representative A375 melanoma cell line will prompt us to study further in vivo evaluation on xenografted mice.

## Figures and Tables

**Table 1:** Synthesis of imidazo[1,2-*a*]quinoxaline derivatives grafted with the α-amine of the amino acid as described in Scheme 2: cLogP, theoretical water solubility (mg/mL) at pH 7.4 calculated values and IC_50_ values against A375 (human melanoma cell line).

Amino Acids	─R	─R’	Compounds	ClogP ^a^	Theoretical Water Solubility (mg/mL) at pH 7.4 ^b^	IC_50_ Values ^c^ (nM)
**Gly**	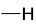	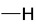	**8a**	5.25	5.88 × 10^−4^	ND ^d^
			**9a**	4.99	1.79 × 10^−3^	1932
			**11a**	2.59	47.18	403
**Ala**	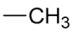	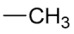	**8b**	5.61	3.80 × 10^−4^	>10,000
			**9b**	5.34	1.15 × 10^−3^	6103
			**10b**	3.21	6.01	5947
			**11b**	2.94	19.92	584
**Val**	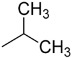	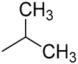	**8c**	6.47	1.35 × 10^−4^	>10,000
			**9c**	6.21	4.07 × 10^−4^	7180
			**10c**	4.07	1.91	>10,000
			**11c**	3.81	6.5	7166
**Leu**	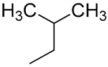	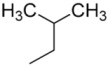	**8d**	6.98	7.43 × 10^−5^	>10,000
			**9d**	6.72	2.23 × 10^−4^	128
			**10d**	4.58	0.89	>10,000
			**11d**	4.32	3.08	838
**Lys**	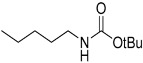	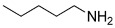	**8e**	7.05	5.70 × 10^−5^	ND ^d^
			**9e**	6.79	1.56 × 10^−4^	4575
			**11e**	2.78	4.34 × 10^−3^	673
**Orn**	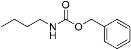	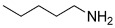	**8f**	7.01	3.43 × 10^−5^	>10,000
			**9f**	6.75	9.07 × 10^−5^	1111
			**11f**	2.57	5.82 × 10^−3^	3404
**Phe**	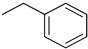	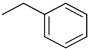	**9g**	6.82	1.06 × 10^−4^	1999
			**11g**	3.71	6.47	951
**Tyr**	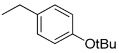	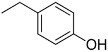	**9h**	7.99	3.31 × 10^−5^	4117
			**11h**	3.05	10.17	2174

^a,b^ cLogP and theoretical water solubility (mg/mL) at pH 7.4 values are calculated using the ACDLabs® software; ^c^ IC_50_ values, concentration of the compound (nM) producing 50% cell growth inhibition after 96 h of drug exposure, as determined by the MTT assay. Each experiment was performed in triplicate, and the results are presented as average values. Coefficients of variation were less than 10%; ^d^ ND: Not determined.

**Table 2:** Synthesis of imidazo[1,2-*a*]quinoxaline derivatives grafted with the side chain amine of ornithine as described in Scheme 3: ClogP, theoretical water solubility (mg/mL) at pH 7.4 calculated values and IC_50_ values against A375 (human melanoma cell line).

Amino acid	Compounds	ClogP ^a^	Theoretical Water Solubility (mg/mL) at pH 7.4 ^b^	IC_50_ Values ^c^ (nM)
**Orn**	**8i**	6.12	1.62 × 10^−4^	>10,000
	**9i**	5.86	4.50 × 10^−4^	>10,000
	**11i**	2.99	3.05 × 10^−3^	5168

^a,b^ ClogP and theoretical water solubility (mg/mL) at pH 7.4 values are calculated using the ACDLabs® software. ^c^ IC_50_ values, concentration of the compound (nM) producing 50% cell growth inhibition after 96 h of drug exposure, as determined by the MTT assay. Each experiment was performed in triplicate, and the results are presented as average values. Coefficients of variation were less than 10%.

**Table 3:** First and second generation imiqualines leads: ClogP, theoretical water solubility (mg/mL) at pH 7.4 calculated values and IC50 values against A375 (human melanoma cell line).

Compounds	ClogP ^a^	Theoretical Water Solubility (mg/mL) at pH 7.4 ^b^	IC_50_ Values ^c^ (nM)
**EAPB0203**	4.6	3.46 × 10^−3^	1570
**EAPB0503**	4.48	2.60 × 10^−3^	200
**EAPB02302**	2.68	4.28 × 10^−2^	60
**EAPB02303**	3.55	1.74 × 10^−2^	10

^a,b^ ClogP and theoretical water solubility (mg/mL) at pH 7.4 values are calculated using the ACDLabs® software. ^c^ IC_50_ values, concentration of the compound (nM) producing 50% cell growth inhibition after 96 h of drug exposure, as determined by the MTT assay. Each experiment was performed in triplicate, and the results are presented as average values. Coefficients of variation were less than 10%.

## Supplementary Materials

The following are available online. NMR ^1^H and ^13^C spectra of all compounds evaluated on A375 melanoma cells.
