# Supplementary material for: Imidazo[1,2-a]quinoxalines Derivatives Grafted with Amino Acids: Synthesis and Evaluation on A375 Melanoma Cells
Source: Molecules. 2018 Nov 15;23(11):2987. doi: 10.3390/molecules23112987 (PMC6278480; doi:10.3390/molecules23112987)
Supplement: Supplementary file 1 [file molecules-23-02987-s001.pdf]

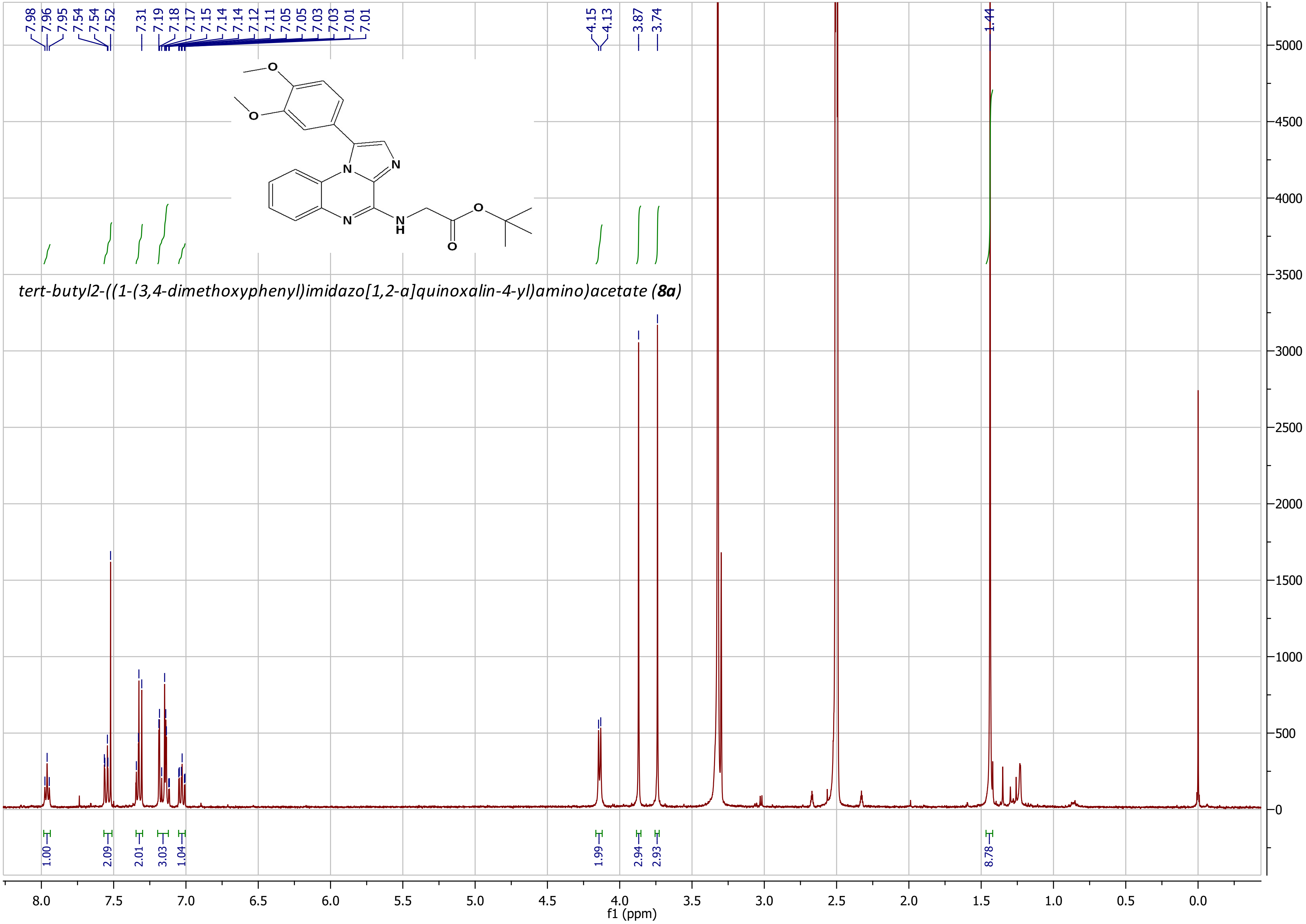
7.98
7.96
7.95
7.54
7.54
7.52
7.31
7.19
7.18
7.17
7.15
7.14
7.14
7.12
7.11
7.05
7.05
7.03
7.03
7.01
7.01
4.15
4.13
3.87
3.74
1.44
tert-butyl2-((1-(3,4-dimethoxyphenyl)imidazo[1,2-a]quinoxalin-4-yl)amino)acetate (8a)
1.00
2.09
2.01
3.03
1.04
1.99
2.94
2.93
8.78
f1 (ppm)

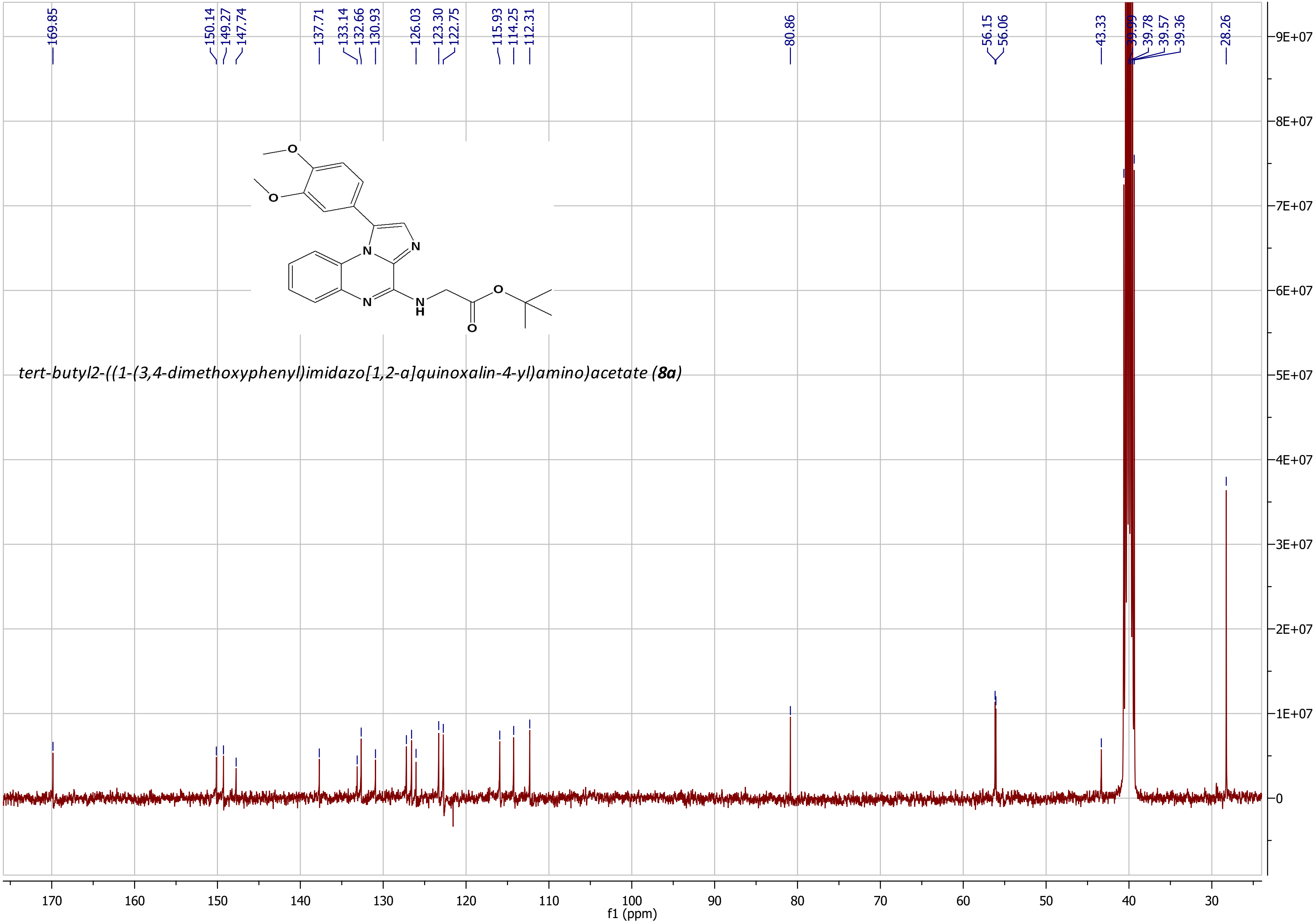

*tert-butyl2-((1-(3,4-dimethoxyphenyl)imidazo[1,2-a]quinoxalin-4-yl)amino)acetate (**8a**)*

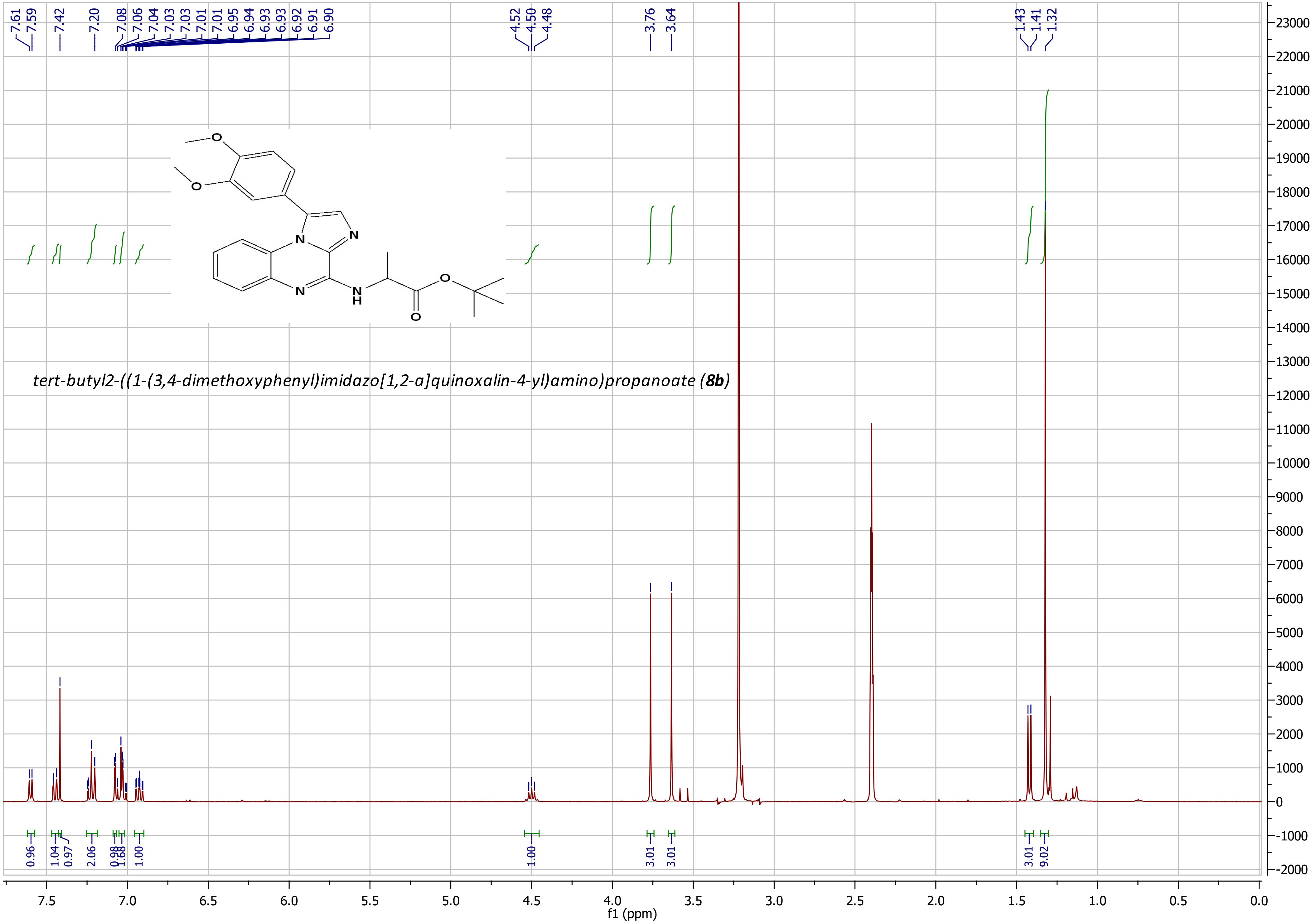
tert-butyl2-((1-(3,4-dimethoxyphenyl)imidazo[1,2-a]quinoxalin-4-yl)amino)propanoate (8b)
f1 (ppm)

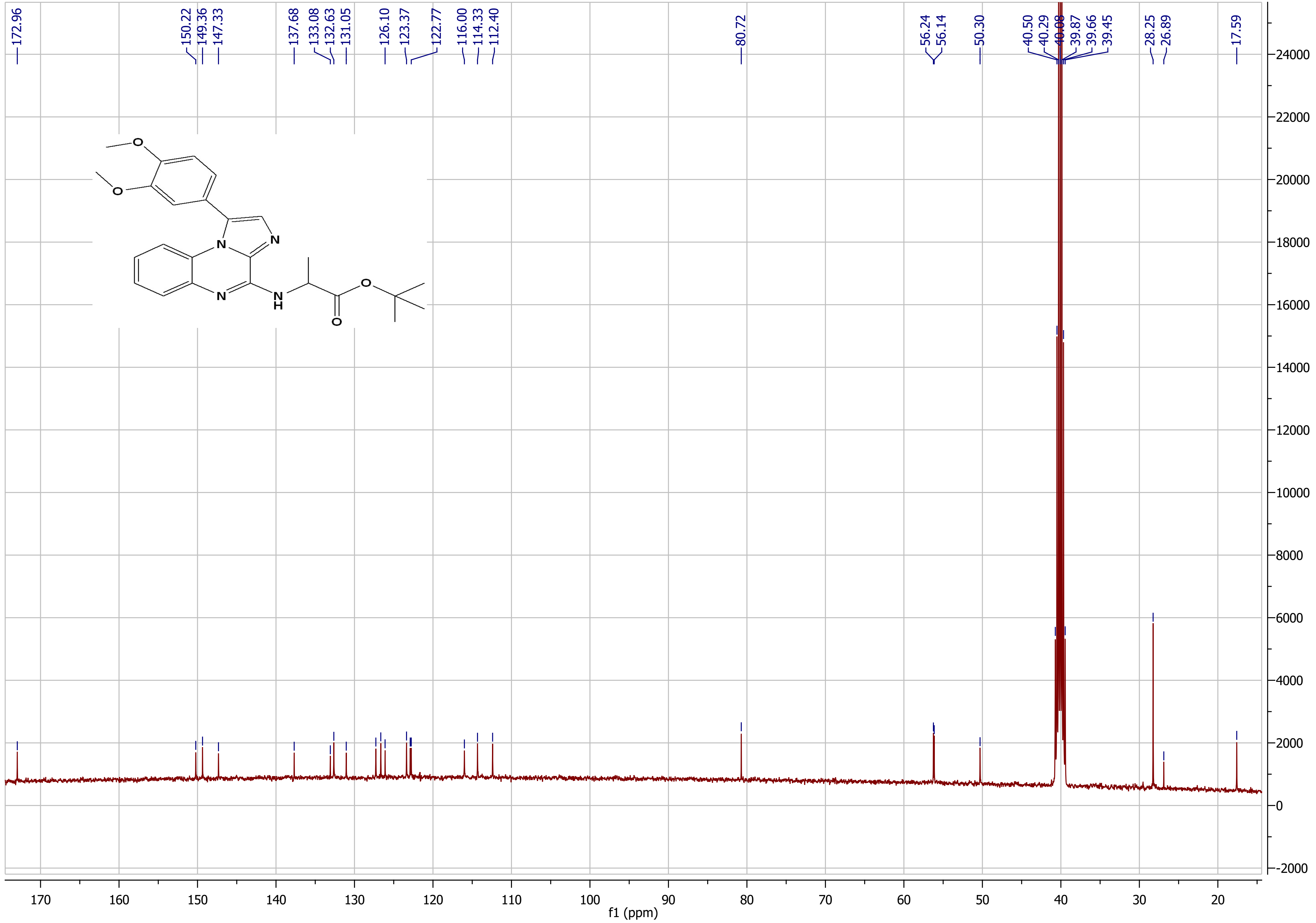
172.96
150.22
149.36
147.33
137.68
133.08
132.63
131.05
126.10
123.37
122.77
116.00
114.33
112.40
80.72
56.24
56.14
50.30
40.50
40.29
40.08
39.87
39.66
39.45
28.25
26.89
17.59
f1 (ppm)

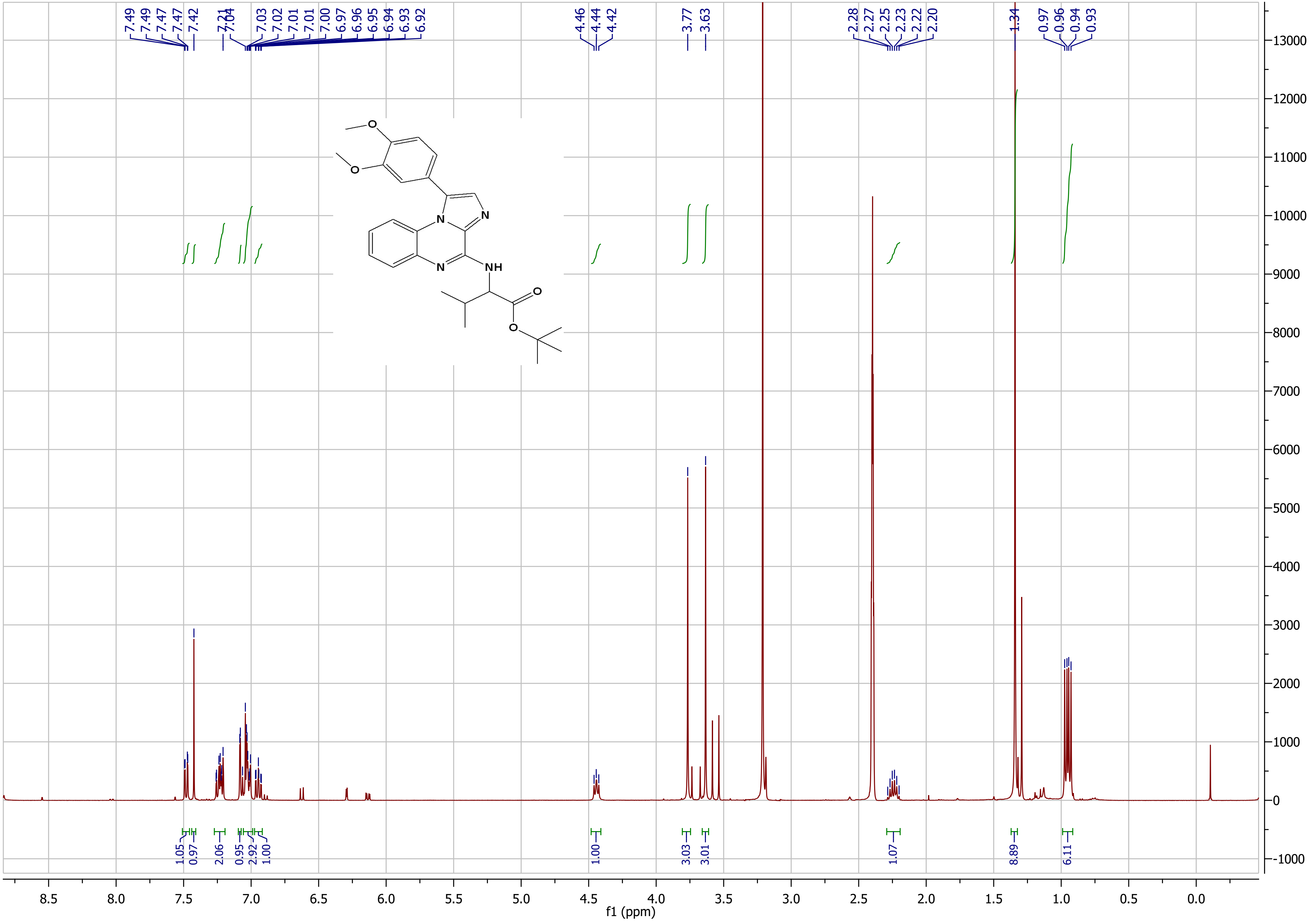
7.49
7.49
7.47
7.47
7.42
7.21
7.04
7.03
7.02
7.01
7.01
7.00
6.97
6.96
6.95
6.94
6.93
6.92
4.46
4.44
4.42
3.77
3.63
2.28
2.27
2.25
2.23
2.22
2.20
1.34
0.97
0.96
0.94
0.93
O
O
N
N
N
NH
O
O
1.05
0.97
2.06
0.95
2.92
1.00
1.00
3.03
3.01
1.07
8.89
6.11
13000
12000
11000
10000
9000
8000
7000
6000
5000
4000
3000
2000
1000
0
-1000
8.5
8.0
7.5
7.0
6.5
6.0
5.5
5.0
4.5
4.0
3.5
3.0
2.5
2.0
1.5
1.0
0.5
0.0
f1 (ppm)

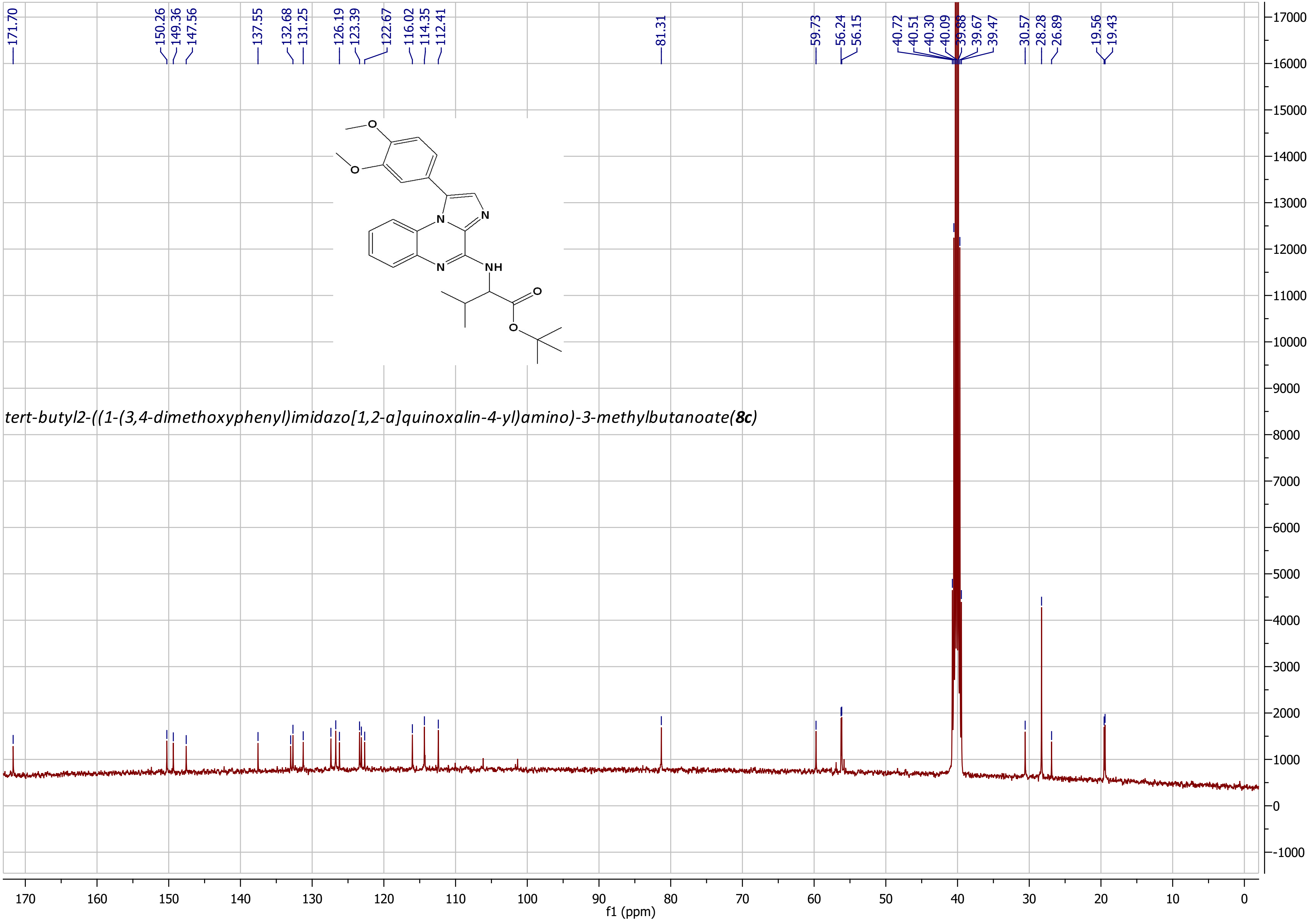

*tert-butyl2-((1-(3,4-dimethoxyphenyl)imidazo[1,2-a]quinoxalin-4-yl)amino)-3-methylbutanoate(**8c**)*

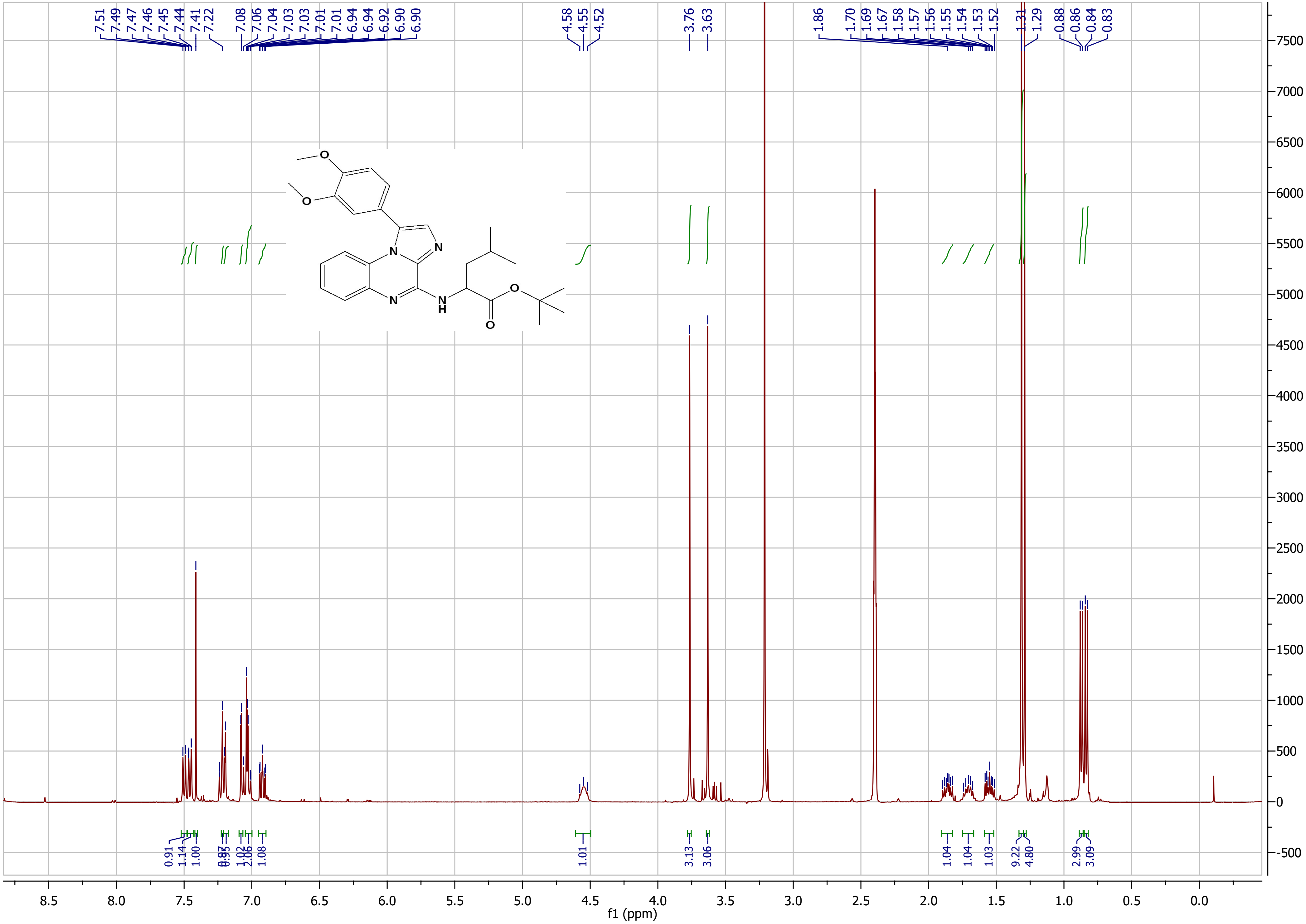

7.51
7.49
7.47
7.46
7.45
7.44
7.41
7.22
7.08
7.06
7.04
7.03
7.03
7.01
7.01
6.94
6.94
6.92
6.90
6.90
4.58
4.55
4.52
3.76
3.63
1.86
1.70
1.69
1.67
1.58
1.57
1.56
1.55
1.54
1.53
1.52
1.31
1.29
0.88
0.86
0.84
0.83
0.91
1.14
1.00
0.97
0.95
1.02
2.06
1.08
1.01
3.13
3.06
1.04
1.04
1.03
9.22
4.80
2.99
3.09
O
O
N
N
N
N
H
O
O
f1 (ppm)

*tert-butyl2-((1-(3,4-dimethoxyphenyl)imidazo[1,2-a]quinoxalin-4-yl)amino)-4-methylpentanoate(**8d**)*

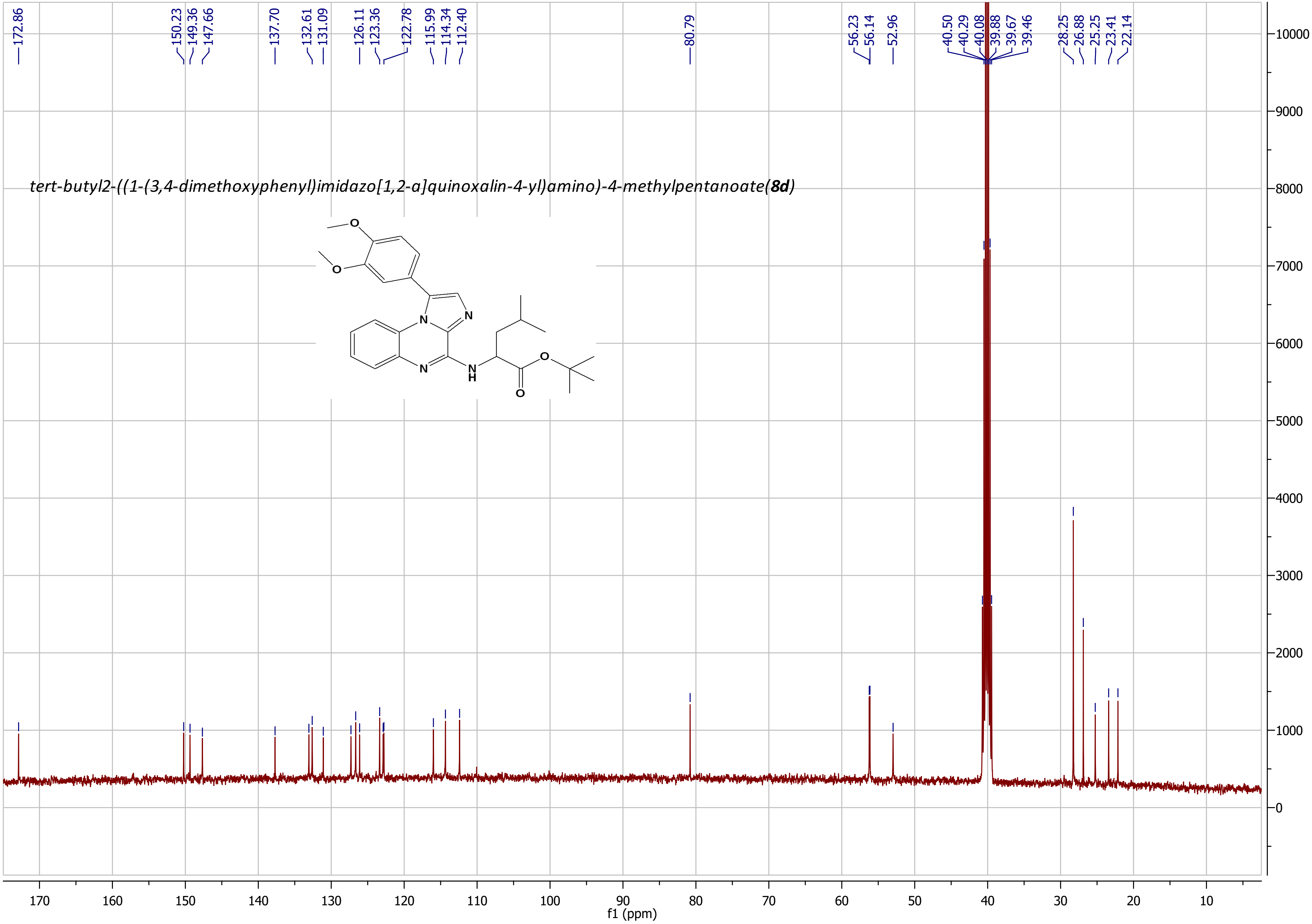

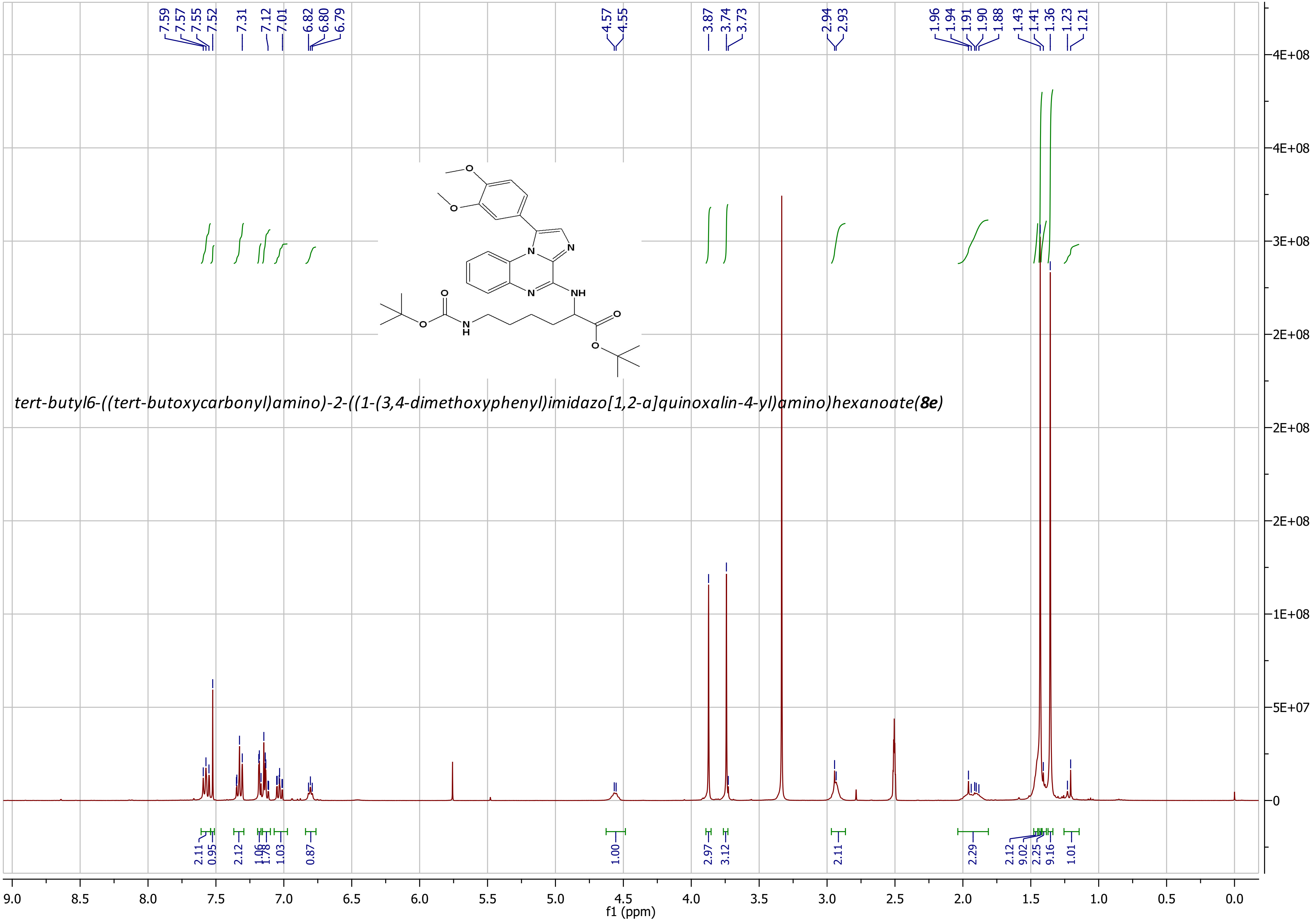
7.59
7.57
7.55
7.52
7.31
7.12
7.01
6.82
6.80
6.79
4.57
4.55
3.87
3.74
3.73
2.94
2.93
1.96
1.94
1.91
1.90
1.88
1.43
1.41
1.36
1.23
1.21
tert-butyl6-((tert-butoxycarbonyl)amino)-2-((1-(3,4-dimethoxyphenyl)imidazo[1,2-a]quinoxalin-4-yl)amino)hexanoate(**8e**)
2.11
0.95
2.12
1.06
1.78
1.03
0.87
1.00
2.97
3.12
2.11
2.29
2.12
9.02
2.25
9.16
1.01
f1 (ppm)

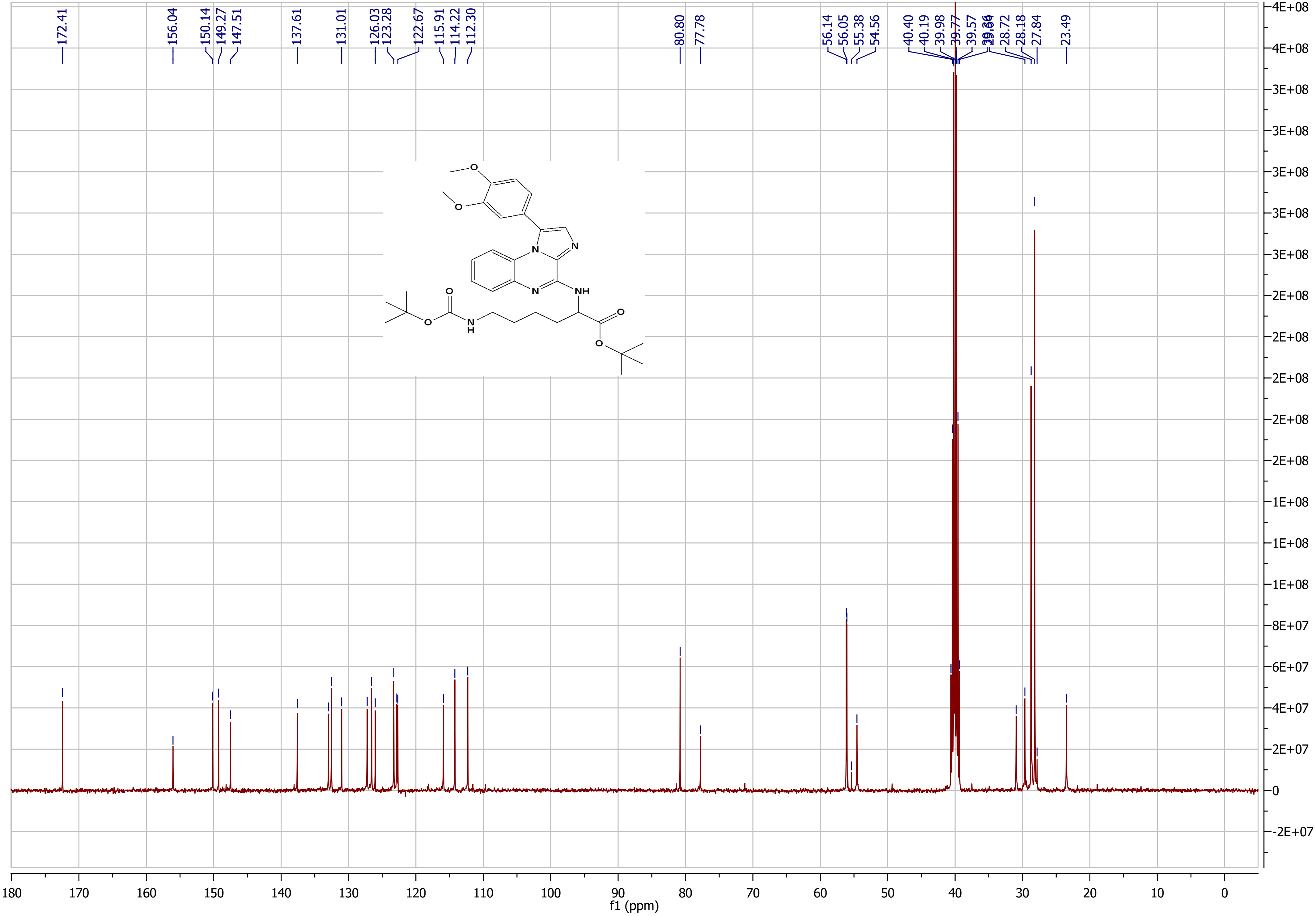
172.41
156.04
150.14
149.27
147.51
137.61
131.01
126.03
123.28
122.67
115.91
114.22
112.30
80.80
77.78
56.14
56.05
55.38
54.56
40.40
40.19
39.98
39.77
39.57
28.72
28.18
27.84
23.49
O
O
N
N
N
NH
O
O
N
H
O
O
4E+08
3E+08
2E+08
1E+08
8E+07
6E+07
4E+07
2E+07
0
-2E+07
180
170
160
150
140
130
120
110
100
90
80
70
60
50
40
30
20
10
0
f1 (ppm)

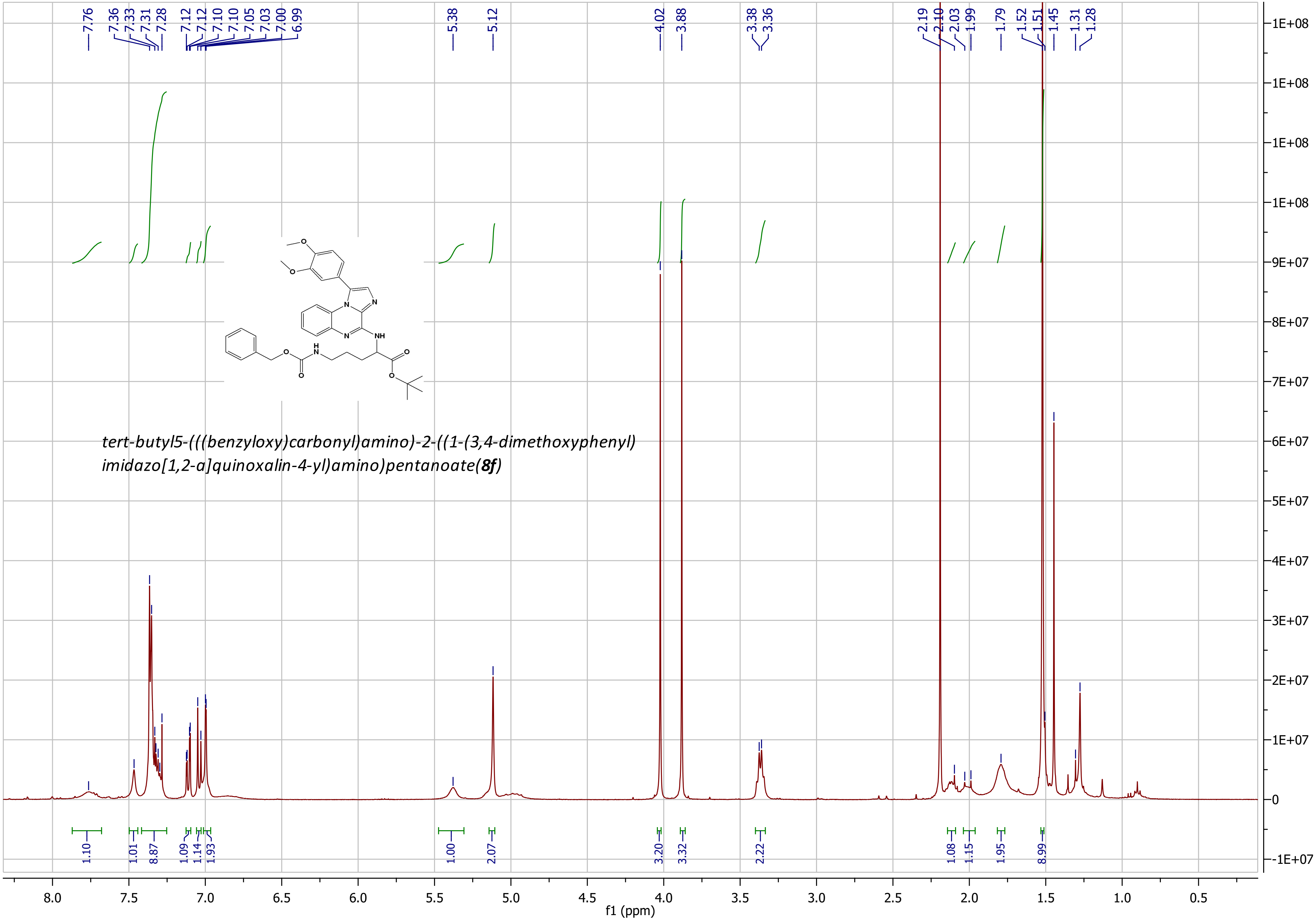
7.76
7.36
7.33
7.31
7.28
7.12
7.12
7.10
7.10
7.05
7.03
7.00
6.99
5.38
5.12
4.02
3.88
3.38
3.36
2.19
2.10
2.03
1.99
1.79
1.52
1.51
1.45
1.31
1.28
O
O
N
N
N
NH
H
N
O
O
O
O
tert-butyl5-(((benzyloxy)carbonyl)amino)-2-((1-(3,4-dimethoxyphenyl)
imidazo[1,2-a]quinoxalin-4-yl)amino)pentanoate(8f)
1.10
1.01
8.87
1.09
1.14
1.93
1.00
2.07
3.20
3.32
2.22
1.08
1.15
1.95
8.99
8.0
7.5
7.0
6.5
6.0
5.5
5.0
4.5
4.0
3.5
3.0
2.5
2.0
1.5
1.0
0.5
f1 (ppm)
1E+08
1E+08
1E+08
1E+08
9E+07
8E+07
7E+07
6E+07
5E+07
4E+07
3E+07
2E+07
1E+07
0
-1E+07

*tert-butyl5-(((benzyloxy)carbonyl)amino)-2-((1-(3,4-dimethoxyphenyl)imidazo[1,2-a]quinoxalin-4-yl)amino)pentanoate(**8f**)*

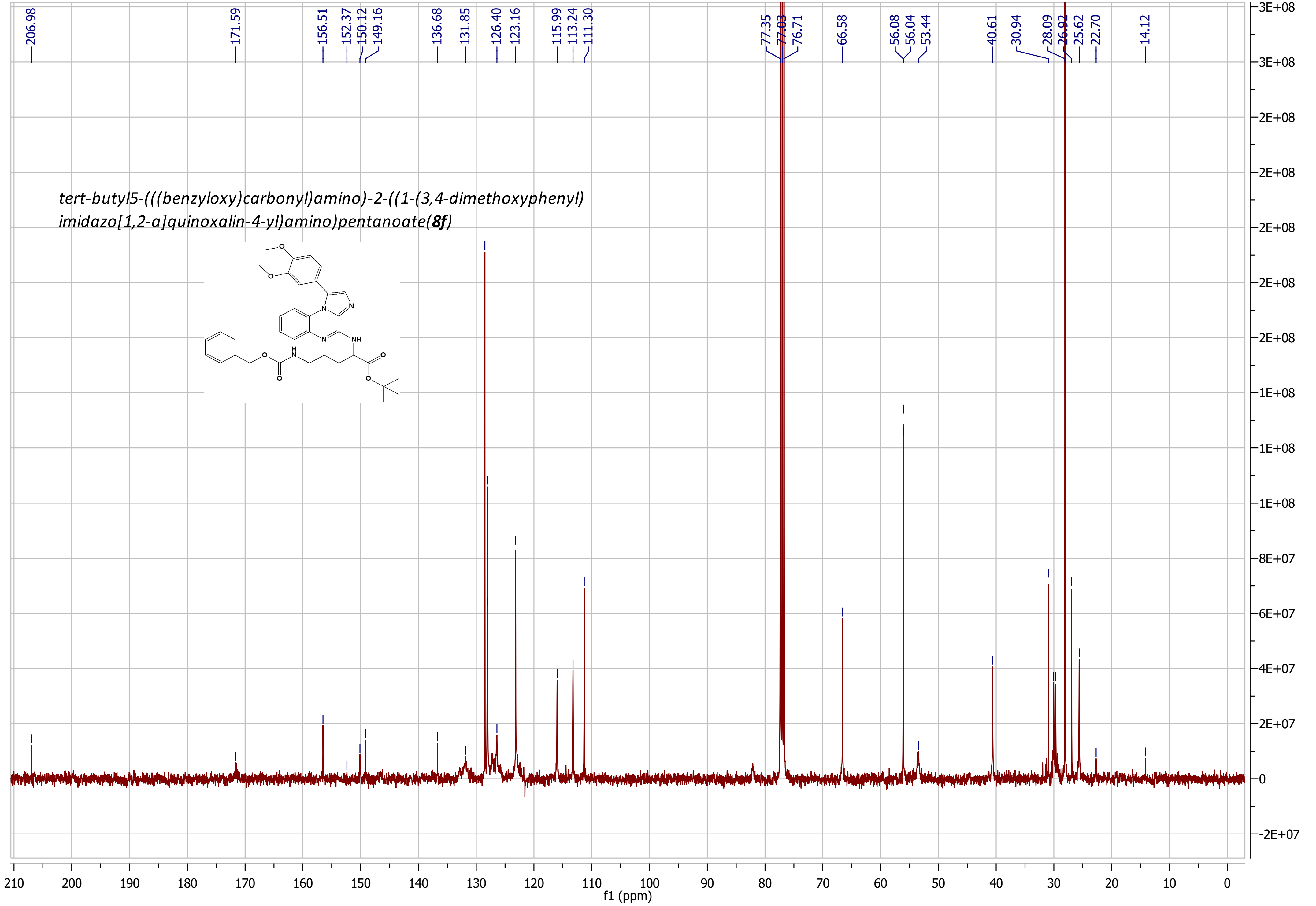

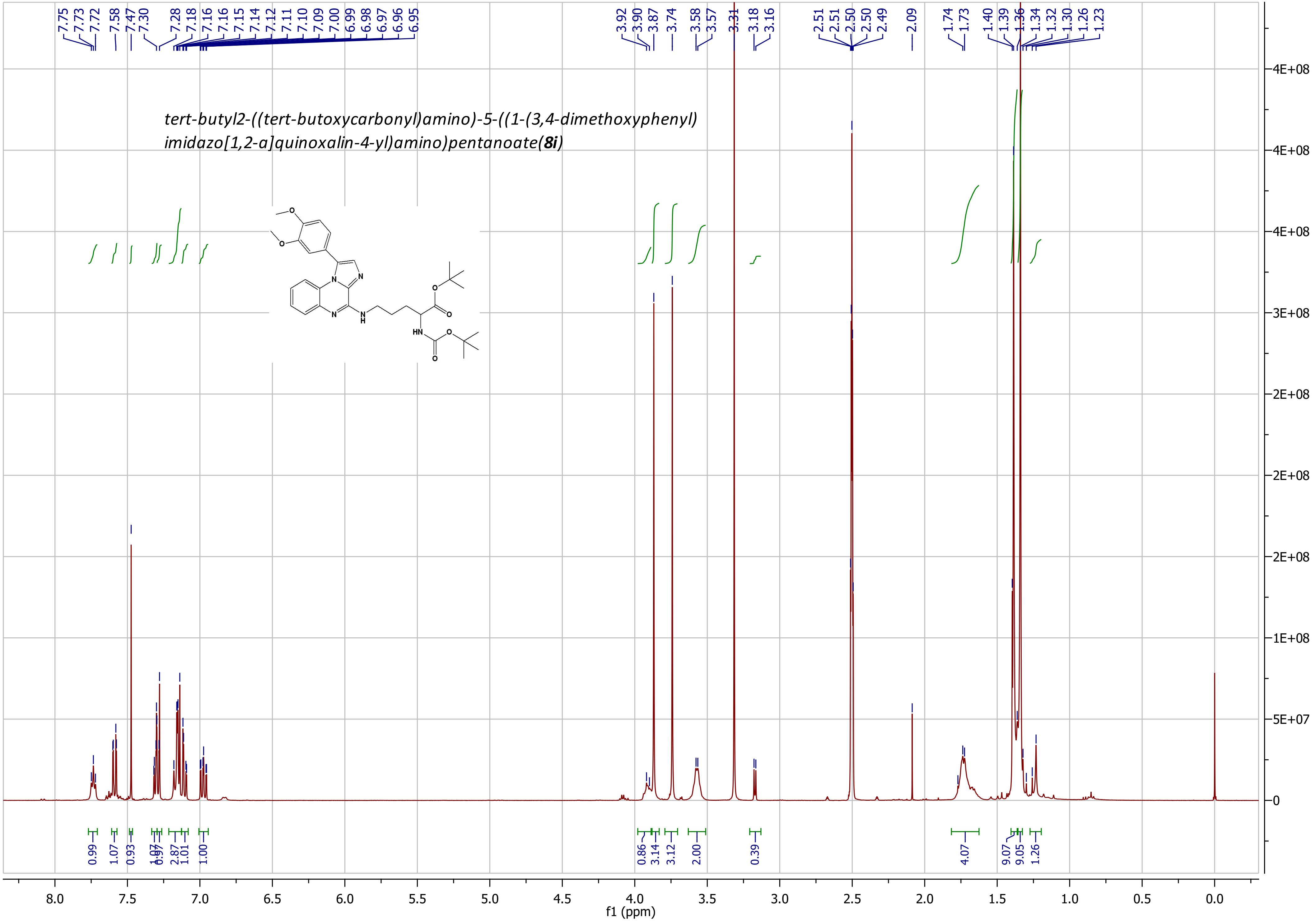
tert-butyl2-((tert-butoxycarbonyl)amino)-5-((1-(3,4-dimethoxyphenyl)
imidazo[1,2-a]quinoxalin-4-yl)amino)pentanoate(**8i**)
7.75 7.73 7.72 7.58 7.47 7.30 7.28 7.18 7.16 7.16 7.15 7.14 7.12 7.11 7.10 7.09 7.00 6.99 6.98 6.97 6.96 6.95
3.92 3.90 3.87 3.74 3.58 3.57 3.31 3.18 3.16
2.51 2.51 2.50 2.50 2.49 2.09
1.74 1.73 1.40 1.39 1.36 1.34 1.32 1.30 1.26 1.23
0.99 1.07 0.93 1.07 0.97 2.87 1.01 1.00
0.86 3.14 3.12 2.00 0.39
4.07 9.07 9.05 1.26
f1 (ppm)

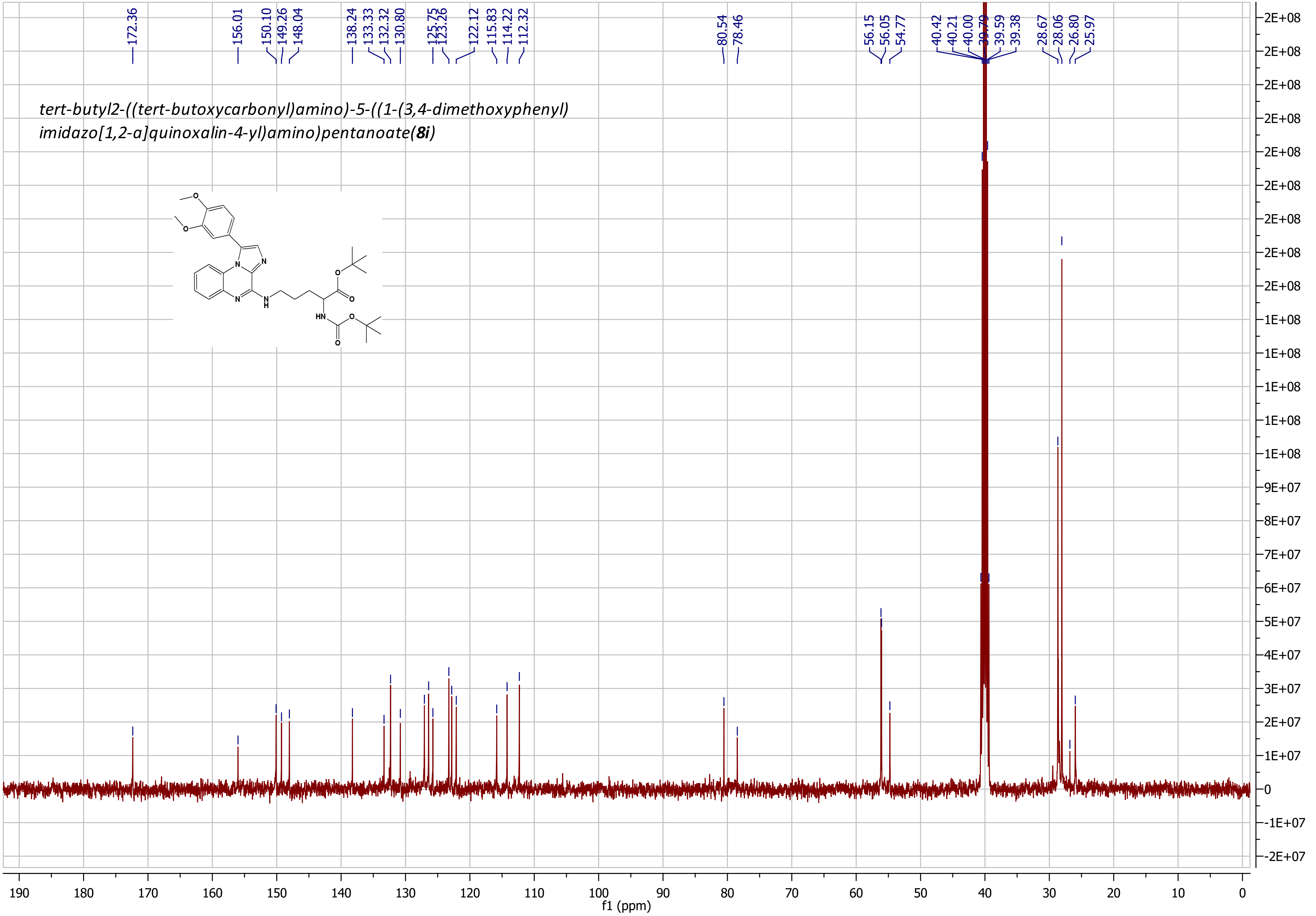
tert-butyl2-((tert-butoxycarbonyl)amino)-5-((1-(3,4-dimethoxyphenyl)
imidazo[1,2-a]quinoxalin-4-yl)amino)pentanoate(8i)
172.36
156.01
150.10
149.26
148.04
138.24
133.33
132.32
130.80
125.75
123.26
122.12
115.83
114.22
112.32
80.54
78.46
56.15
56.05
54.77
40.42
40.21
40.00
39.79
39.59
39.38
28.67
28.06
26.80
25.97
f1 (ppm)

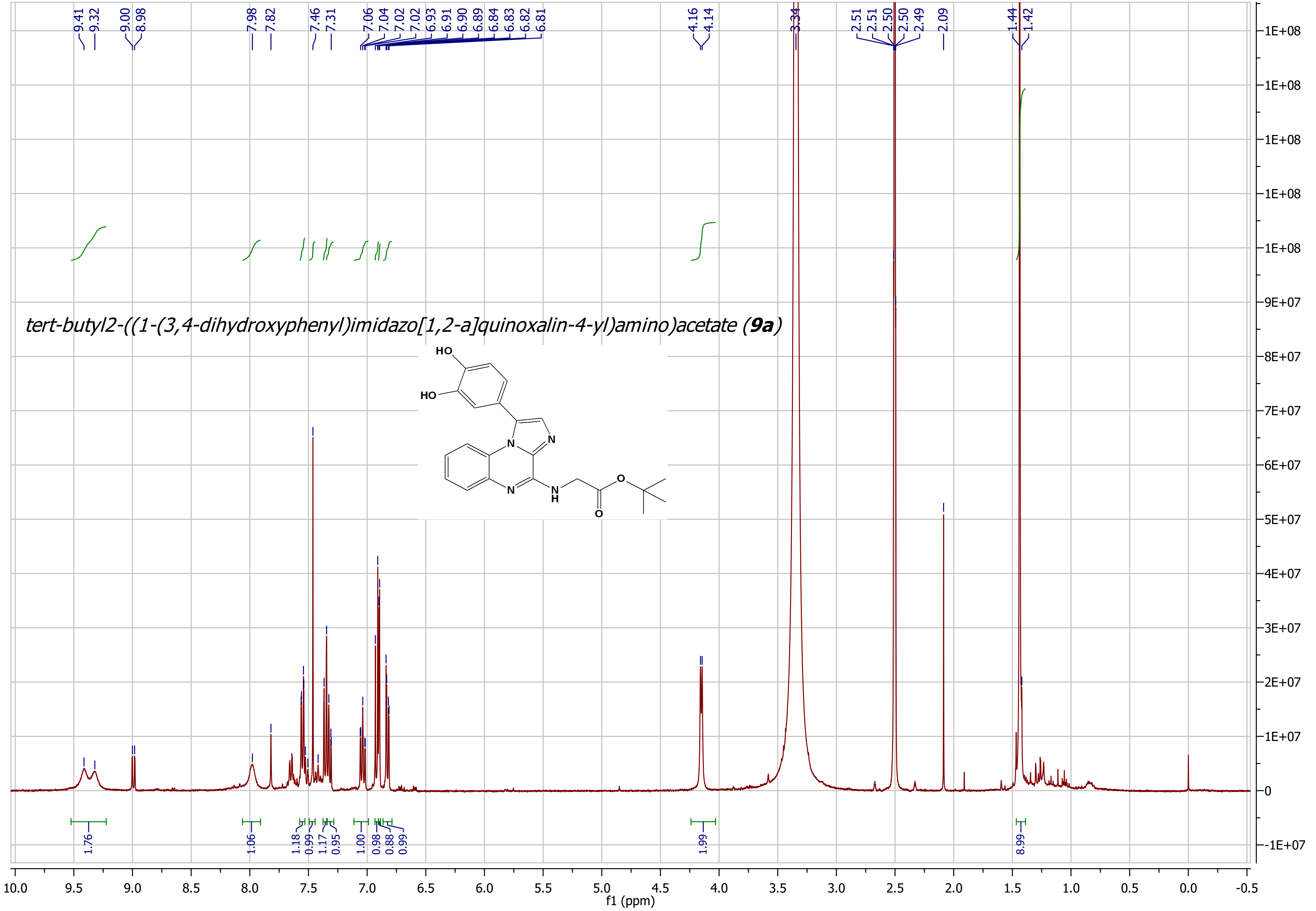
tert-butyl2-((1-(3,4-dihydroxyphenyl)imidazo[1,2-a]quinoxalin-4-yl)amino)acetate (**9a**)
9.41
9.32
9.00
8.98
7.98
7.82
7.46
7.31
7.06
7.04
7.02
7.02
6.93
6.91
6.90
6.89
6.84
6.83
6.82
6.81
4.16
4.14
3.34
2.51
2.51
2.50
2.50
2.49
2.09
1.44
1.42
1.76
1.06
1.18
0.99
1.17
0.95
1.00
0.98
0.88
0.99
1.99
8.99
HO
HO
N
N
N
N
H
O
O
f1 (ppm)

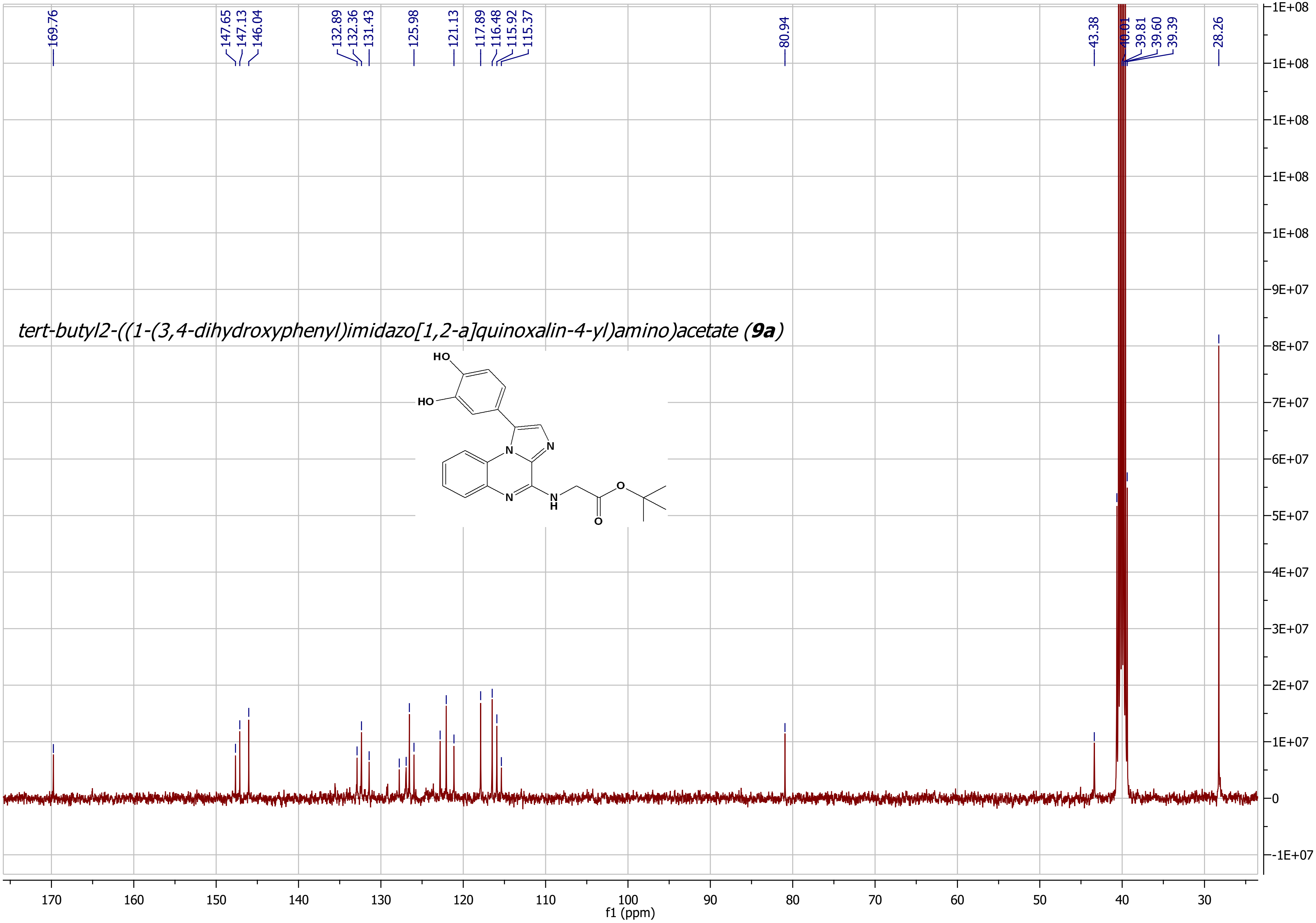
169.76
147.65
147.13
146.04
132.89
132.36
131.43
125.98
121.13
117.89
116.48
115.92
115.37
80.94
43.38
40.01
39.81
39.60
39.39
28.26
tert-butyl2-((1-(3,4-dihydroxyphenyl)imidazo[1,2-a]quinoxalin-4-yl)amino)acetate (**9a**)
HO
HO
N
N
N
N
H
O
O
1E+08
9E+07
8E+07
7E+07
6E+07
5E+07
4E+07
3E+07
2E+07
1E+07
0
-1E+07
170
160
150
140
130
120
110
100
90
80
70
60
50
40
30
f1 (ppm)

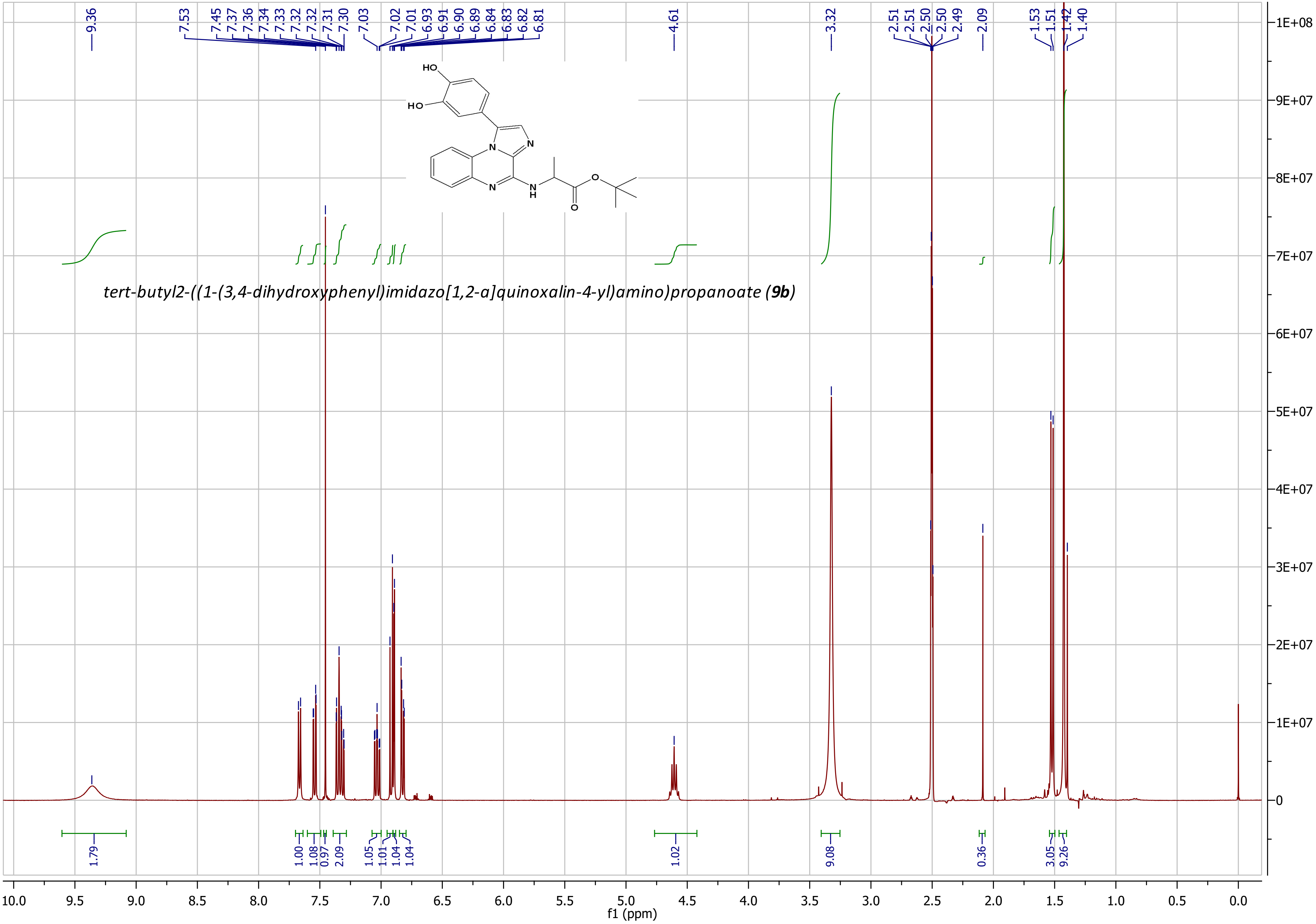
tert-butyl2-((1-(3,4-dihydroxyphenyl)imidazo[1,2-a]quinoxalin-4-yl)amino)propanoate (**9b**)
9.36
7.53 7.45 7.37 7.36 7.34 7.33 7.32 7.32 7.31 7.30 7.03 7.02 7.01 6.93 6.91 6.90 6.89 6.84 6.83 6.82 6.81
4.61
3.32
2.51 2.51 2.50 2.50 2.49 2.09
1.53 1.51 1.42 1.40
1.79
1.00 1.08 0.97 2.09 1.05 1.01 1.04 1.04
1.02
9.08
0.36
3.05 9.26
f1 (ppm)

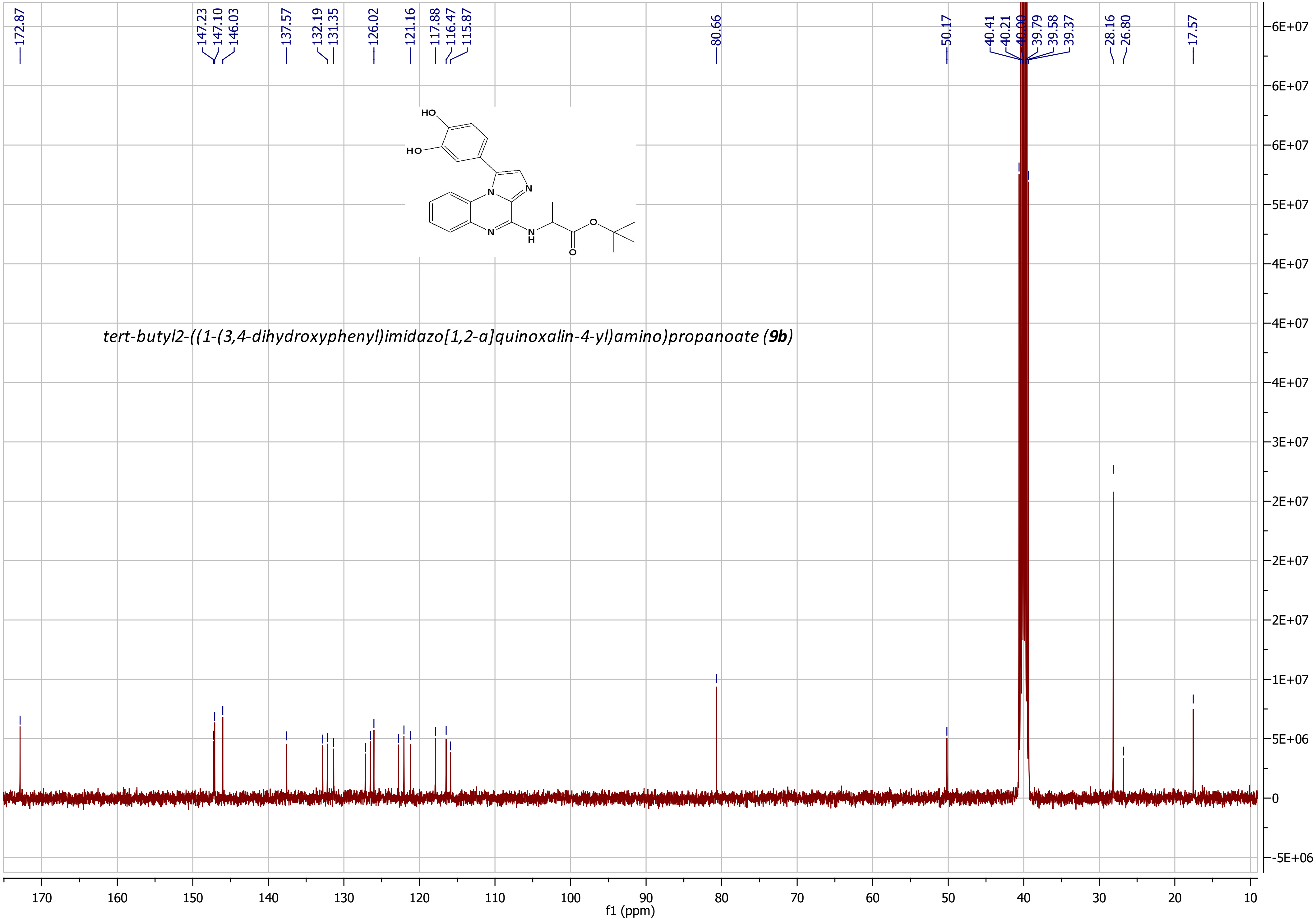

*tert-butyl2-((1-(3,4-dihydroxyphenyl)imidazo[1,2-a]quinoxalin-4-yl)amino)propanoate (**9b**)*

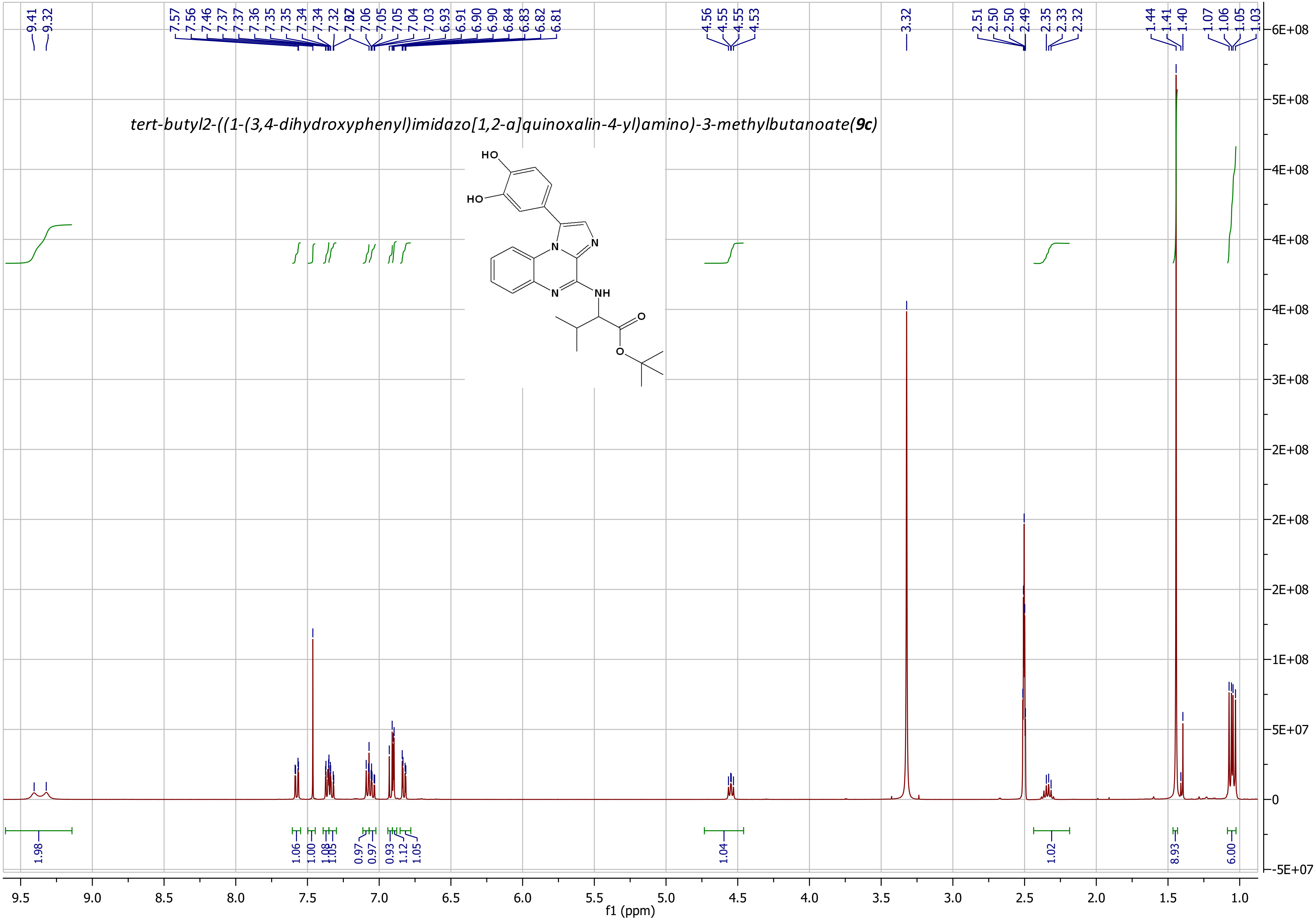
tert-butyl2-((1-(3,4-dihydroxyphenyl)imidazo[1,2-a]quinoxalin-4-yl)amino)-3-methylbutanoate(**9c**)
HO
HO
N
N
N
NH
O
O
9.41
9.32
7.57
7.56
7.46
7.37
7.37
7.36
7.35
7.35
7.34
7.34
7.32
7.06
7.05
7.05
7.04
7.03
6.93
6.91
6.90
6.90
6.84
6.83
6.82
6.81
4.56
4.55
4.55
4.53
3.32
2.51
2.50
2.50
2.49
2.35
2.33
2.32
1.44
1.41
1.40
1.07
1.06
1.05
1.03
1.98
1.06
1.00
1.08
1.05
0.97
0.97
0.93
1.12
1.05
1.04
1.02
8.93
6.00
f1 (ppm)

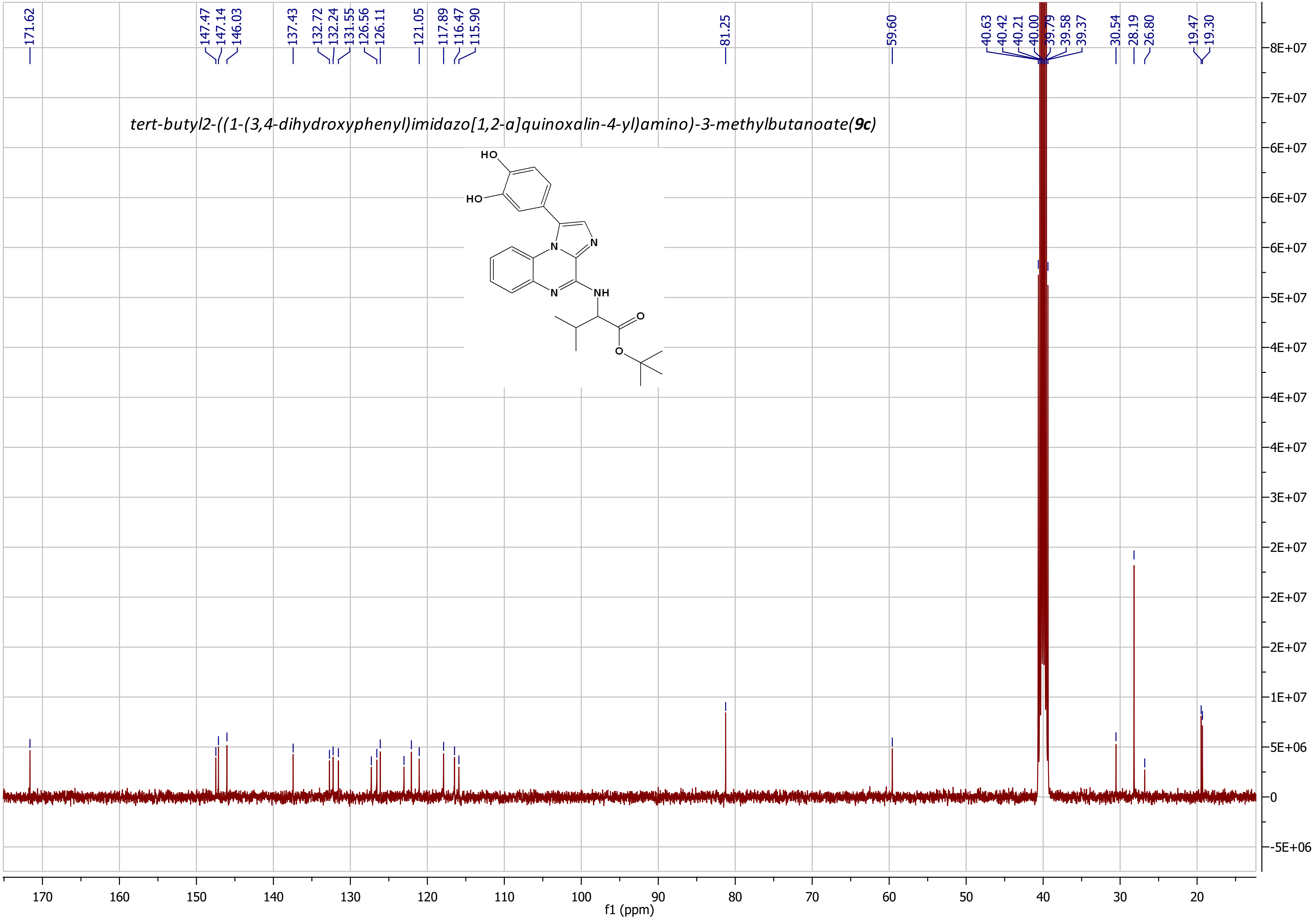
tert-butyl2-((1-(3,4-dihydroxyphenyl)imidazo[1,2-a]quinoxalin-4-yl)amino)-3-methylbutanoate(**9c**)
171.62
147.47
147.14
146.03
137.43
132.72
132.24
131.55
126.56
126.11
121.05
117.89
116.47
115.90
81.25
59.60
40.63
40.42
40.21
40.00
39.79
39.58
39.37
30.54
28.19
26.80
19.47
19.30
HO
HO
N
N
N
NH
O
O
8E+07
7E+07
6E+07
6E+07
6E+07
5E+07
4E+07
4E+07
4E+07
3E+07
2E+07
2E+07
2E+07
1E+07
5E+06
0
-5E+06
170
160
150
140
130
120
110
100
90
80
70
60
50
40
30
20
f1 (ppm)

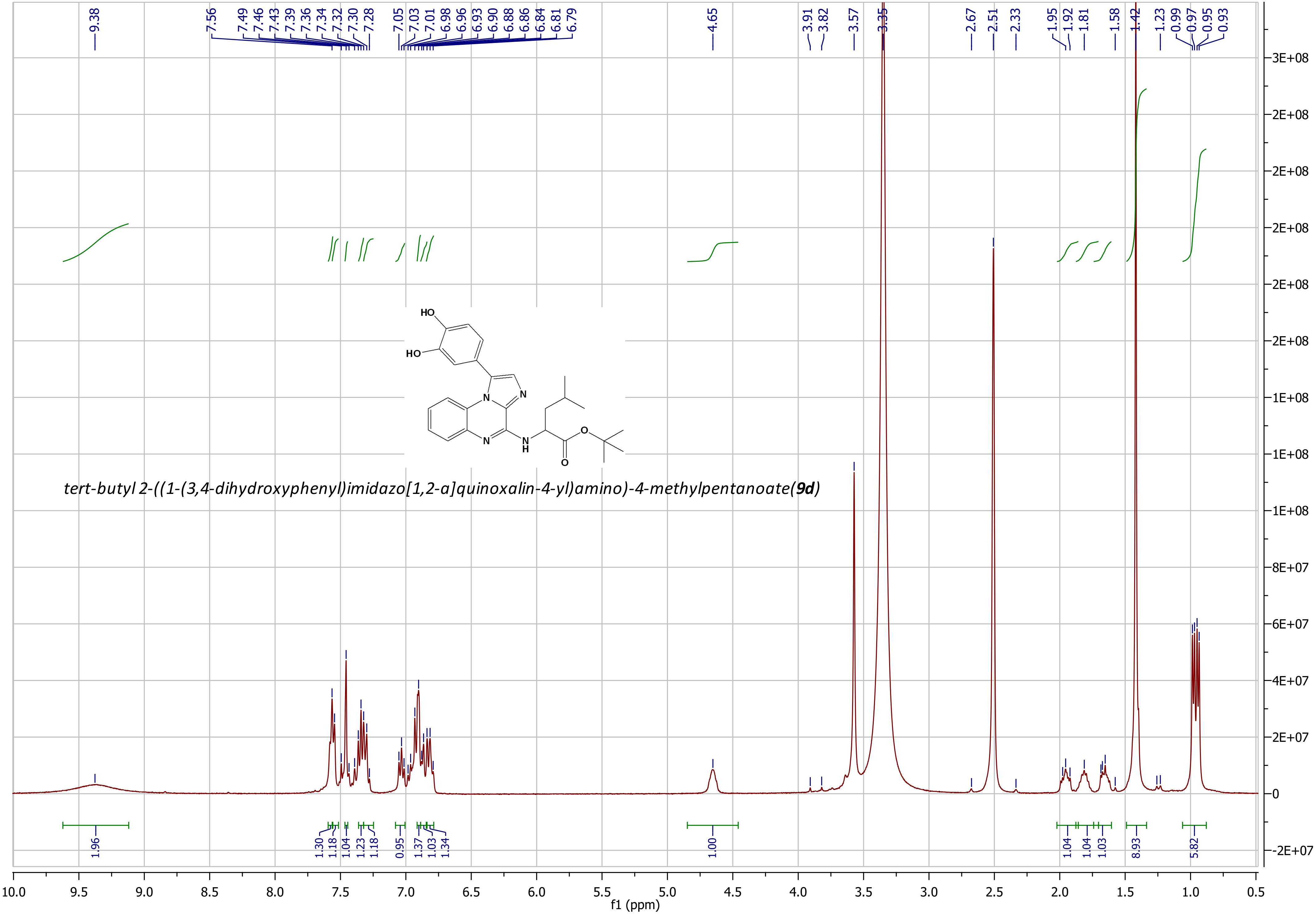

*tert-butyl 2-((1-(3,4-dihydroxyphenyl)imidazo[1,2-a]quinoxalin-4-yl)amino)-4-methylpentanoate(**9d**)*

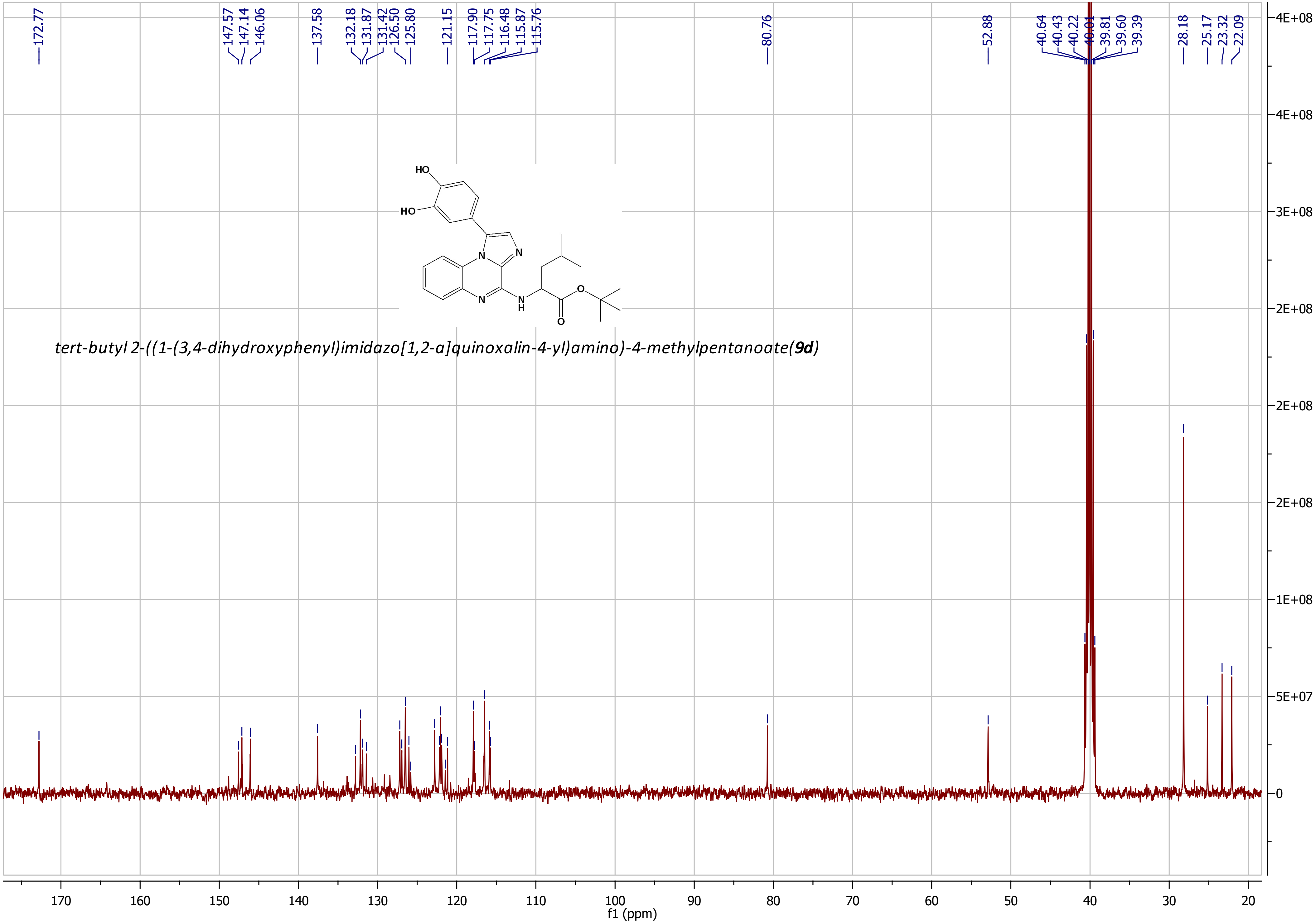
172.77
147.57
147.14
146.06
137.58
132.18
131.87
131.42
126.50
125.80
121.15
117.90
117.75
116.48
115.87
115.76
80.76
52.88
40.64
40.43
40.22
40.01
39.81
39.60
39.39
28.18
25.17
23.32
22.09
tert-butyl 2-((1-(3,4-dihydroxyphenyl)imidazo[1,2-a]quinoxalin-4-yl)amino)-4-methylpentanoate(9d)
f1 (ppm)

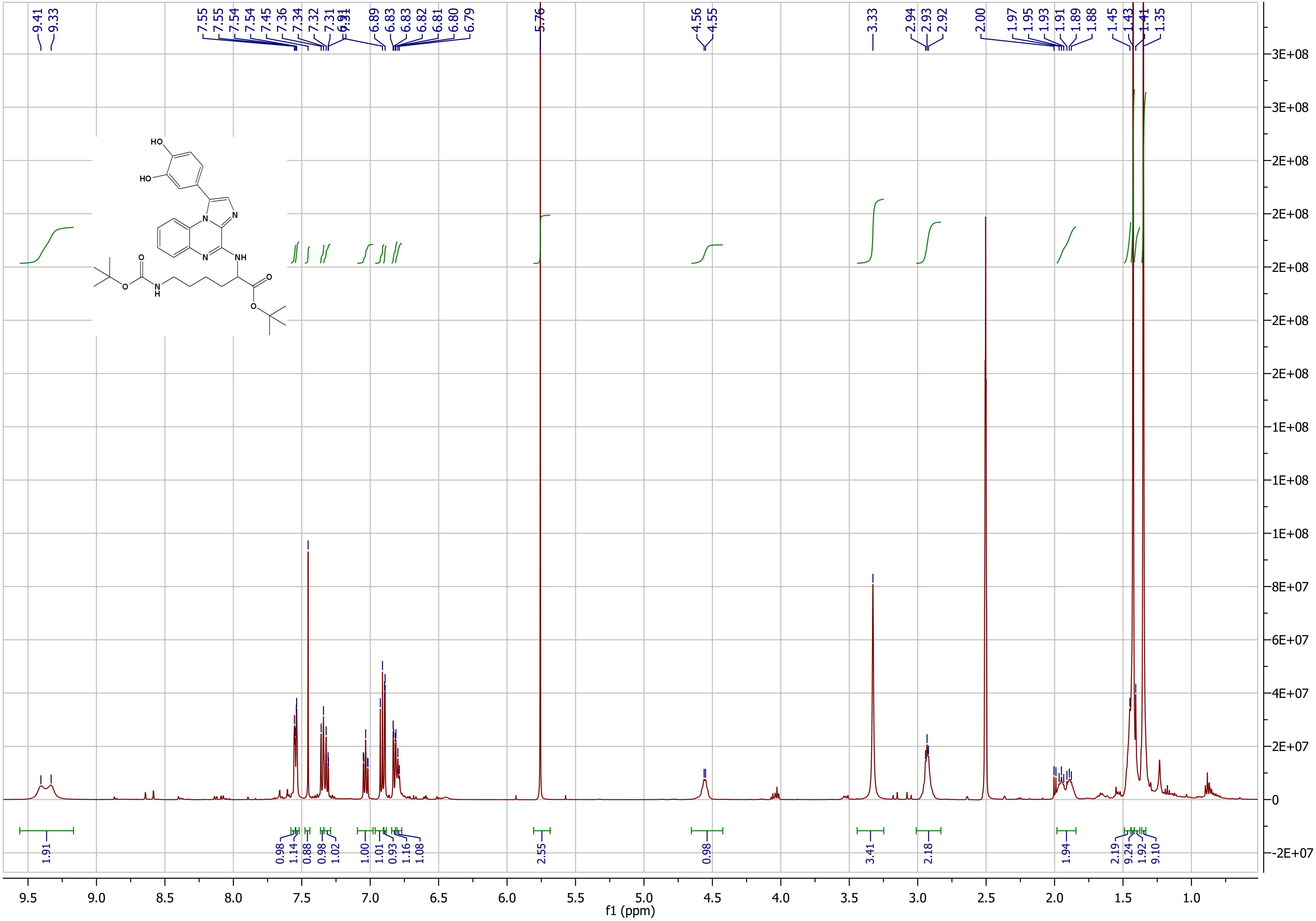
9.41
9.33
7.55
7.55
7.54
7.54
7.45
7.36
7.34
7.32
7.31
6.91
7.31
6.89
6.83
6.83
6.82
6.81
6.80
6.79
5.76
4.56
4.55
3.33
2.94
2.93
2.92
2.00
1.97
1.95
1.93
1.91
1.89
1.88
1.45
1.43
1.41
1.35
HO
HO
N
N
N
N
NH
O
O
N
H
O
O
1.91
0.98
1.14
0.88
0.98
1.02
1.00
1.01
0.93
1.16
1.08
2.55
0.98
3.41
2.18
1.94
2.19
9.24
1.92
9.10
3E+08
3E+08
2E+08
2E+08
2E+08
2E+08
2E+08
1E+08
1E+08
1E+08
8E+07
6E+07
4E+07
2E+07
0
-2E+07
9.5
9.0
8.5
8.0
7.5
7.0
6.5
6.0
5.5
5.0
4.5
4.0
3.5
3.0
2.5
2.0
1.5
1.0
f1 (ppm)

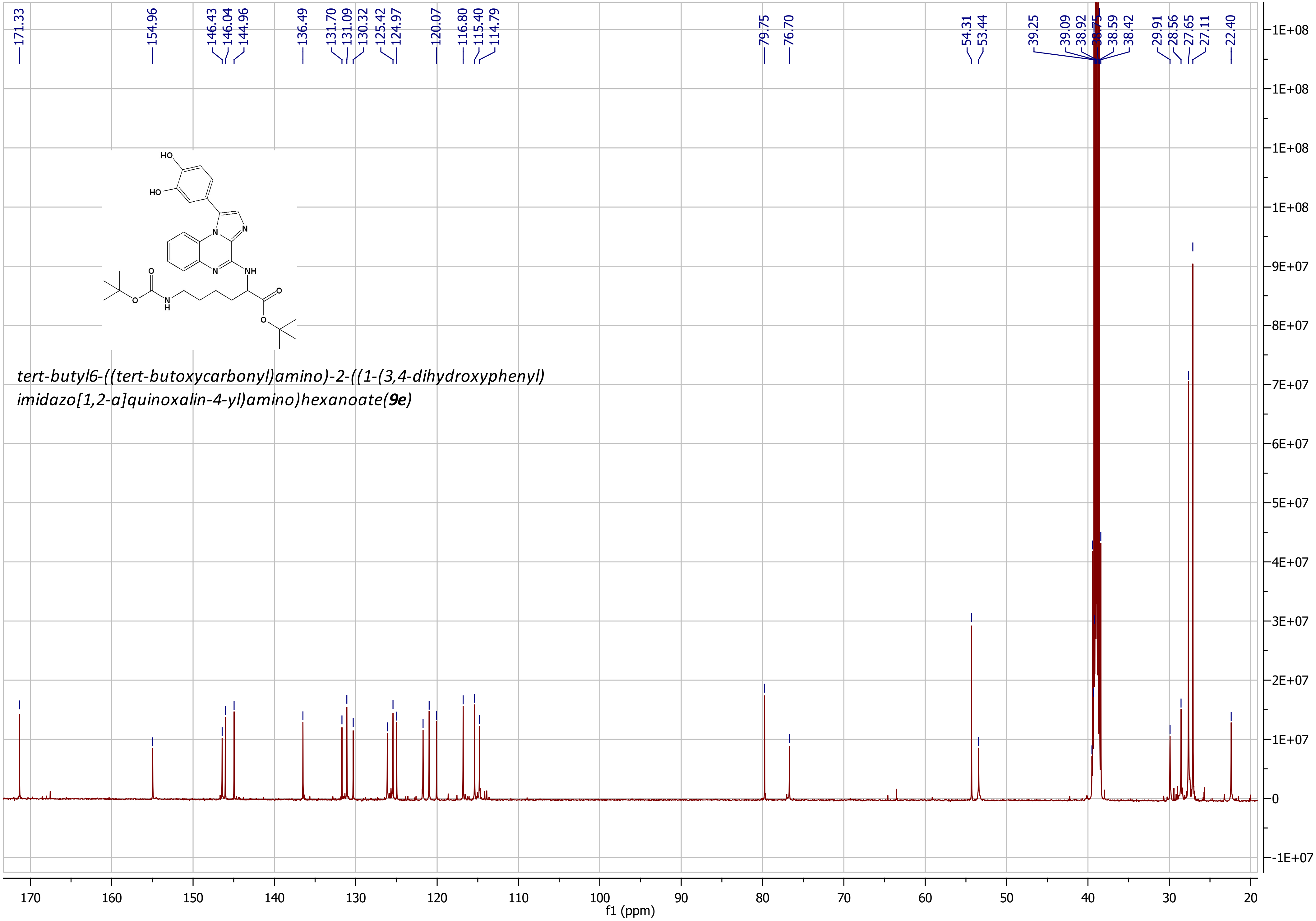

*tert-butyl6-((tert-butoxycarbonyl)amino)-2-((1-(3,4-dihydroxyphenyl)imidazo[1,2-a]quinoxalin-4-yl)amino)hexanoate(**9e**)*

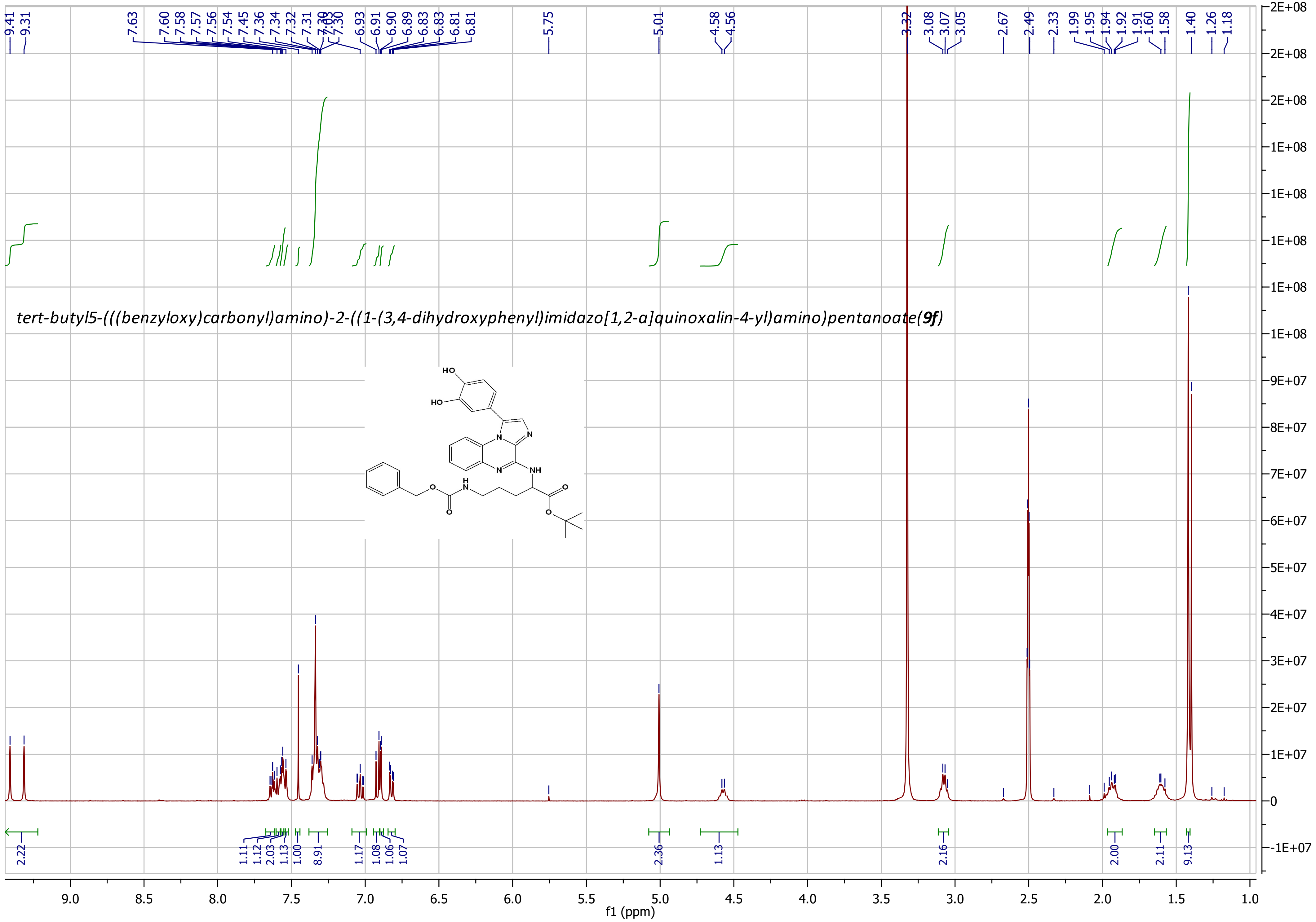
tert-butyl5-(((benzyloxy)carbonyl)amino)-2-((1-(3,4-dihydroxyphenyl)imidazo[1,2-a]quinoxalin-4-yl)amino)pentanoate(9f)
HO
HO
N
N
N
NH
H
N
O
O
O
O
9.41 9.31 7.63 7.60 7.58 7.57 7.56 7.54 7.45 7.36 7.34 7.32 7.31 7.30 7.30 6.93 6.91 6.90 6.89 6.83 6.83 6.81 6.81 5.75 5.01 4.58 4.56 3.32 3.08 3.07 3.05 2.67 2.49 2.33 1.99 1.95 1.94 1.92 1.91 1.60 1.58 1.40 1.26 1.18
2.22 1.11 1.12 2.03 1.13 1.00 8.91 1.17 1.08 1.06 1.07 2.36 1.13 2.16 2.00 2.11 9.13
f1 (ppm)

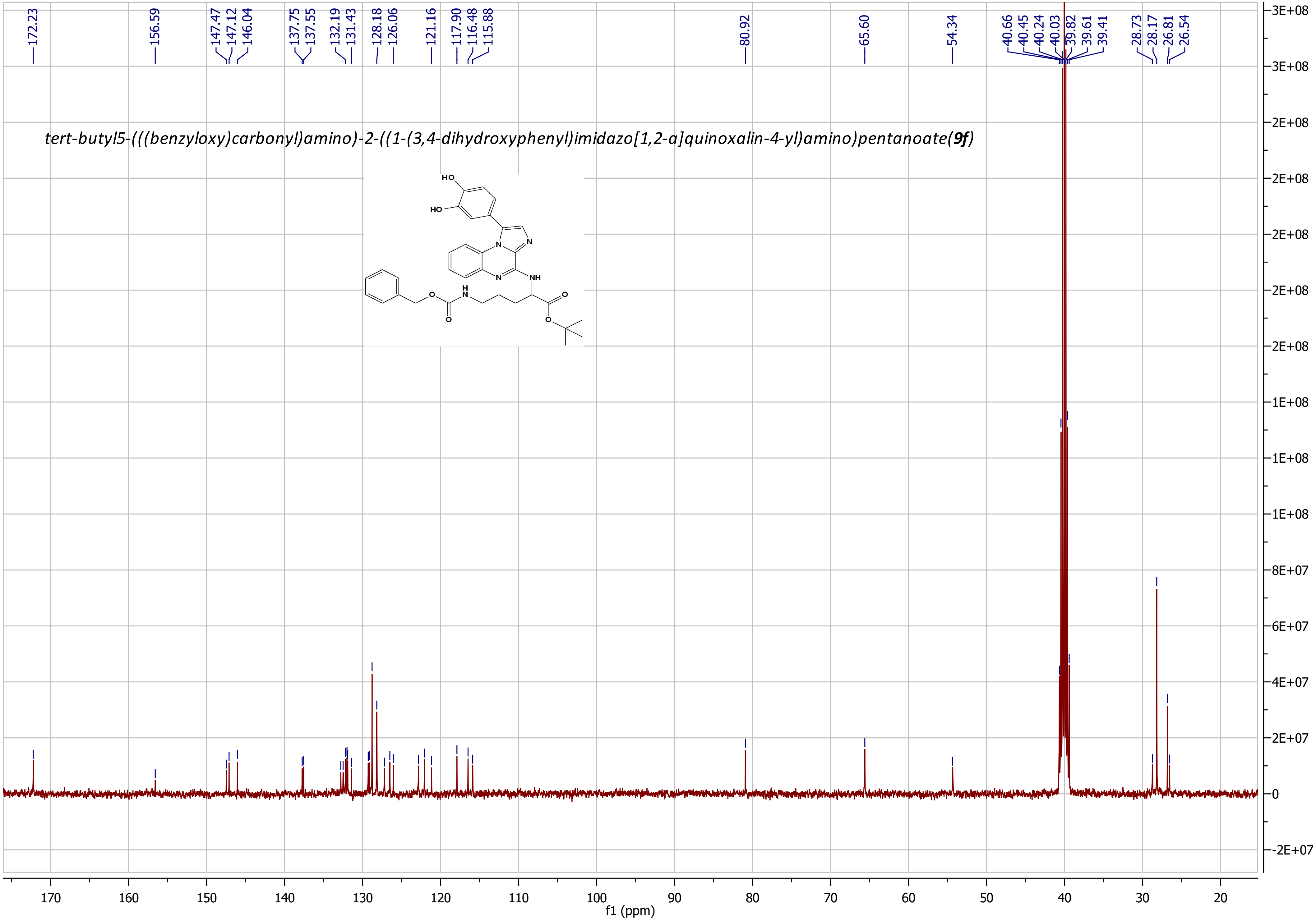
172.23
156.59
147.47
147.12
146.04
137.75
137.55
132.19
131.43
128.18
126.06
121.16
117.90
116.48
115.88
80.92
65.60
54.34
40.66
40.45
40.24
40.03
39.82
39.61
39.41
28.73
28.17
26.81
26.54
tert-butyl5-(((benzyloxy)carbonyl)amino)-2-((1-(3,4-dihydroxyphenyl)imidazo[1,2-a]quinoxalin-4-yl)amino)pentanoate(9f)
HO
HO
N
N
N
NH
H
N
O
O
O
O
3E+08
3E+08
2E+08
2E+08
2E+08
2E+08
2E+08
1E+08
1E+08
1E+08
8E+07
6E+07
4E+07
2E+07
0
-2E+07
170
160
150
140
130
120
110
100
90
80
70
60
50
40
30
20
f1 (ppm)

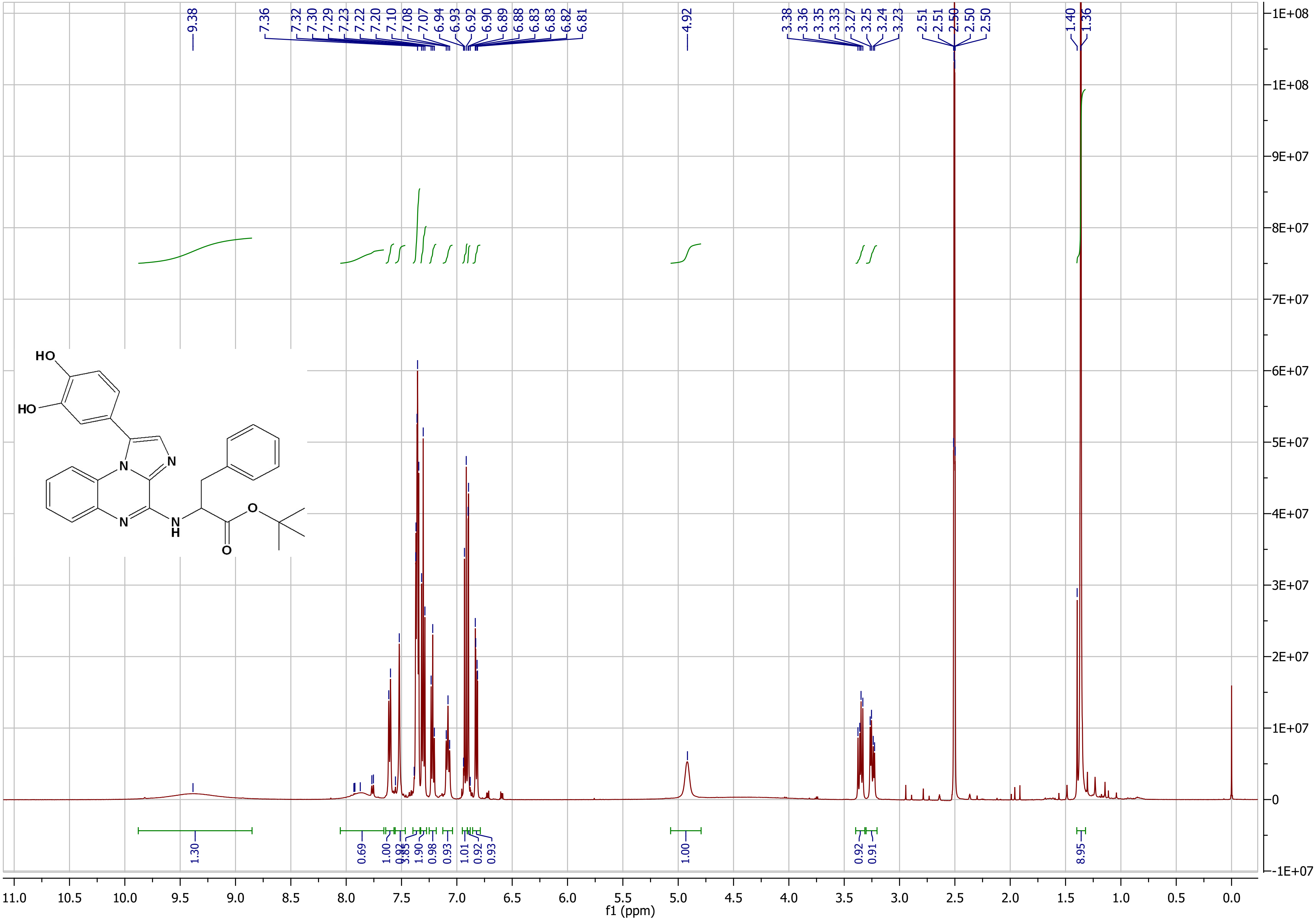
9.38
7.36 7.32 7.30 7.29 7.23 7.22 7.20 7.10 7.08 7.07 6.94 6.93 6.92 6.90 6.89 6.88 6.83 6.83 6.82 6.81
4.92
3.38 3.36 3.35 3.33 3.27 3.25 3.24 3.23
2.51 2.51 2.50 2.50 2.50
1.40 1.36
HO
HO
N
N
N
N
H
O
O
1.30
0.69 1.00 0.92 3.85 1.90 0.98 0.93 1.01 0.92 0.93
1.00
0.92 0.91
8.95
11.0 10.5 10.0 9.5 9.0 8.5 8.0 7.5 7.0 6.5 6.0 5.5 5.0 4.5 4.0 3.5 3.0 2.5 2.0 1.5 1.0 0.5 0.0
f1 (ppm)
1E+08 1E+08 9E+07 8E+07 7E+07 6E+07 5E+07 4E+07 3E+07 2E+07 1E+07 0 -1E+07

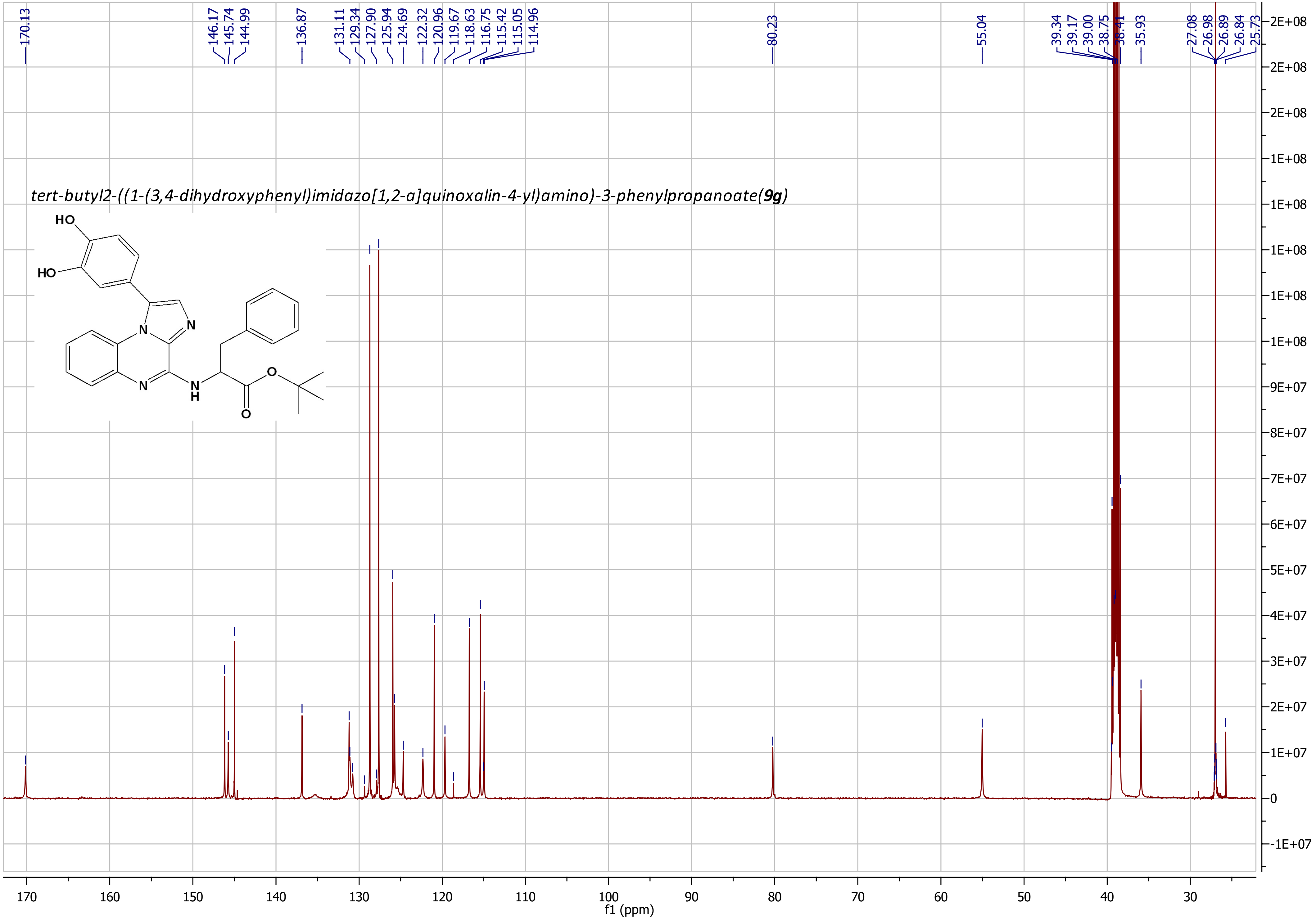

tert-butyl2-((1-(3,4-dihydroxyphenyl)imidazo[1,2-a]quinoxalin-4-yl)amino)-3-phenylpropanoate(**9g**)
HO
HO
N
N
N
N
H
O
O
170.13
146.17
145.74
144.99
136.87
131.11
129.34
127.90
125.94
124.69
122.32
120.96
119.67
118.63
116.75
115.42
115.05
114.96
80.23
55.04
39.34
39.17
39.00
38.75
38.41
35.93
27.08
26.98
26.89
26.84
25.73
f1 (ppm)

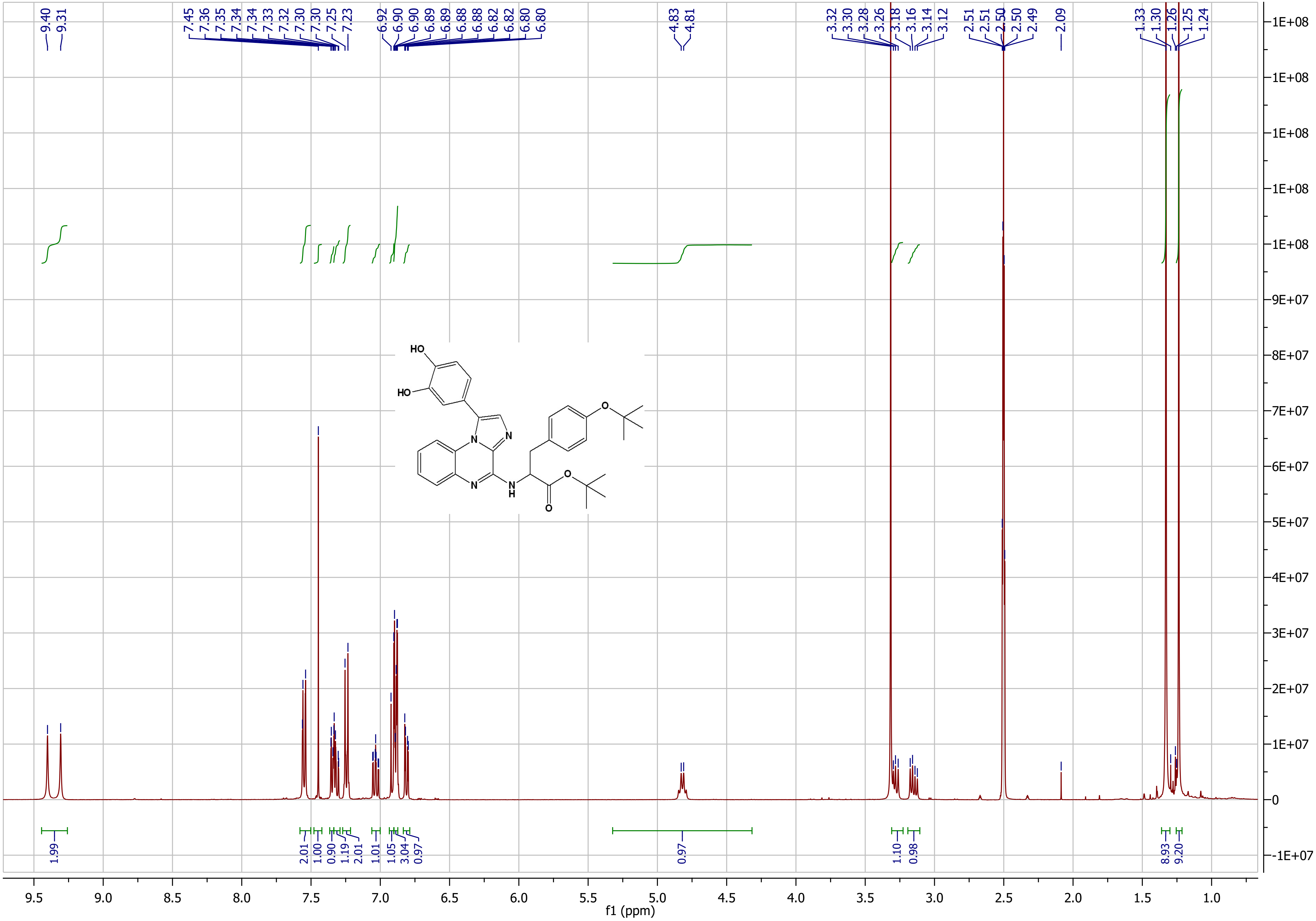
9.40
9.31
7.45
7.36
7.35
7.34
7.34
7.33
7.32
7.30
7.30
7.25
7.23
6.92
6.90
6.90
6.89
6.89
6.88
6.88
6.82
6.82
6.80
6.80
4.83
4.81
3.32
3.30
3.28
3.26
3.18
3.16
3.14
3.12
2.51
2.51
2.50
2.50
2.49
2.09
1.33
1.30
1.26
1.25
1.24
1.99
2.01
1.00
0.90
1.19
2.01
1.01
1.05
3.04
0.97
0.97
1.10
0.98
8.93
9.20
HO
HO
N
N
N
N
H
O
O
O
f1 (ppm)

*tert-butyl3-(4-(tert-butoxy)phenyl)-2-((1-(3,4-dihydroxyphenyl)imidazo[1,2-a]quinoxalin-4-yl)amino)propanoate(**9h**)*

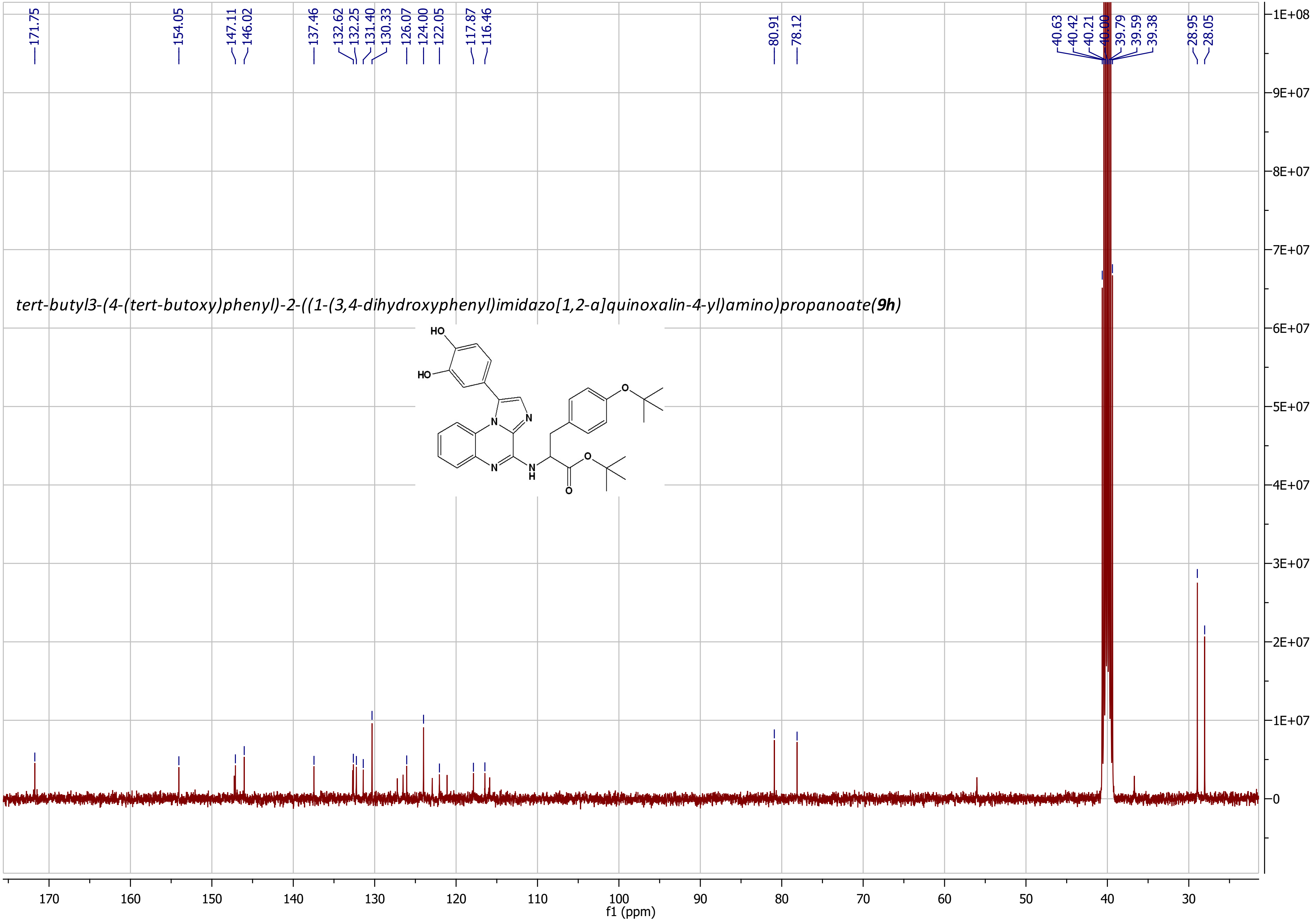

*tert-butyl2-((tert-butoxycarbonyl)amino)-5-((1-(3,4-dihydroxyphenyl)imidazo[1,2-a]quinoxalin-4-yl)amino)pentanoate(**9i**)*

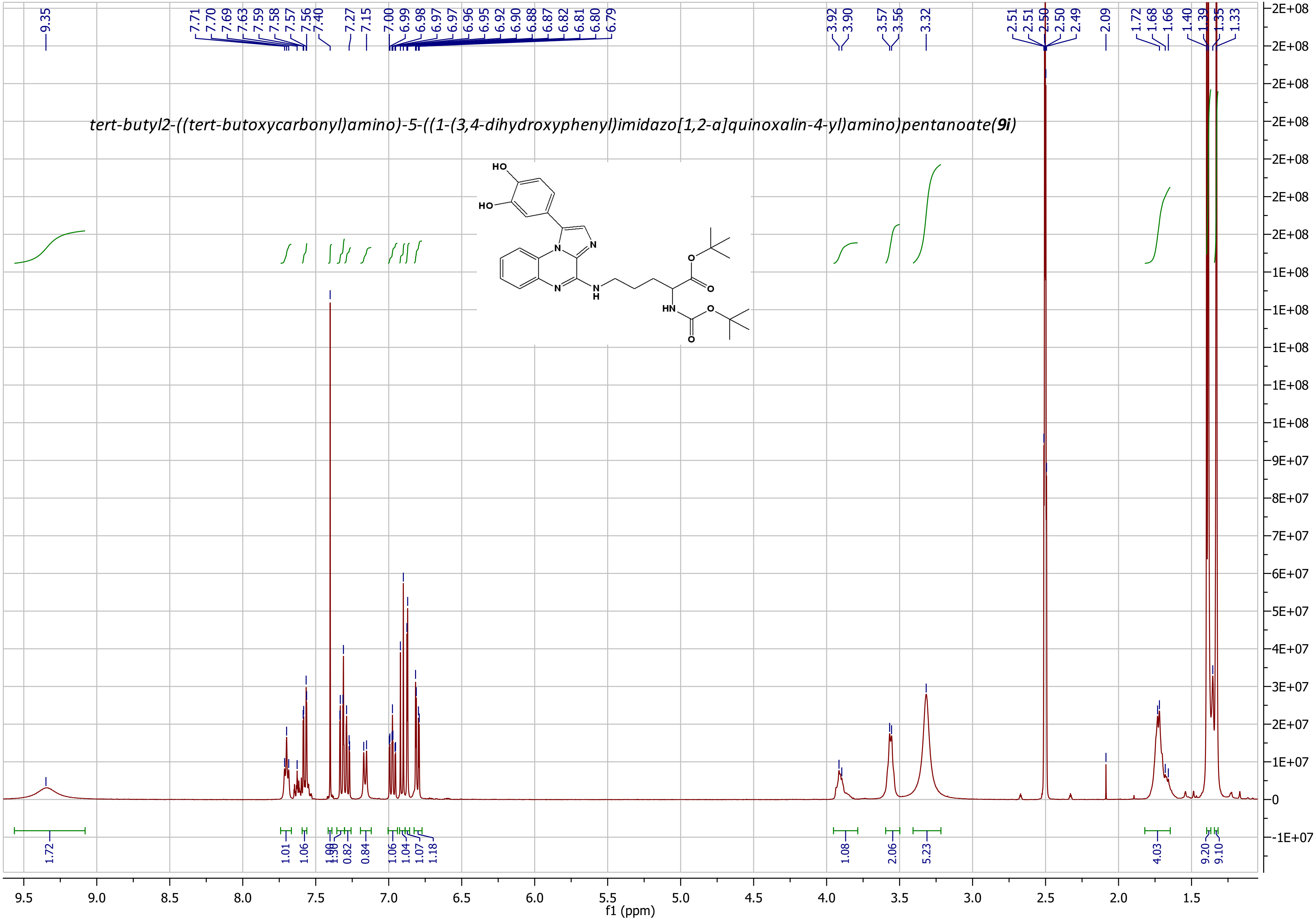

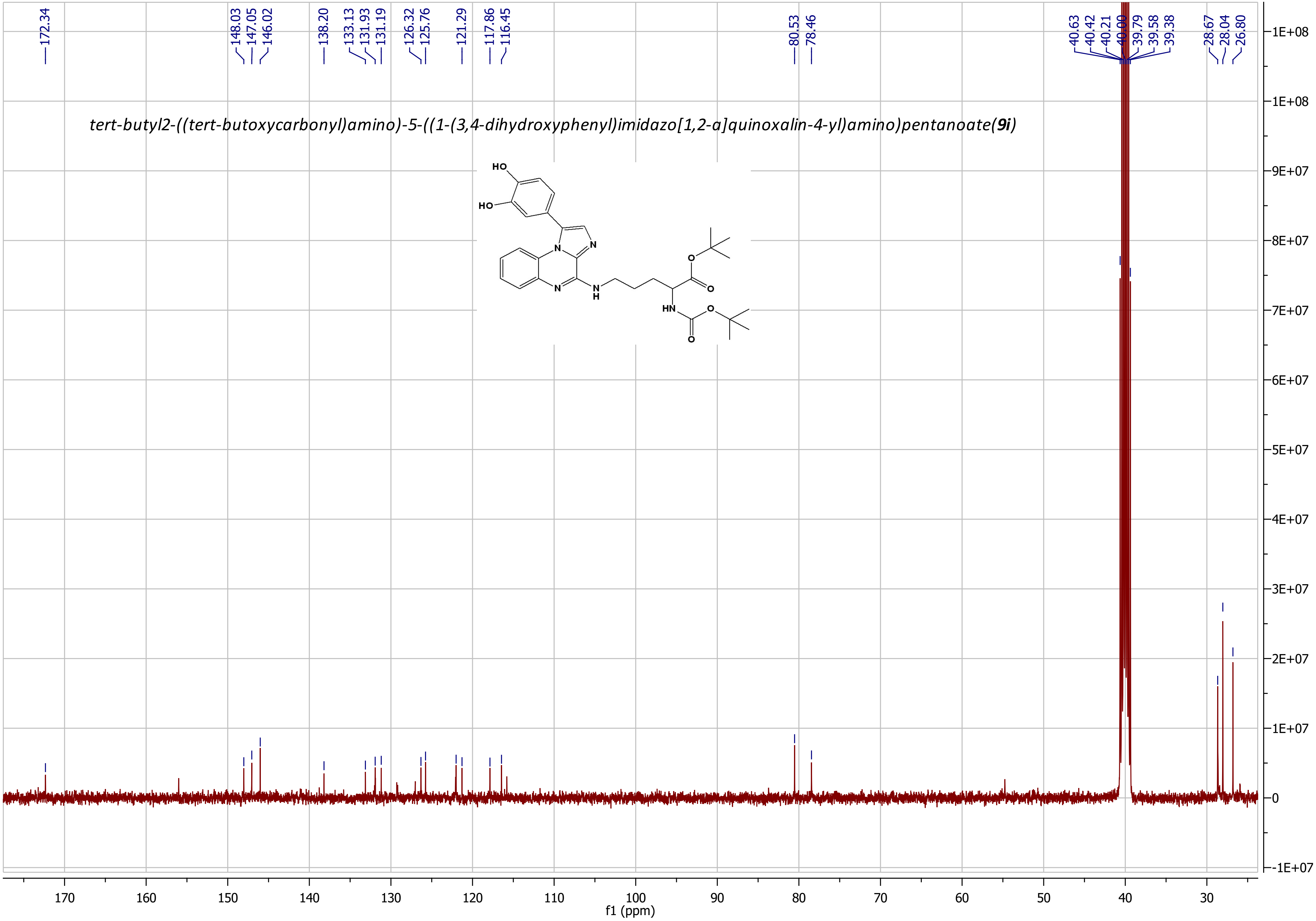
172.34
148.03
147.05
146.02
138.20
133.13
131.93
131.19
126.32
125.76
121.29
117.86
116.45
80.53
78.46
40.63
40.42
40.21
40.00
39.79
39.58
39.38
28.67
28.04
26.80
tert-butyl2-((tert-butoxycarbonyl)amino)-5-((1-(3,4-dihydroxyphenyl)imidazo[1,2-a]quinoxalin-4-yl)amino)pentanoate(**9i**)
HO
HO
N
N
N
N
H
O
O
HN
O
O
1E+08
1E+08
9E+07
8E+07
7E+07
6E+07
5E+07
4E+07
3E+07
2E+07
1E+07
0
-1E+07
170
160
150
140
130
120
110
100
90
80
70
60
50
40
30
f1 (ppm)

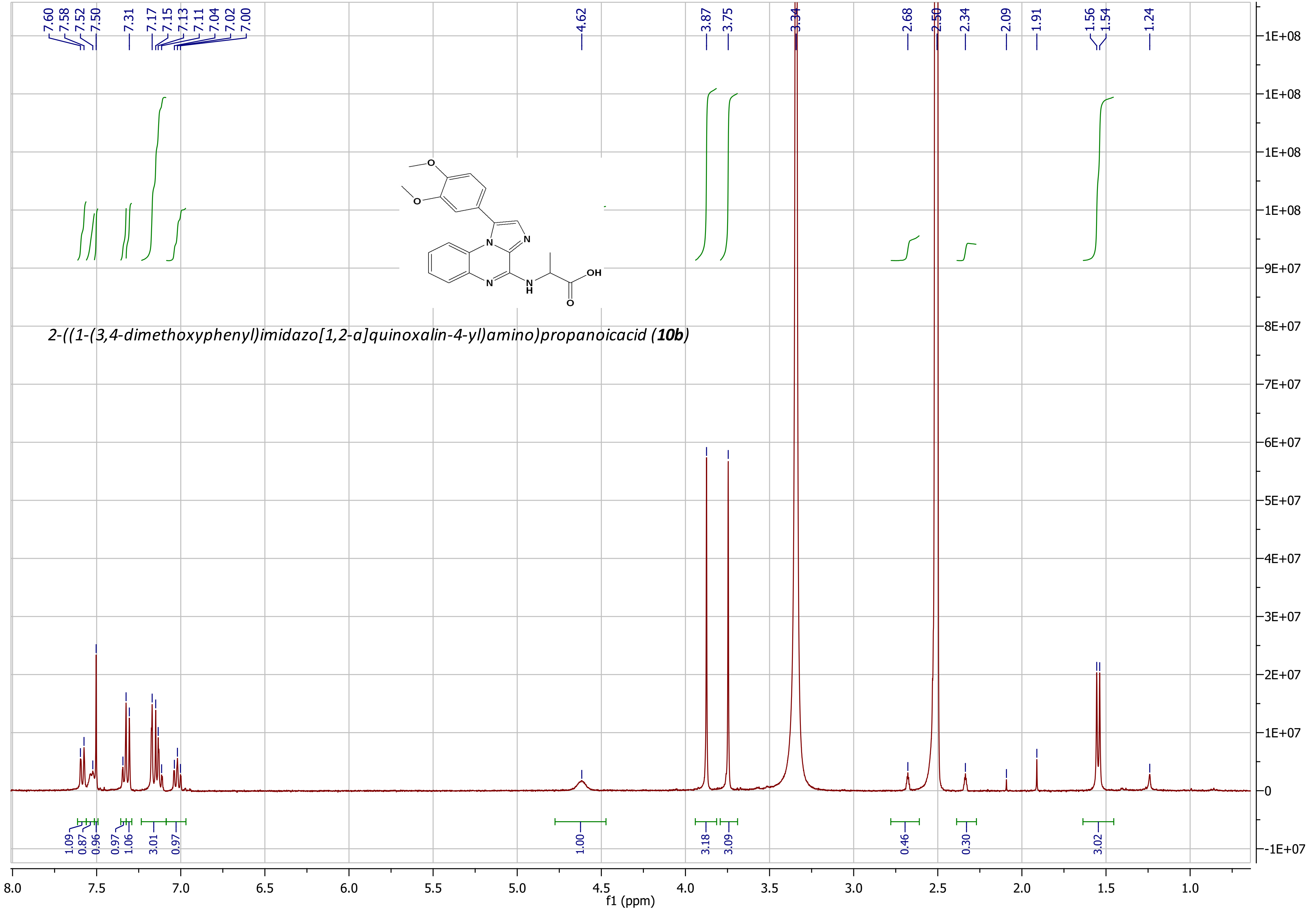

2-((1-(3,4-dimethoxyphenyl)imidazo[1,2-a]quinoxalin-4-yl)amino)propanoicacid (**10b**)
7.60
7.58
7.52
7.50
7.31
7.17
7.15
7.13
7.11
7.04
7.02
7.00
4.62
3.87
3.75
3.34
2.68
2.50
2.34
2.09
1.91
1.56
1.54
1.24
1.09
0.87
0.96
0.97
1.06
3.01
0.97
1.00
3.18
3.09
0.46
0.30
3.02
f1 (ppm)

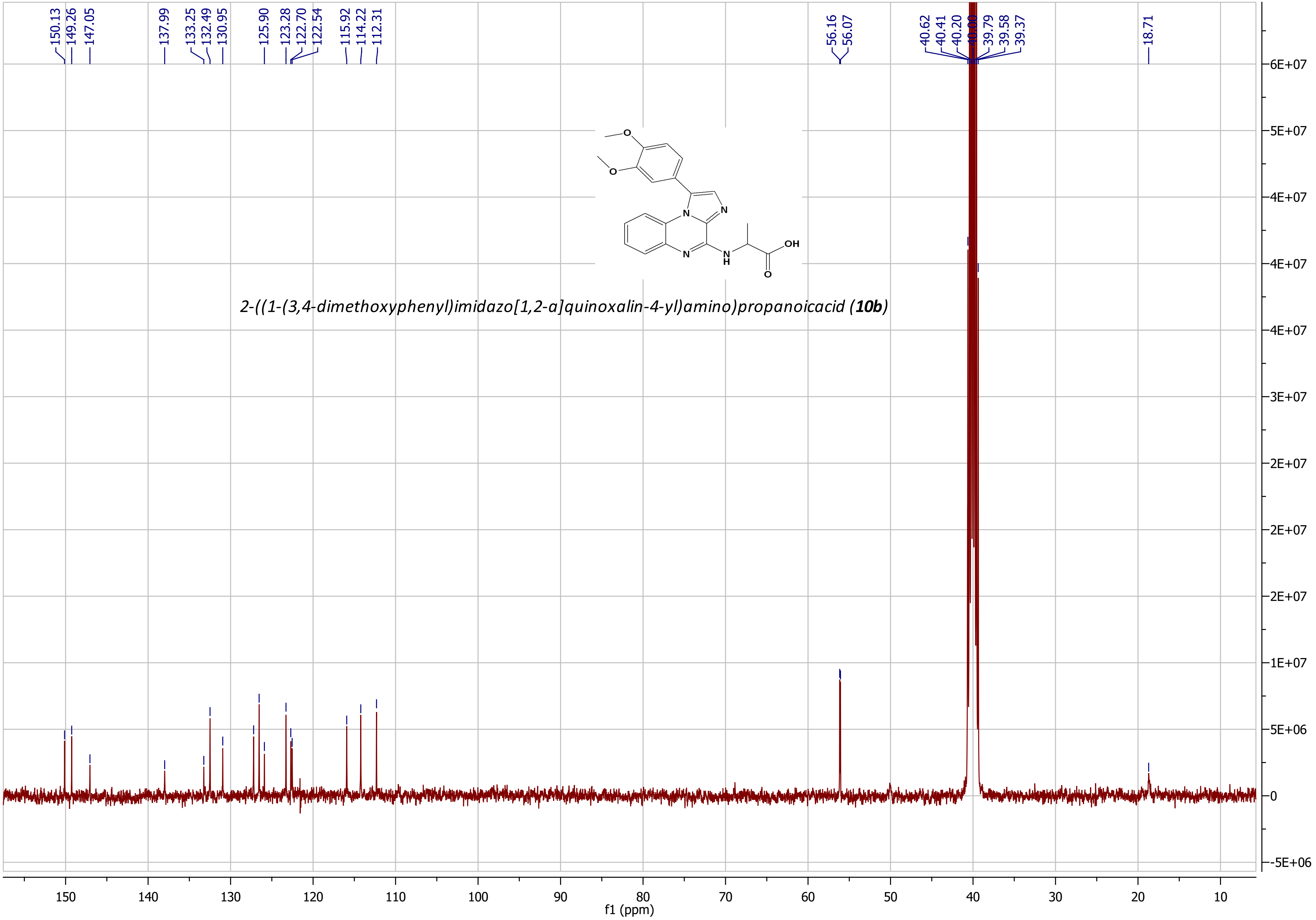

*2-((1-(3,4-dimethoxyphenyl)imidazo[1,2-a]quinoxalin-4-yl)amino)propanoicacid (**10b**)*

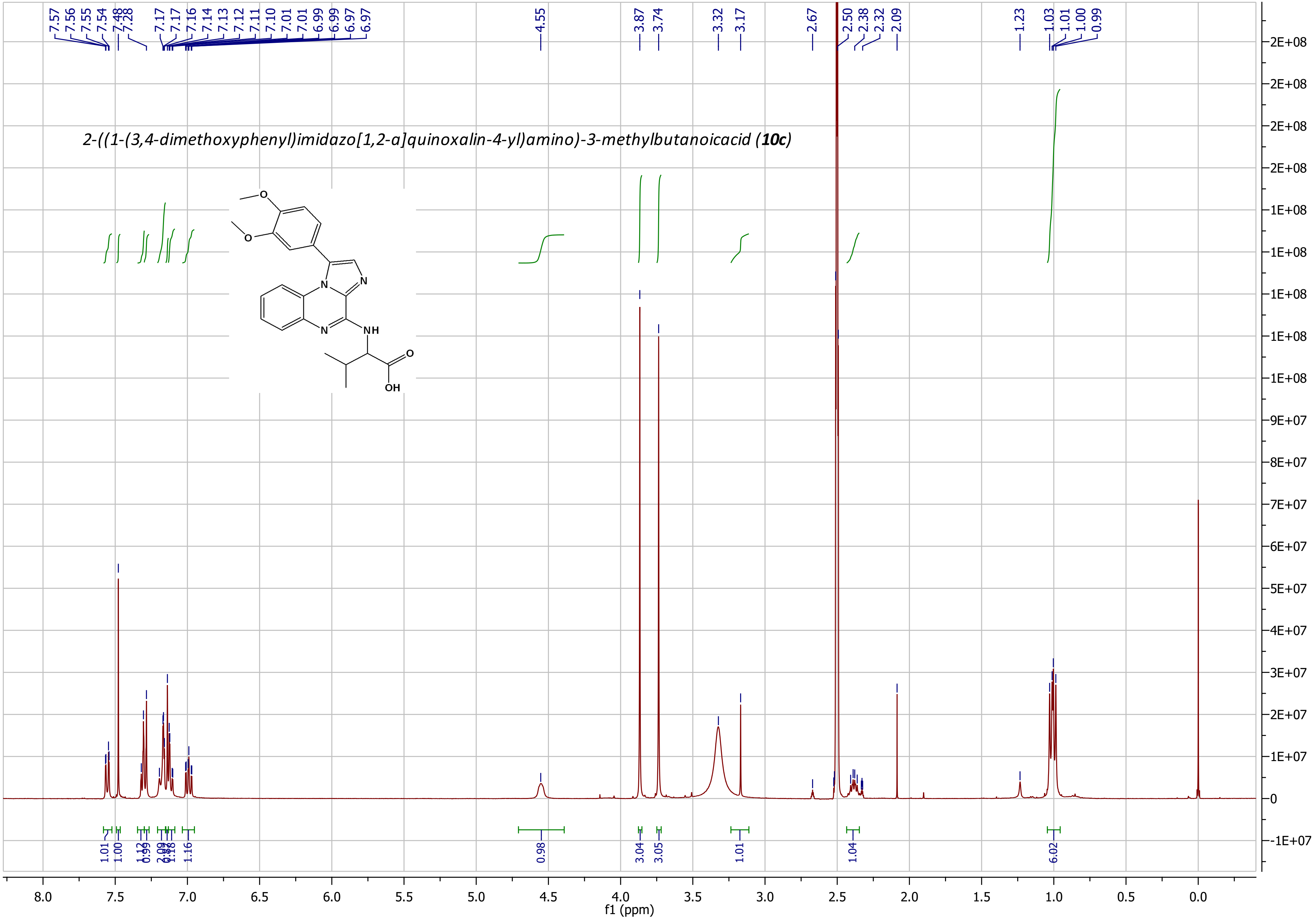
2-((1-(3,4-dimethoxyphenyl)imidazo[1,2-a]quinoxalin-4-yl)amino)-3-methylbutanoicacid (**10c**)
f1 (ppm)

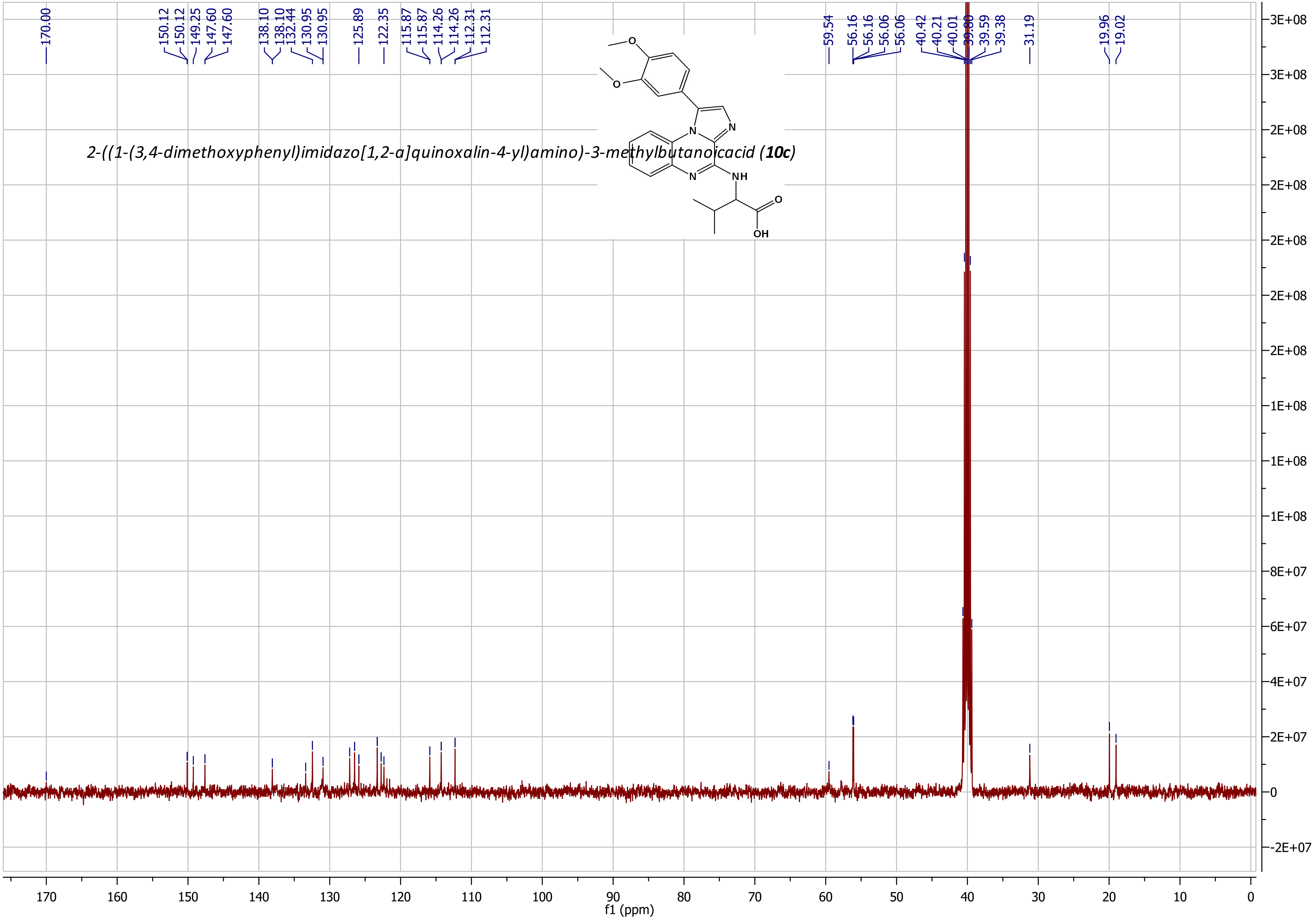

2-((1-(3,4-dimethoxyphenyl)imidazo[1,2-a]quinoxalin-4-yl)amino)-3-methylbutanoicacid (**10c**)
170.00
150.12
150.12
149.25
147.60
147.60
138.10
138.10
132.44
130.95
130.95
125.89
122.35
115.87
115.87
114.26
114.26
112.31
112.31
59.54
56.16
56.16
56.06
56.06
40.42
40.21
40.01
39.80
39.59
39.38
31.19
19.96
19.02
O
O
N
N
N
NH
O
OH
3E+08
3E+08
2E+08
2E+08
2E+08
2E+08
2E+08
1E+08
1E+08
1E+08
8E+07
6E+07
4E+07
2E+07
0
-2E+07
170
160
150
140
130
120
110
100
90
80
70
60
50
40
30
20
10
0
f1 (ppm)

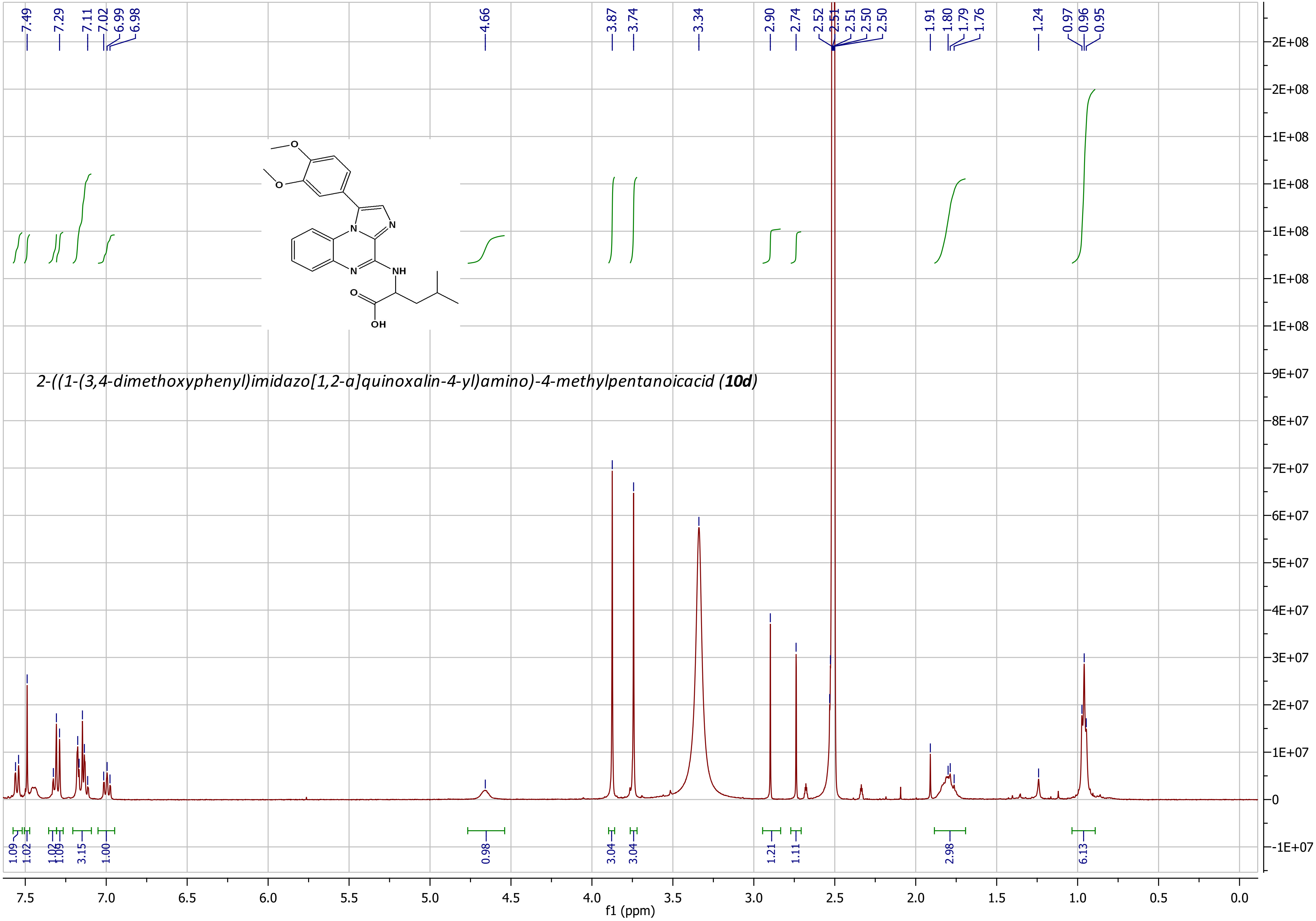

*2-((1-(3,4-dimethoxyphenyl)imidazo[1,2-a]quinoxalin-4-yl)amino)-4-methylpentanoicacid (**10d**)*

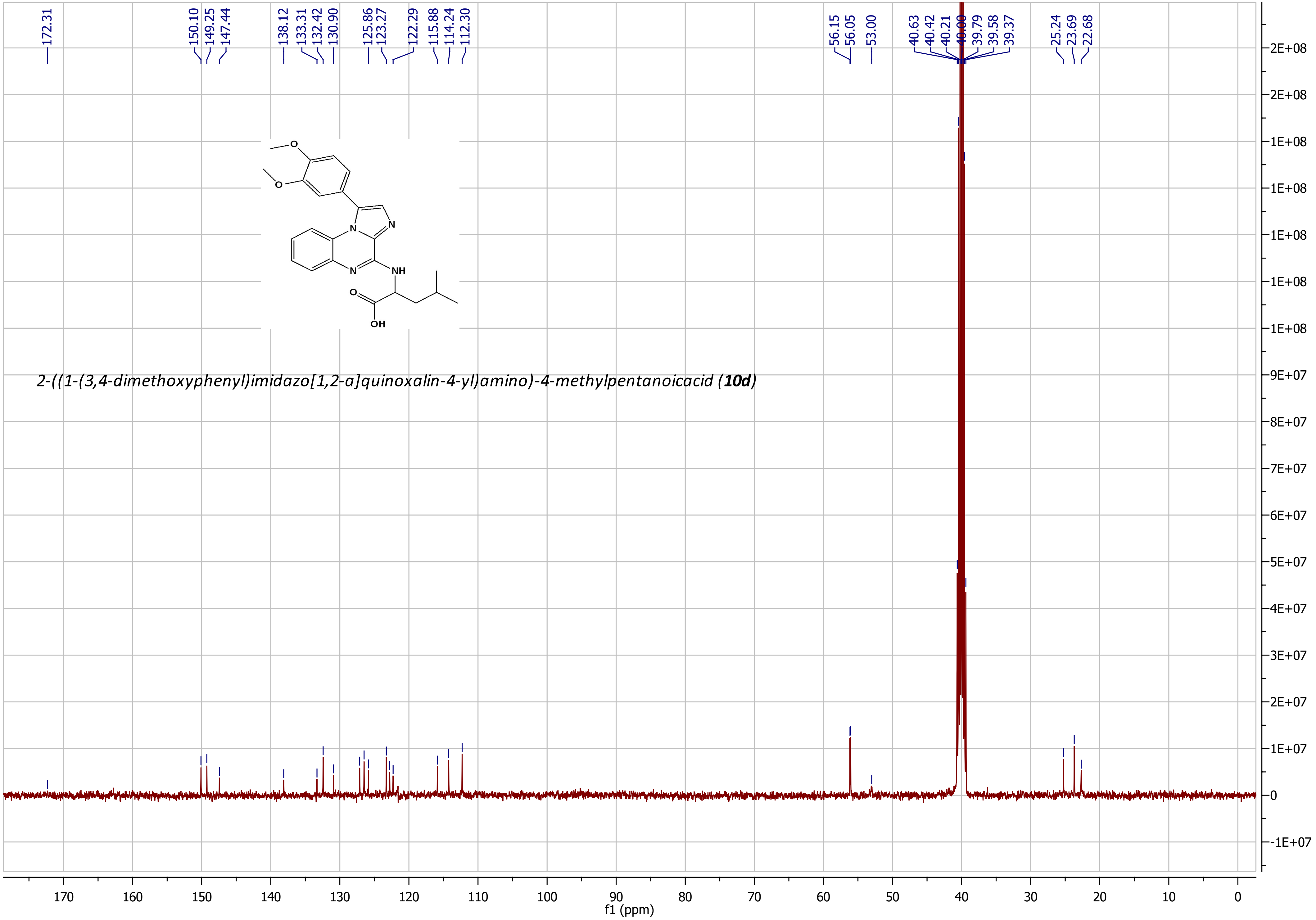

*2-((1-(3,4-dimethoxyphenyl)imidazo[1,2-a]quinoxalin-4-yl)amino)-4-methylpentanoicacid (**10d**)*

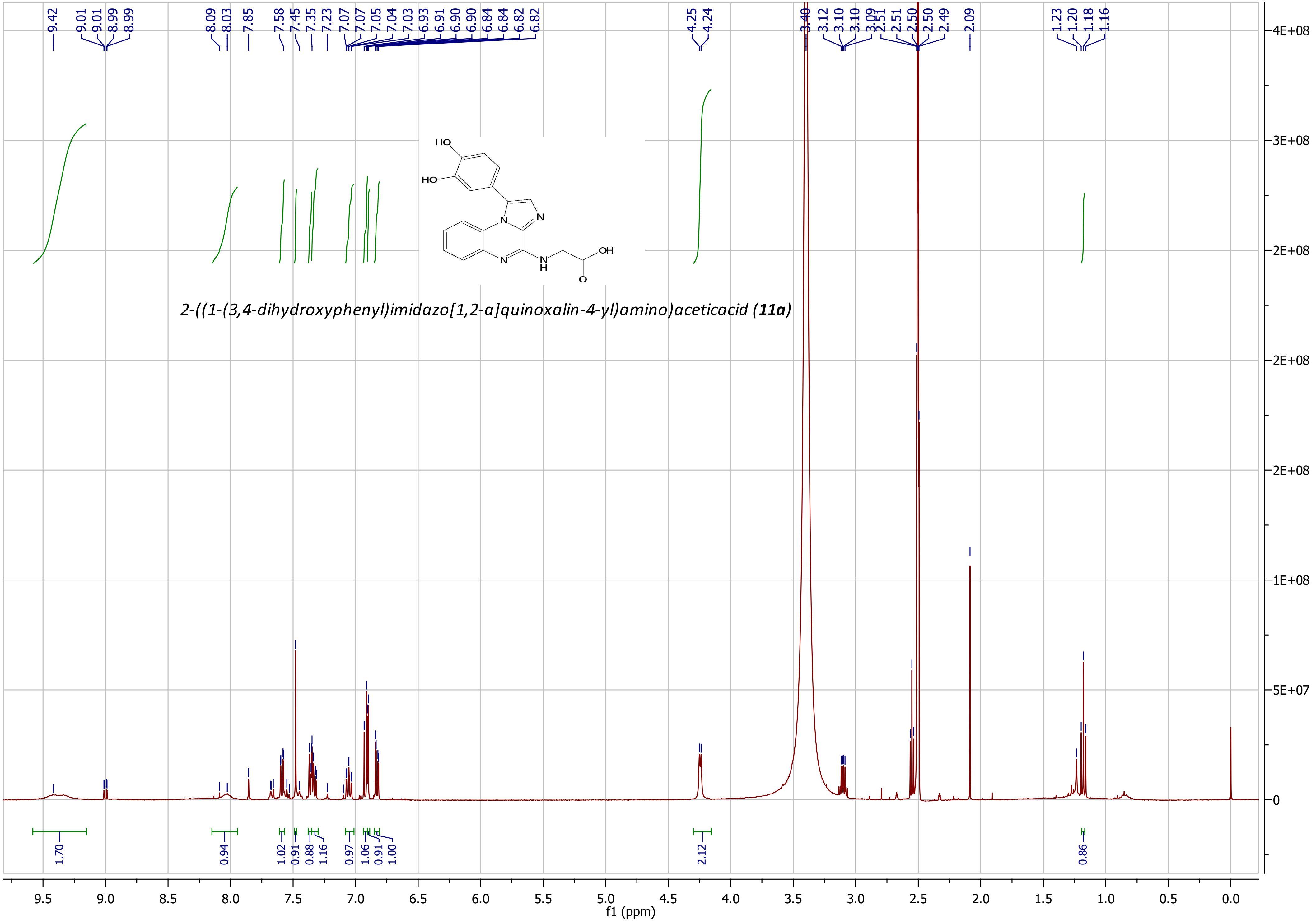
9.42
9.01
9.01
8.99
8.99
8.09
8.03
7.85
7.58
7.45
7.35
7.23
7.07
7.07
7.05
7.04
7.03
6.93
6.91
6.90
6.90
6.84
6.84
6.82
6.82
4.25
4.24
3.40
3.12
3.10
3.10
3.09
2.51
2.51
2.50
2.50
2.49
2.09
1.23
1.20
1.18
1.16
HO
HO
N
N
N
N
H
OH
O
2-((1-(3,4-dihydroxyphenyl)imidazo[1,2-a]quinoxalin-4-yl)amino)aceticacid (**11a**)
1.70
0.94
1.02
0.91
0.88
1.16
0.97
1.06
0.91
1.00
2.12
0.86
4E+08
3E+08
2E+08
2E+08
2E+08
1E+08
5E+07
0
9.5
9.0
8.5
8.0
7.5
7.0
6.5
6.0
5.5
5.0
4.5
4.0
3.5
3.0
2.5
2.0
1.5
1.0
0.5
0.0
f1 (ppm)

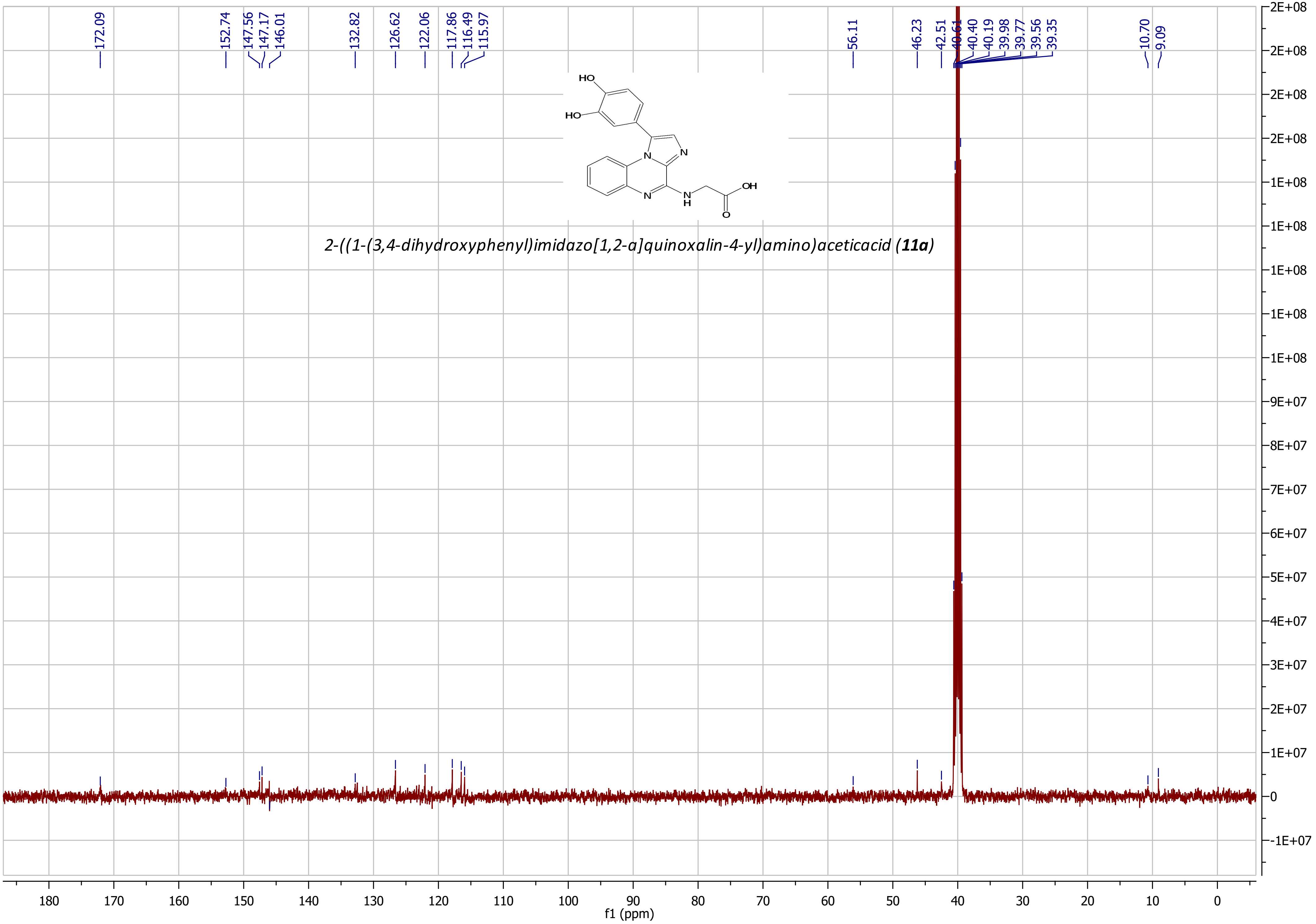

2-((1-(3,4-dihydroxyphenyl)imidazo[1,2-a]quinoxalin-4-yl)amino)aceticacid (***11a***)

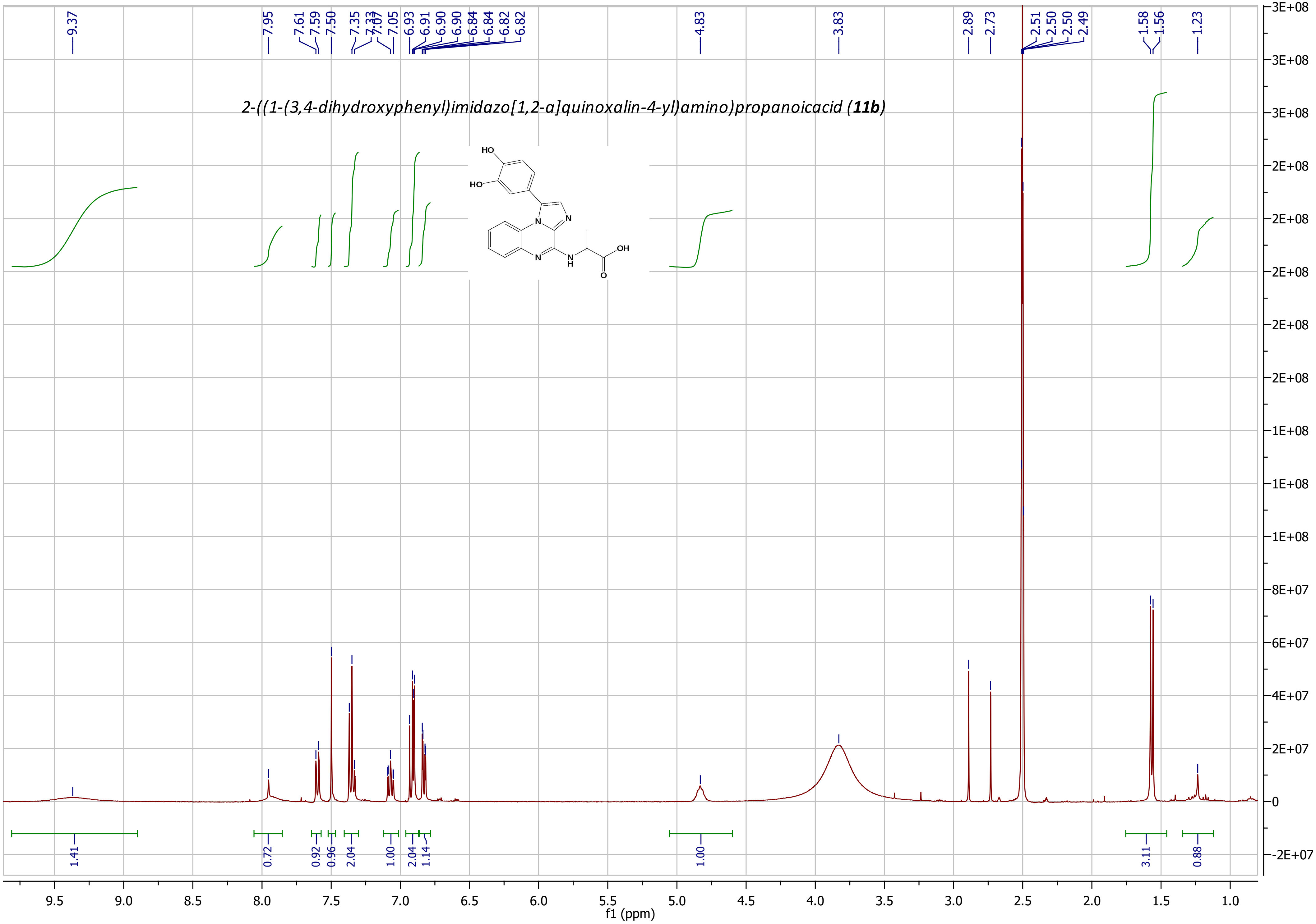

2-((1-(3,4-dihydroxyphenyl)imidazo[1,2-a]quinoxalin-4-yl)amino)propanoicacid (**11b**)
9.37
7.95
7.61
7.59
7.50
7.35
7.33
7.07
7.05
6.93
6.91
6.90
6.90
6.84
6.84
6.82
6.82
4.83
3.83
2.89
2.73
2.51
2.50
2.50
2.49
1.58
1.56
1.23
1.41
0.72
0.92
0.96
2.04
1.00
2.04
1.14
1.00
3.11
0.88
HO
HO
N
N
N
N
H
OH
O
f1 (ppm)

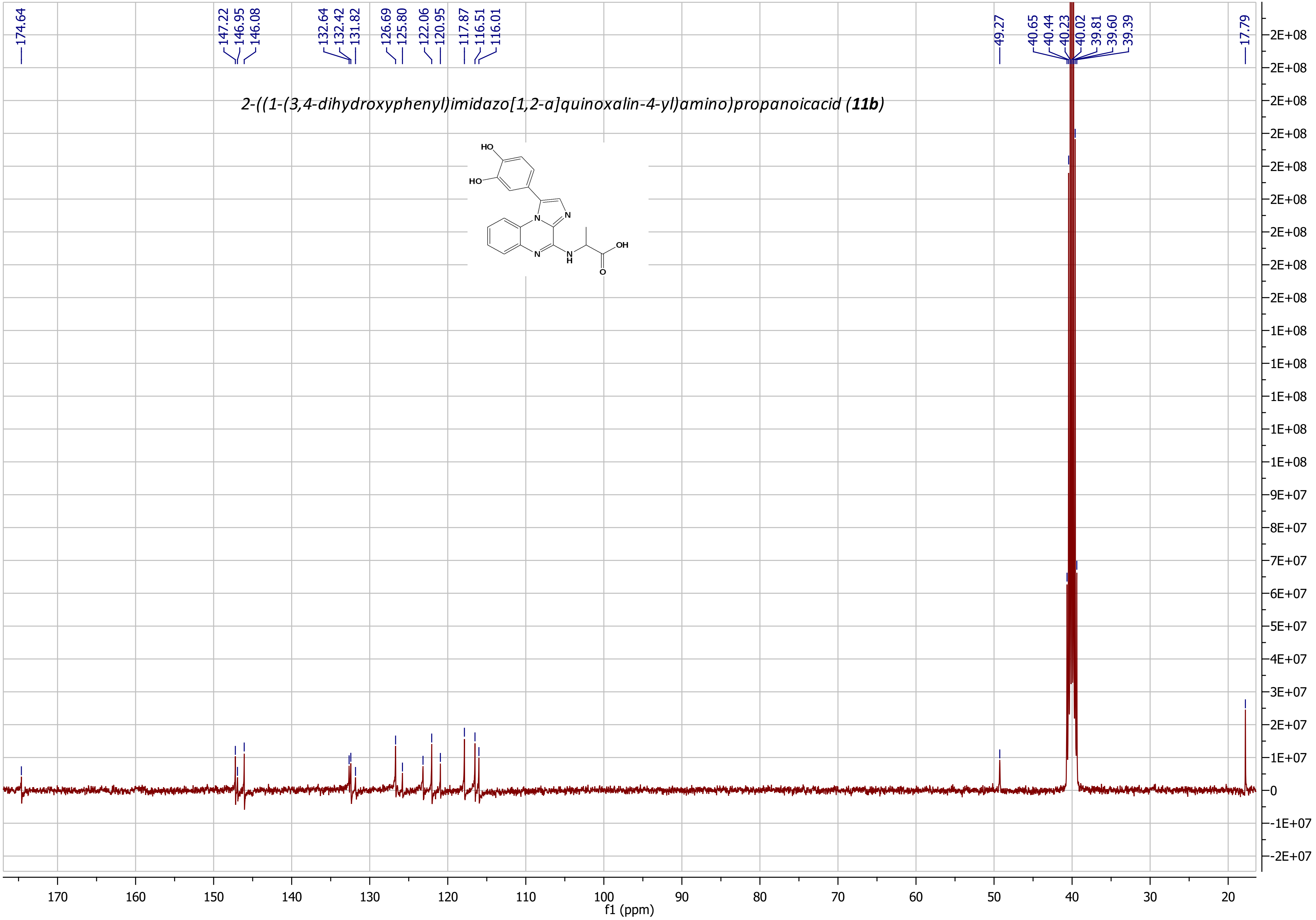
2-((1-(3,4-dihydroxyphenyl)imidazo[1,2-a]quinoxalin-4-yl)amino)propanoicacid (**11b**)
174.64
147.22
146.95
146.08
132.64
132.42
131.82
126.69
125.80
122.06
120.95
117.87
116.51
116.01
49.27
40.65
40.44
40.23
40.02
39.81
39.60
39.39
17.79
HO
HO
N
N
N
N
H
OH
O
f1 (ppm)
170
160
150
140
130
120
110
100
90
80
70
60
50
40
30
20
2E+08
1E+08
9E+07
8E+07
7E+07
6E+07
5E+07
4E+07
3E+07
2E+07
1E+07
0
-1E+07
-2E+07

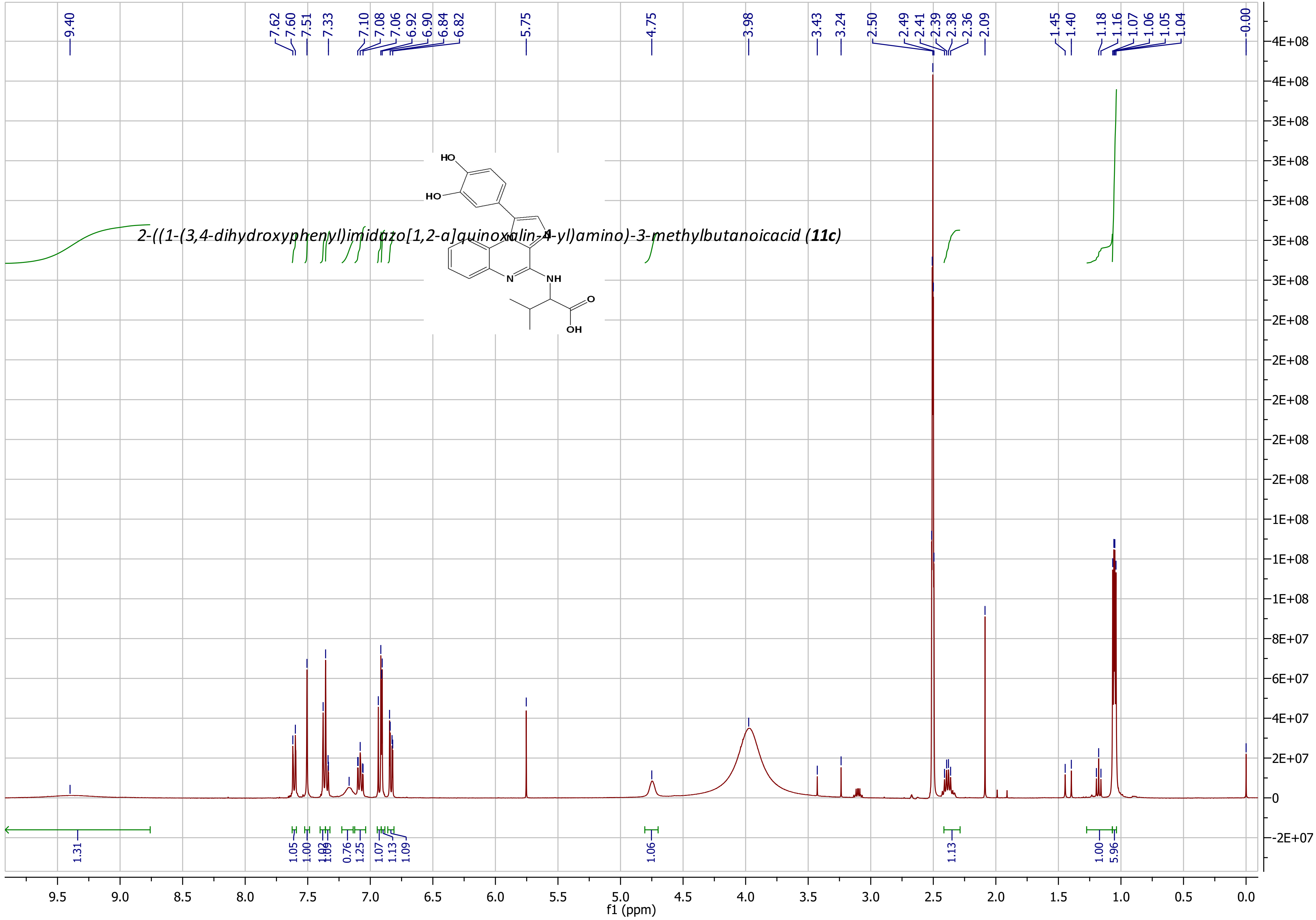

2-((1-(3,4-dihydroxyphenyl)imidazo[1,2-a]quinoxalin-4-yl)amino)-3-methylbutanoicacid (**11c**)

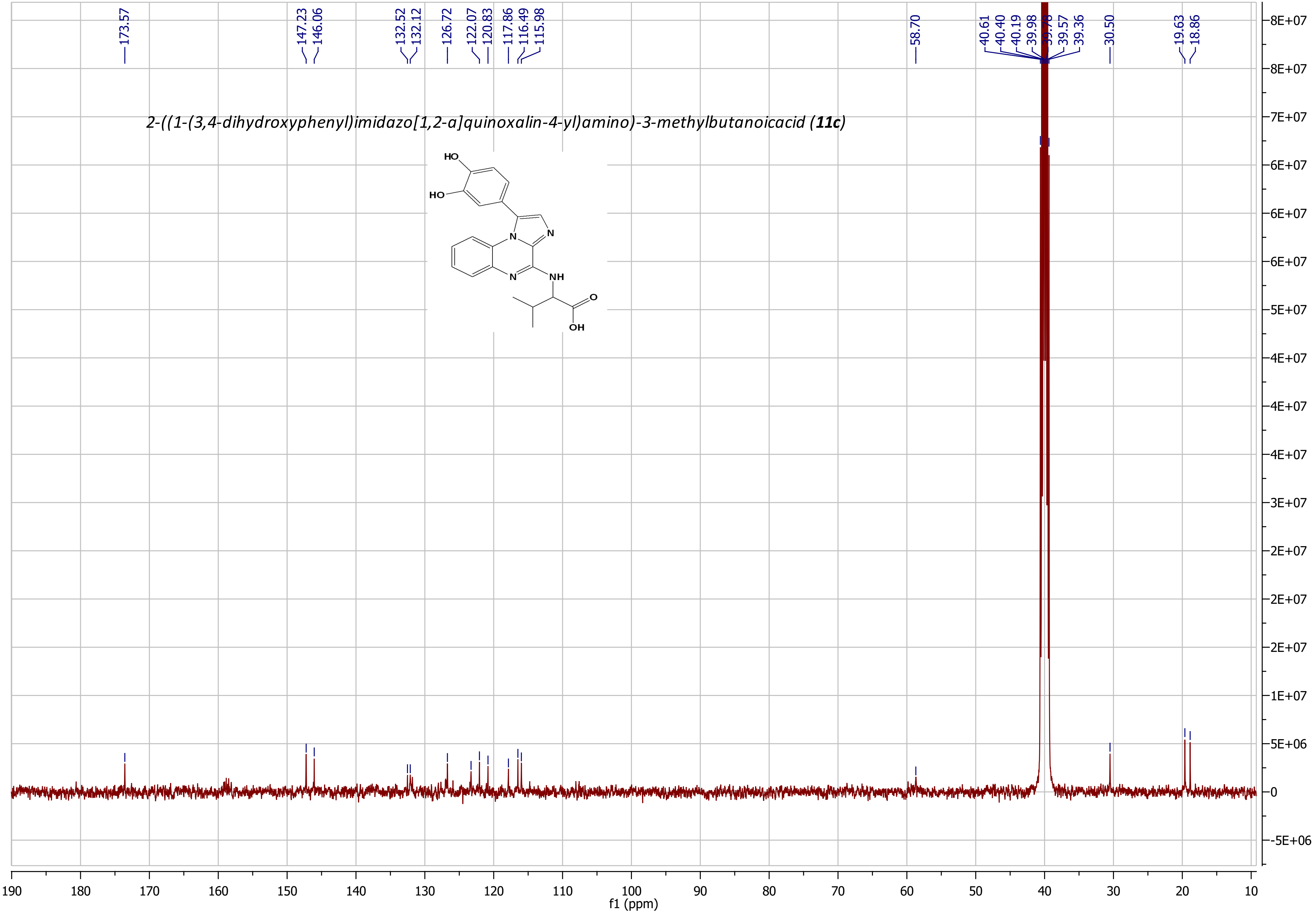
173.57
147.23
146.06
132.52
132.12
126.72
122.07
120.83
117.86
116.49
115.98
58.70
40.61
40.40
40.19
39.98
39.78
39.57
39.36
30.50
19.63
18.86
2-((1-(3,4-dihydroxyphenyl)imidazo[1,2-a]quinoxalin-4-yl)amino)-3-methylbutanoicacid (**11c**)
HO
HO
N
N
N
NH
O
OH
f1 (ppm)

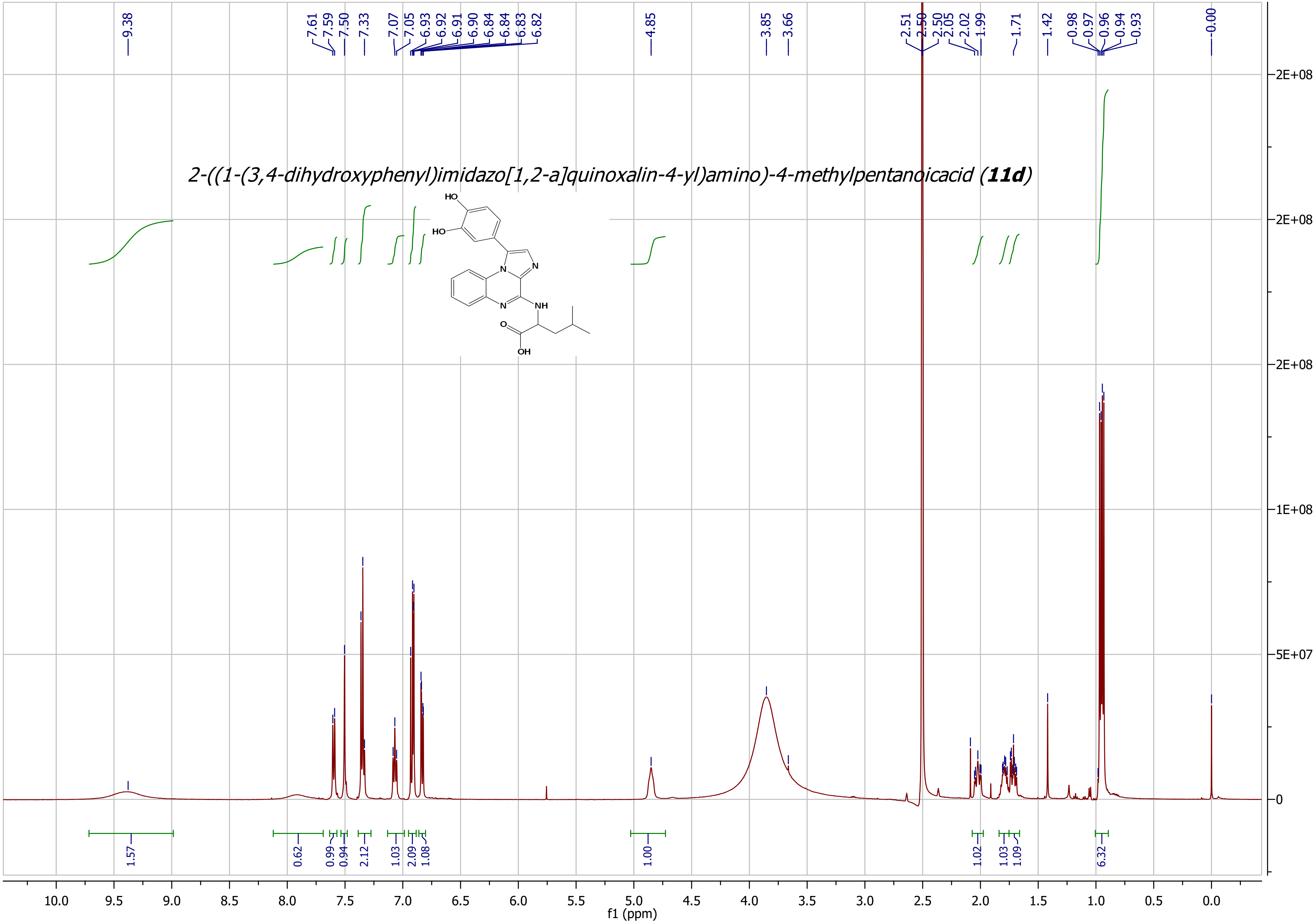
2-((1-(3,4-dihydroxyphenyl)imidazo[1,2-a]quinoxalin-4-yl)amino)-4-methylpentanoicacid (**11d**)
HO
HO
N
N
N
NH
O
OH
9.38
7.61
7.59
7.50
7.33
7.07
7.05
6.93
6.92
6.91
6.90
6.84
6.84
6.83
6.82
4.85
3.85
3.66
2.51
2.50
2.50
2.05
2.02
1.99
1.71
1.42
0.98
0.97
0.96
0.94
0.93
-0.00
1.57
0.62
0.99
0.94
2.12
1.03
2.09
1.08
1.00
1.02
1.03
1.09
6.32
2E+08
2E+08
2E+08
1E+08
5E+07
0
10.0
9.5
9.0
8.5
8.0
7.5
7.0
6.5
6.0
5.5
5.0
4.5
4.0
3.5
3.0
2.5
2.0
1.5
1.0
0.5
0.0
f1 (ppm)

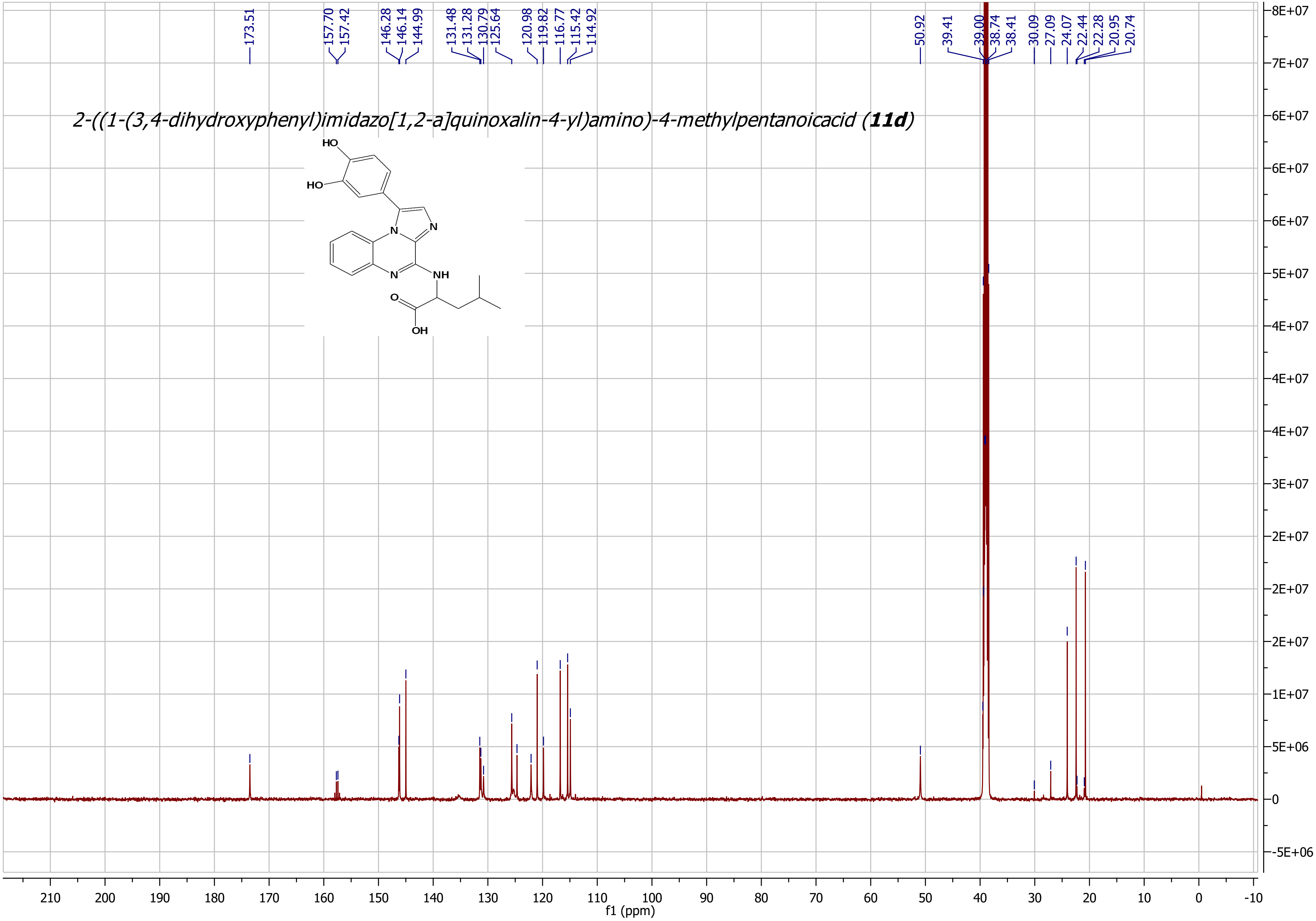
2-((1-(3,4-dihydroxyphenyl)imidazo[1,2-a]quinoxalin-4-yl)amino)-4-methylpentanoicacid (**11d**)
173.51
157.70
157.42
146.28
146.14
144.99
131.48
131.28
130.79
125.64
120.98
119.82
116.77
115.42
114.92
50.92
39.41
39.00
38.74
38.41
30.09
27.09
24.07
22.44
22.28
20.95
20.74
HO
HO
N
N
N
NH
O
OH
f1 (ppm)

*6-amino-2-((1-(3,4-dihydroxyphenyl)imidazo[1,2-a]quinoxalin-4-yl)amino)hexanoicacid (**11e**)*

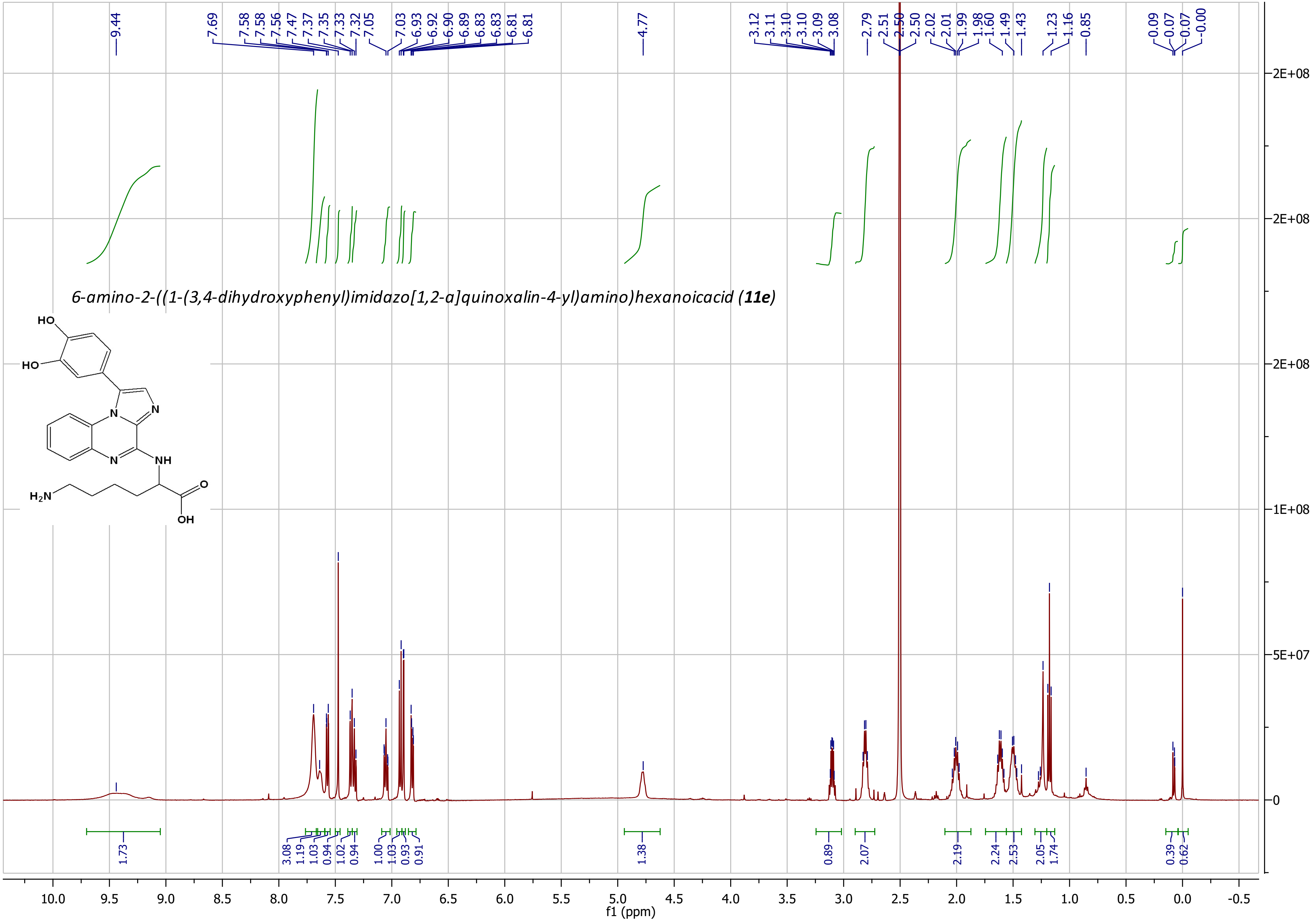

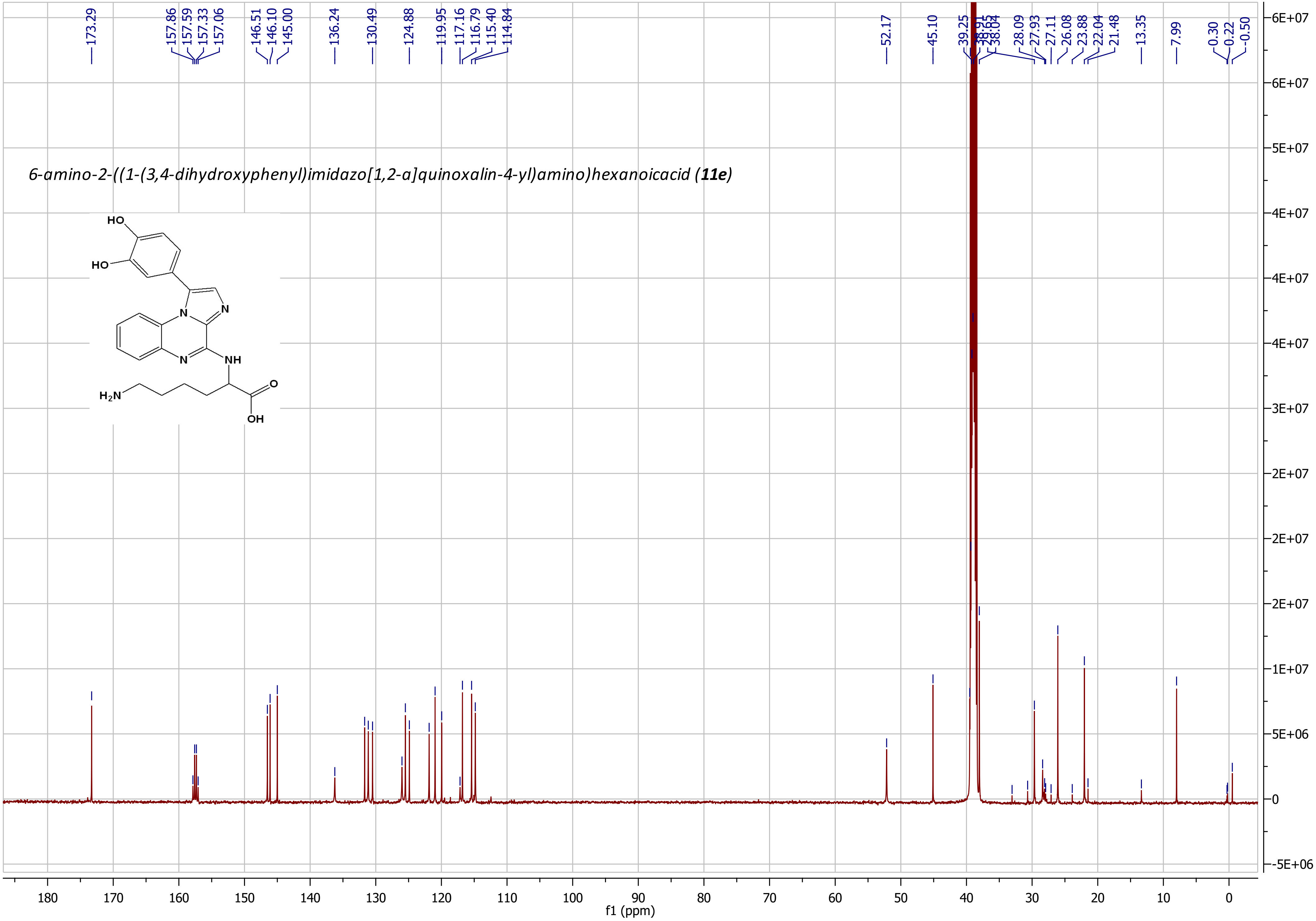
6-amino-2-((1-(3,4-dihydroxyphenyl)imidazo[1,2-a]quinoxalin-4-yl)amino)hexanoicacid (11e)
HO
HO
N
N
N
NH
H2N
O
OH
173.29
157.86
157.59
157.33
157.06
146.51
146.10
145.00
136.24
130.49
124.88
119.95
117.16
116.79
115.40
114.84
52.17
45.10
39.25
38.04
28.09
27.93
27.11
26.08
23.88
22.04
21.48
13.35
7.99
0.30
0.22
-0.50
f1 (ppm)

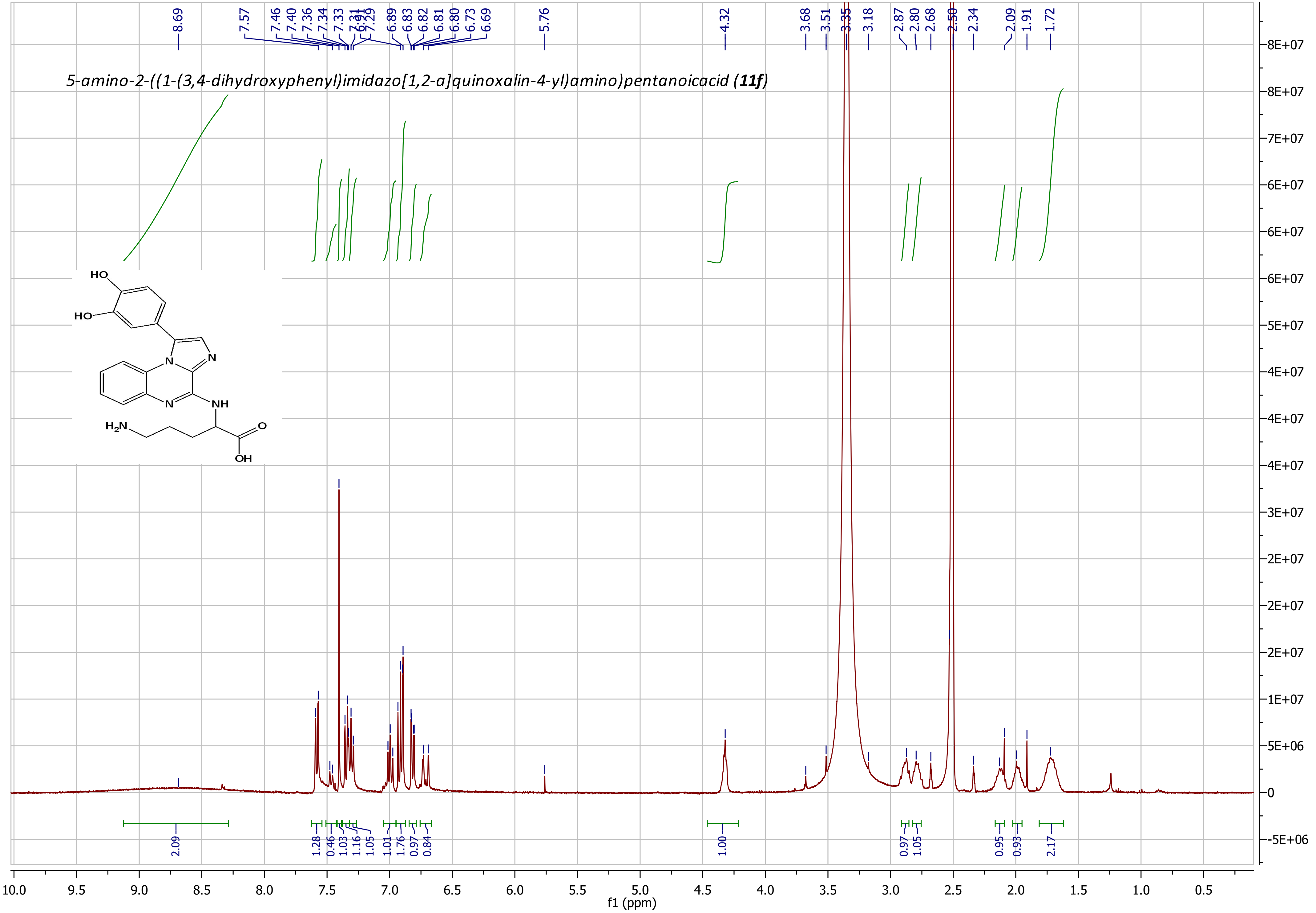
5-amino-2-((1-(3,4-dihydroxyphenyl)imidazo[1,2-a]quinoxalin-4-yl)amino)pentanoicacid (**11f**)
8.69
7.57
7.46
7.40
7.36
7.34
7.33
7.31
6.91
7.29
6.89
6.83
6.82
6.81
6.80
6.73
6.69
5.76
4.32
3.68
3.51
3.35
3.18
2.87
2.80
2.68
2.50
2.34
2.09
1.91
1.72
2.09
1.28
0.46
1.03
1.16
1.05
1.01
1.76
0.97
0.84
1.00
0.97
1.05
0.95
0.93
2.17
HO
HO
N
N
N
NH
H_2N
O
OH
f1 (ppm)

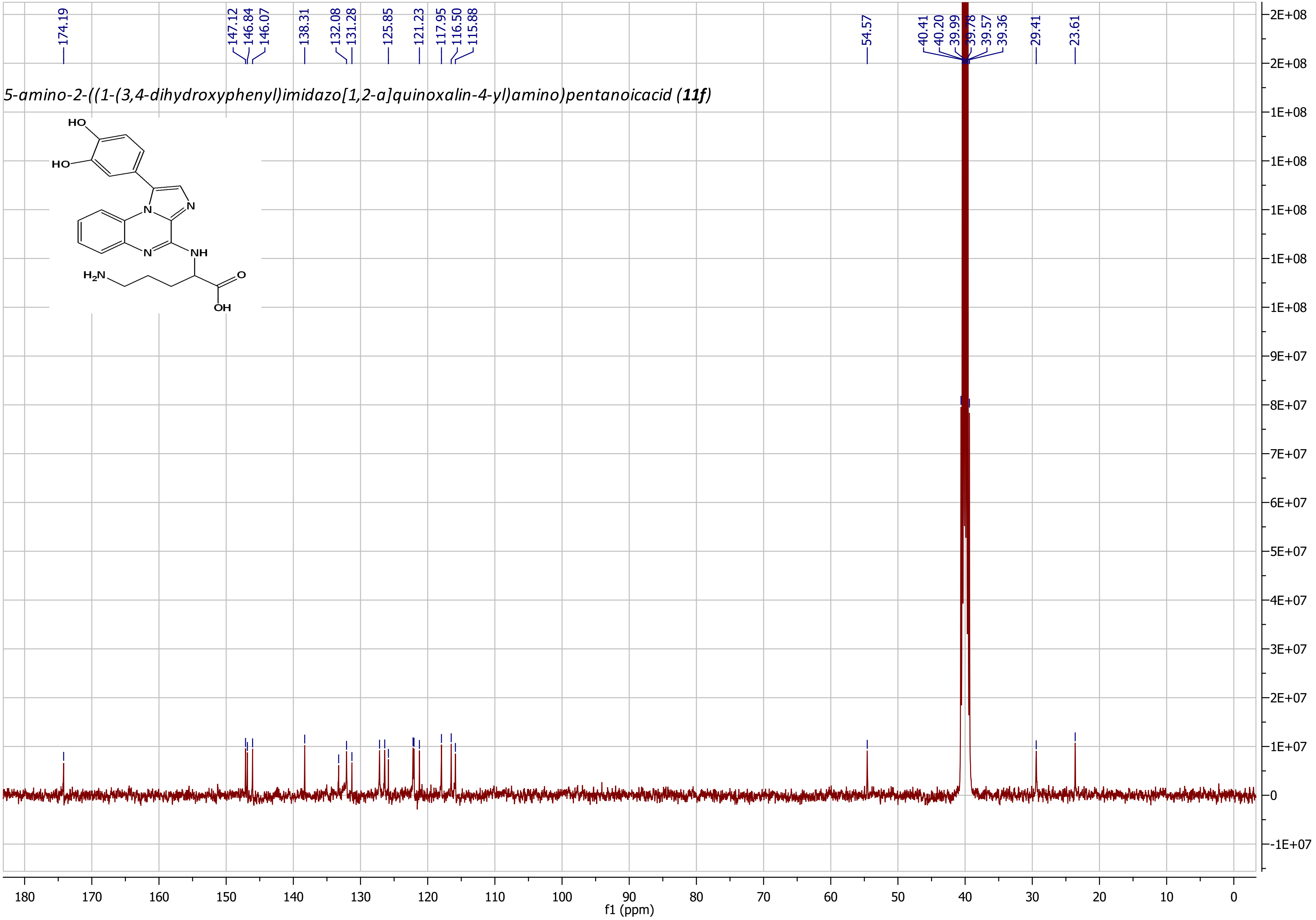
5-amino-2-((1-(3,4-dihydroxyphenyl)imidazo[1,2-a]quinoxalin-4-yl)amino)pentanoicacid (**11f**)
174.19
147.12
146.84
146.07
138.31
132.08
131.28
125.85
121.23
117.95
116.50
115.88
54.57
40.41
40.20
39.99
39.78
39.57
39.36
29.41
23.61
HO
HO
N
N
N
NH
H_2N
O
OH
f1 (ppm)

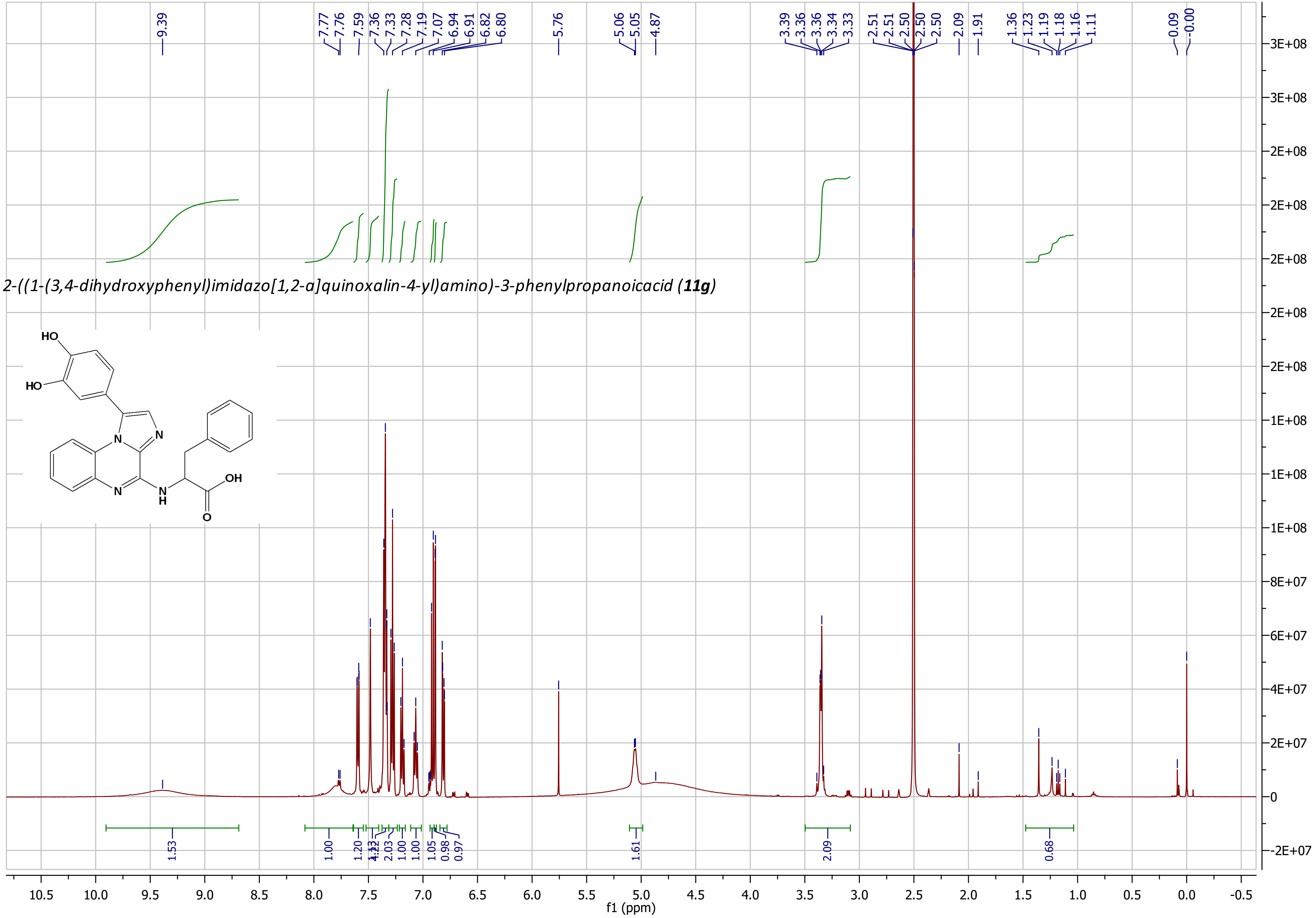

*2-((1-(3,4-dihydroxyphenyl)imidazo[1,2-a]quinoxalin-4-yl)amino)-3-phenylpropanoicacid (**11g**)*

*2-((1-(3,4-dihydroxyphenyl)imidazo[1,2-a]quinoxalin-4-yl)amino)-3-phenylpropanoicacid (**11g**)*

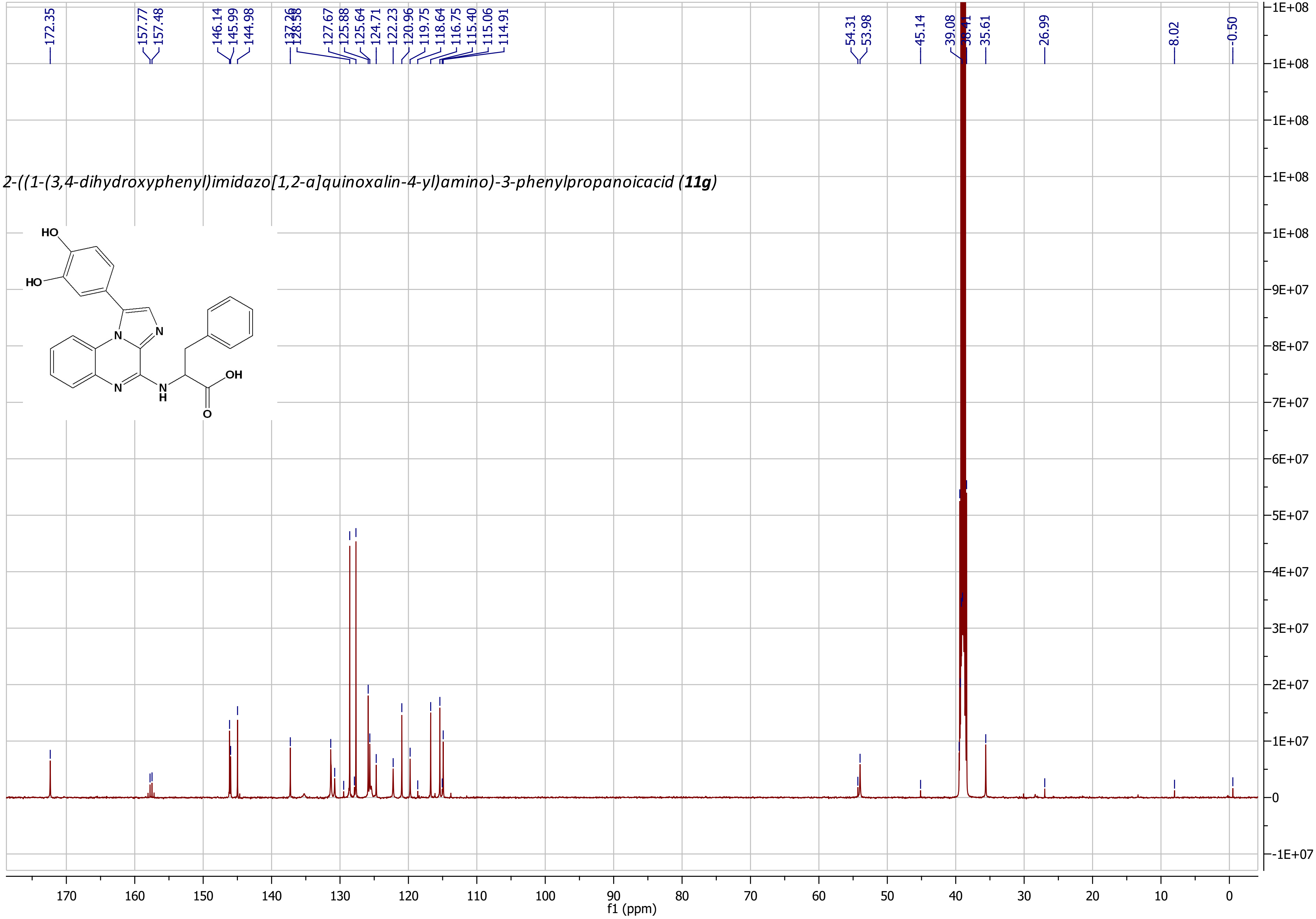

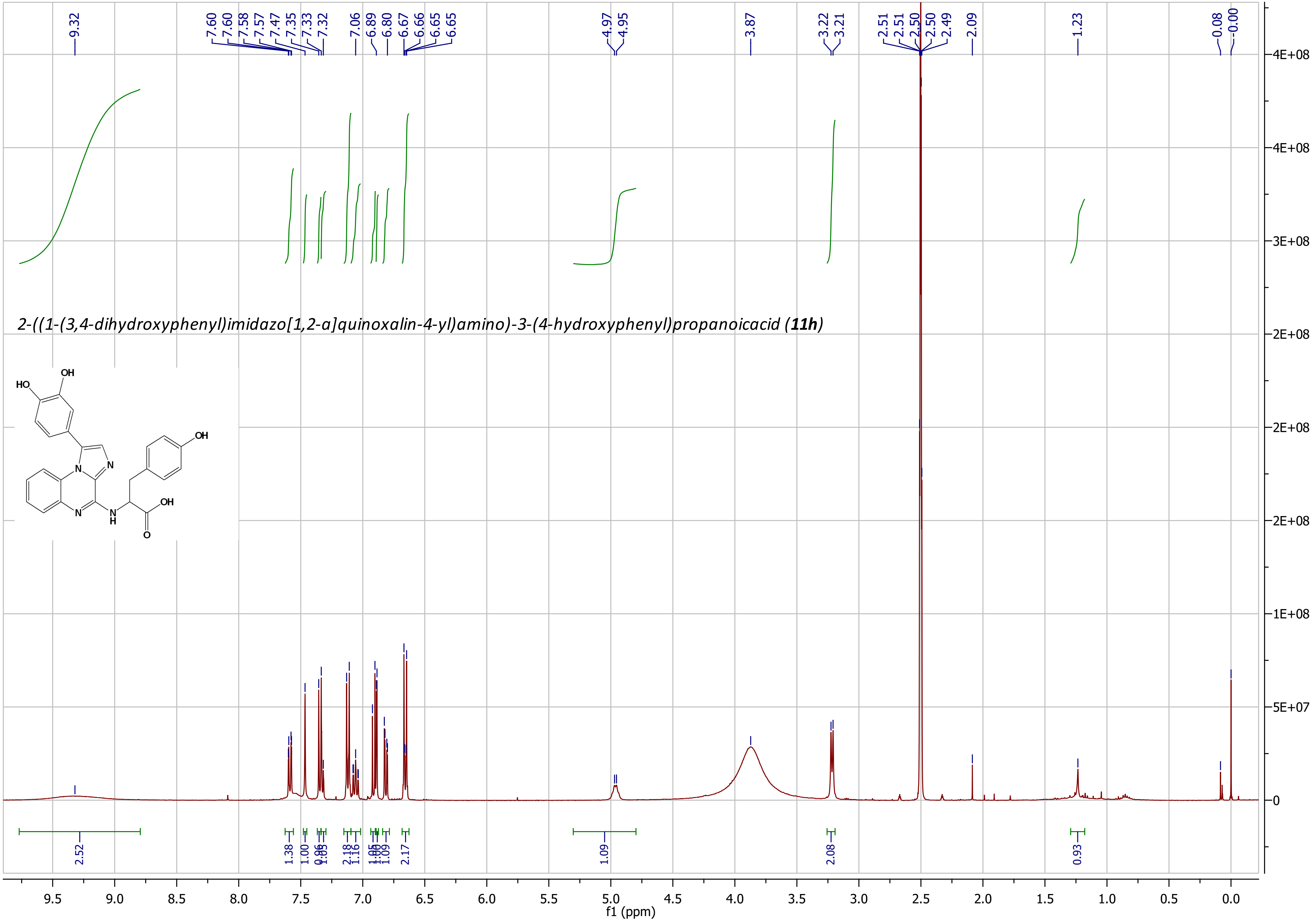
2-((1-(3,4-dihydroxyphenyl)imidazo[1,2-a]quinoxalin-4-yl)amino)-3-(4-hydroxyphenyl)propanoicacid (**11h**)
HO
OH
OH
N
N
N
N
H
OH
O
9.32
7.60
7.60
7.58
7.57
7.47
7.35
7.33
7.32
7.06
6.89
6.80
6.67
6.66
6.65
6.65
4.97
4.95
3.87
3.22
3.21
2.51
2.51
2.50
2.50
2.49
2.09
1.23
0.08
0.00
2.52
1.38
1.00
0.96
1.05
2.18
1.16
1.05
1.00
1.09
2.17
1.09
2.08
0.93
4E+08
4E+08
3E+08
2E+08
2E+08
2E+08
1E+08
5E+07
0
9.5
9.0
8.5
8.0
7.5
7.0
6.5
6.0
5.5
5.0
4.5
4.0
3.5
3.0
2.5
2.0
1.5
1.0
0.5
0.0
f1 (ppm)

*2-((1-(3,4-dihydroxyphenyl)imidazo[1,2-a]quinoxalin-4-yl)amino)-3-(4-hydroxyphenyl)propanoicacid (**11h**)*

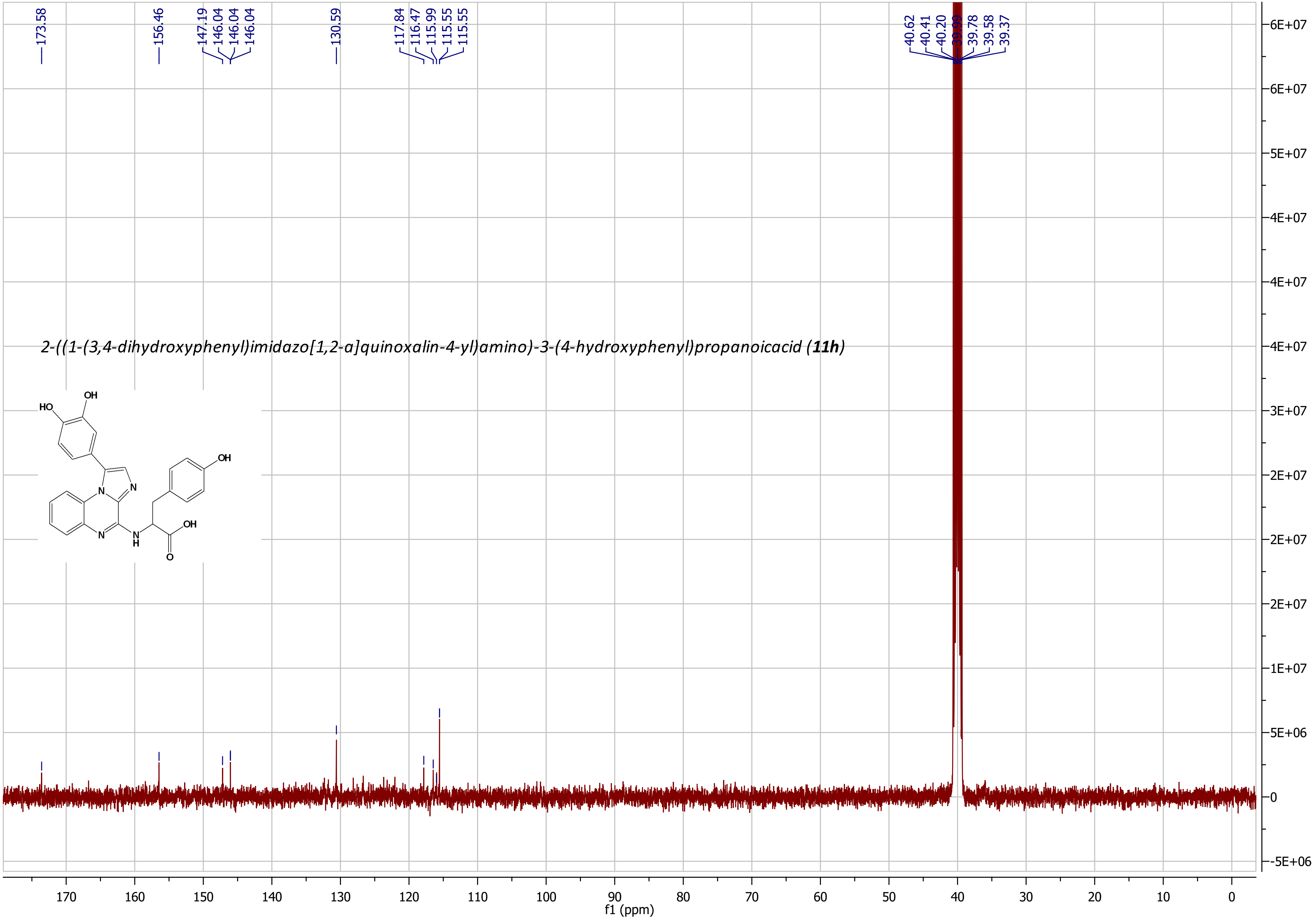

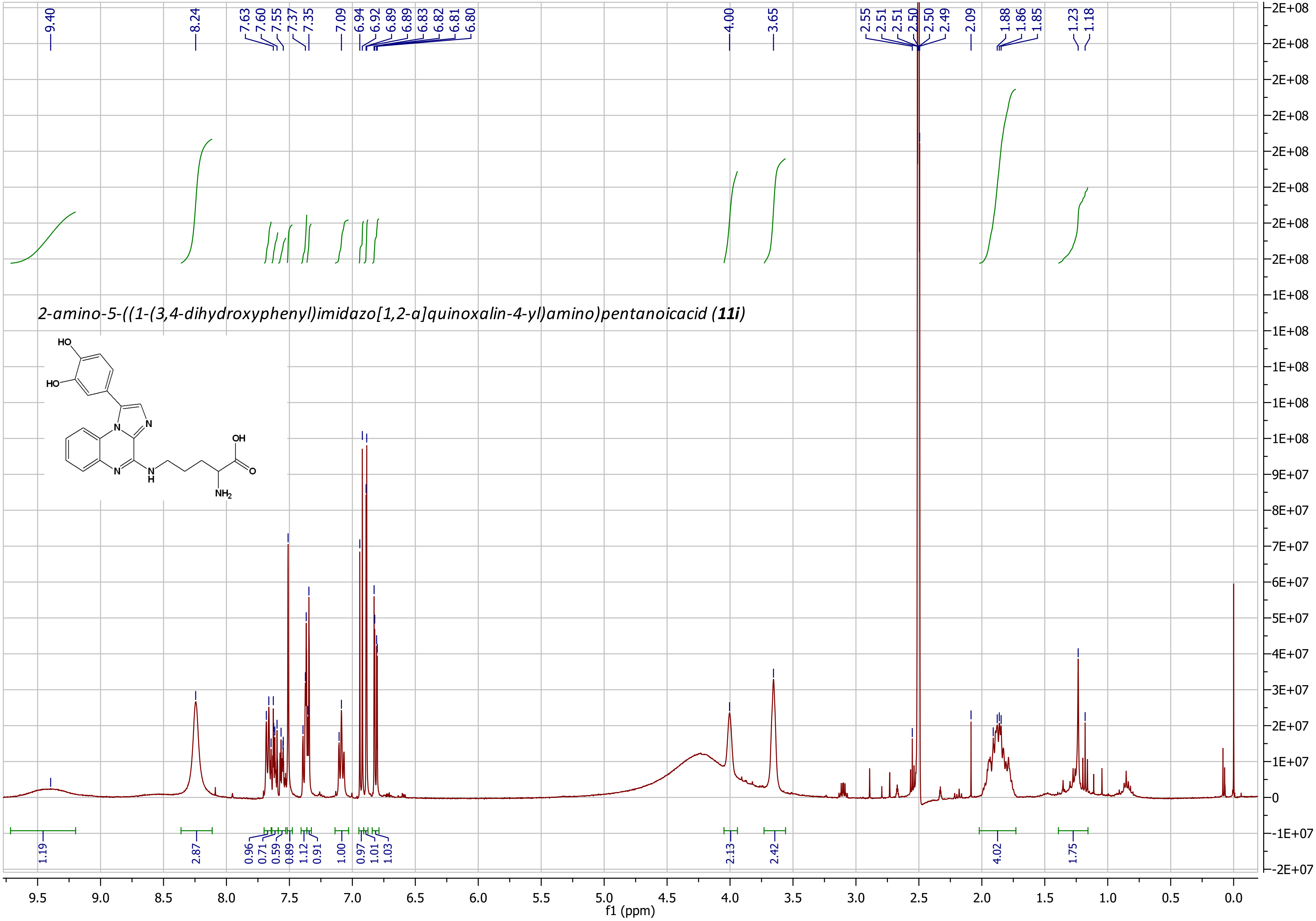
2-amino-5-((1-(3,4-dihydroxyphenyl)imidazo[1,2-a]quinoxalin-4-yl)amino)pentanoicacid (**11i**)
HO
HO
N
N
N
N
H
OH
O
NH2
9.40
8.24
7.63
7.60
7.55
7.37
7.35
7.09
6.94
6.92
6.89
6.89
6.83
6.82
6.81
6.80
4.00
3.65
2.55
2.51
2.51
2.50
2.50
2.49
2.09
1.88
1.86
1.85
1.23
1.18
1.19
2.87
0.96
0.71
0.59
0.89
1.12
0.91
1.00
0.97
1.01
1.03
2.13
2.42
4.02
1.75
f1 (ppm)
9.5
9.0
8.5
8.0
7.5
7.0
6.5
6.0
5.5
5.0
4.5
4.0
3.5
3.0
2.5
2.0
1.5
1.0
0.5
0.0
2E+08
1E+08
9E+07
8E+07
7E+07
6E+07
5E+07
4E+07
3E+07
2E+07
1E+07
0
-1E+07
-2E+07

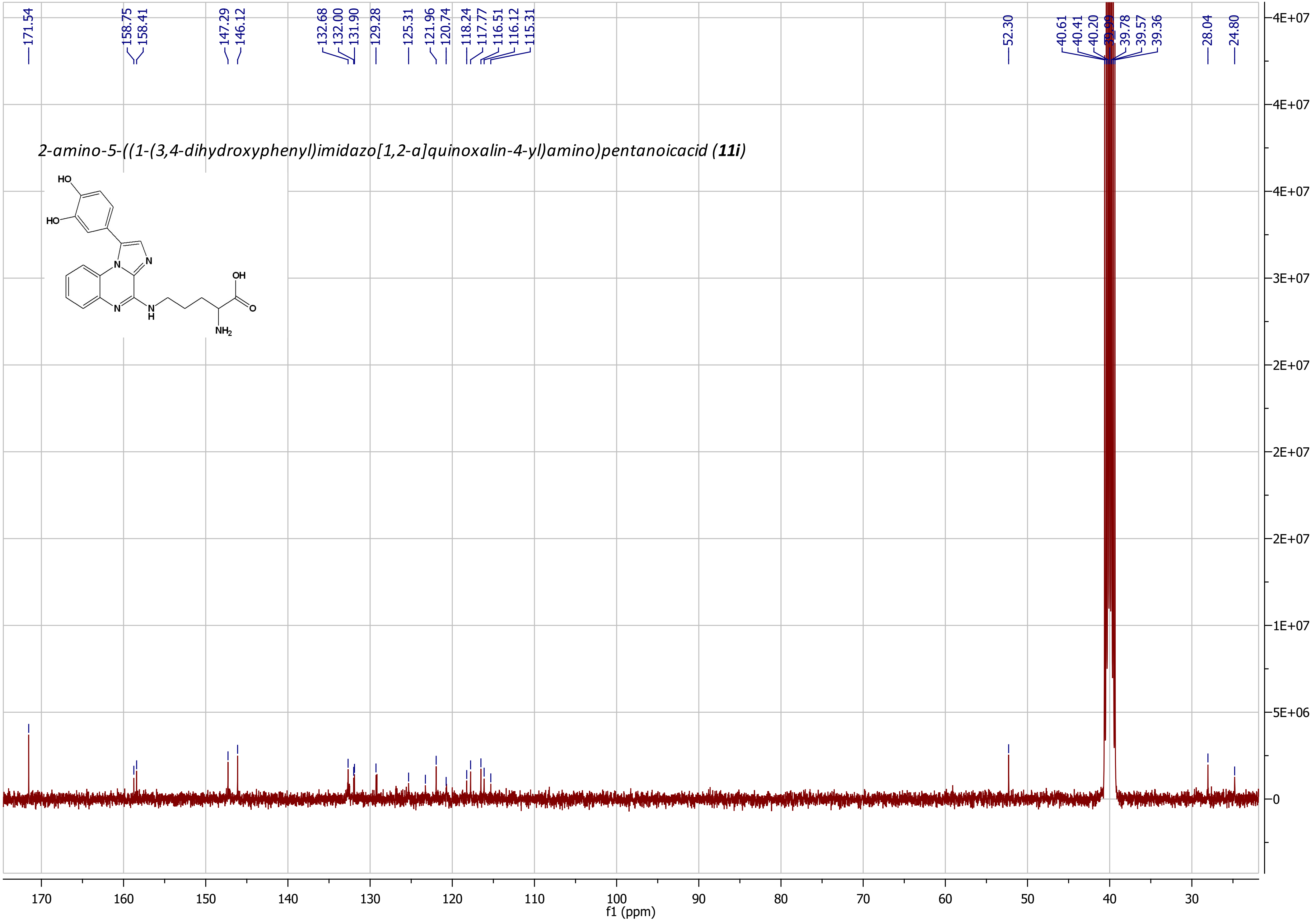
2-amino-5-((1-(3,4-dihydroxyphenyl)imidazo[1,2-a]quinoxalin-4-yl)amino)pentanoicacid (**11i**)
HO
HO
N
N
N
N
H
OH
O
NH2
171.54
158.75
158.41
147.29
146.12
132.68
132.00
131.90
129.28
125.31
121.96
120.74
118.24
117.77
116.51
116.12
115.31
52.30
40.61
40.41
40.20
39.99
39.78
39.57
39.36
28.04
24.80
4E+07
4E+07
4E+07
3E+07
2E+07
2E+07
2E+07
1E+07
5E+06
0
170
160
150
140
130
120
110
100
90
80
70
60
50
40
30
f1 (ppm)
